# Fruits from *Rosa roxburghii*: A Valuable Bioresource of Potent Radical Scavengers and Novel Ursane-Type Triterpenoids

**DOI:** 10.1021/acsomega.4c04893

**Published:** 2024-08-27

**Authors:** Yang Yu, Jing Wu, Mei-Fen Bao, Liu Yang, Zhi-Lin Cai, Johann Schinnerl, Xiang-Hai Cai

**Affiliations:** † State Key Laboratory of Phytochemistry and Plant Resources in West China, Kunming Institute of Botany, Chinese Academy of Sciences, Kunming 650201, People’s Republic of China; ‡ Zhongzhihao Cili Industrial Development (Guizhou) Co., LTD., Panzhou 553599, People’s Republic of China; § Department of Botany and Biodiversity Research, University of Vienna, Rennweg 14, A-1030 Vienna, Austria

## Abstract

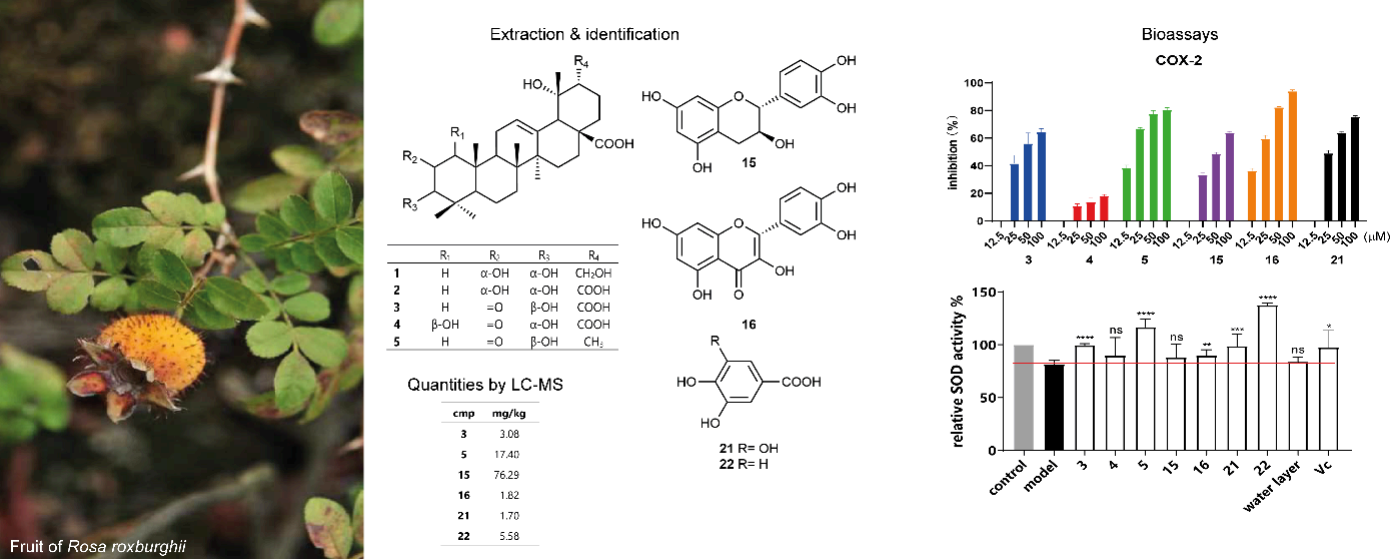

Four new ursane-type triterpenoids named rosaroxine A–D and 21 known compounds were identified from *Rosa roxburghii* fruits. The structures of all compounds were established by 1D and 2D NMR spectroscopy and mass spectrometry. The phenolics catechin (EC_50_ 13.4 μM), quercetin (13.1 μM), gallic acid (10.0 μM), and protocatechuic acid (15.2 μM) were identified as powerful *in vitro* antioxidants with EC_50_ values lower than ascorbic acid (31.3 μM). The triterpenoids rosaroxine C (EC_50_ 37.4; 40.3 μM) and 2-oxo-pomolic acid (16.6; 28.2) and the phenolics catechin (53.3; 29.0), quercetin (18.8; 33.1), and gallic acid (26.3; 40.0) exerted partly higher activities in the cyclo-oxygenase (COX 1/2) assay than the positive control acetaminophen (EC_50_ 45.0; >100 μM). The triterpenoids rosaroxine C and 2-oxo-pomolic acid also performed well in the anti-aging assay using HaCaT cells. Quantification of the bioactive compounds by LC-MS revealed concentrations of 3.08 mg kg^–1^ rosaroxine C, 17.40 mg kg^–1^ 2-oxo-pomolic acid, 76.29 mg kg^–1^ catechin, and 5.58 mg kg^–1^ protocatechuic acid in the dried fruits. Overall, this work provides detailed phytochemical information, and the results from the accomplished bioassay point toward health promoting properties of these fruits.

## 1 Introduction

*Rosa roxburghii* Tratt. (Rosaceae), or Cili, is widely distributed in the southwest of China, particularly in the provinces Guizhou and Yunnan. The fruits of this plant species play an important role in the daily diet of local people, and more than one hundred food and health products from Cili are manufactured. Despite its economic importance in the above-mentioned provinces, detailed phytochemical data about the fruits of this plant are scant. Hydrolyzable tannins,^1^ polyphenols,^2^ and triterpenoids^3^ were described in the past decade. Recently, ascorbic acid derivatives together with flavonoid derivatives were reported.^4^ Performed bioassays of either extracts or single compounds of *R*. *roxburghii* exhibited radioprotective properties^5^ and also radical scavenging activities.^6^ The polysaccharide fraction from *R. roxburghii* showed neurotrophic activity^7^, and Ellagitannins from *R. roxburghii* are able to suppress poly(I:C)-induced IL-8 production in human keratinocytes.^8^ Extracts from fruits of *R. roxburghii* also exerted lipid lowering effects in hyperlipidemic rats and antidiabetic effects, influencing the LDL and HDL as well as the cholesterol levels.

Since most of these reported bioactivities were assessed by using either crude extracts or not well separated fractions of these fruits, we performed a detailed phytochemical analysis of the crude methanolic fruit extract and identified 25 compounds. In addition to the identification of these compounds, we assessed potential antioxidative and anti-inflammatory properties of selected compounds and quantified them in the fruits in order to provide as much information as possible for the use of these fruits.

## 2 Results and Discussion

### 2.1 Structure Elucidation

**Figure 1:**
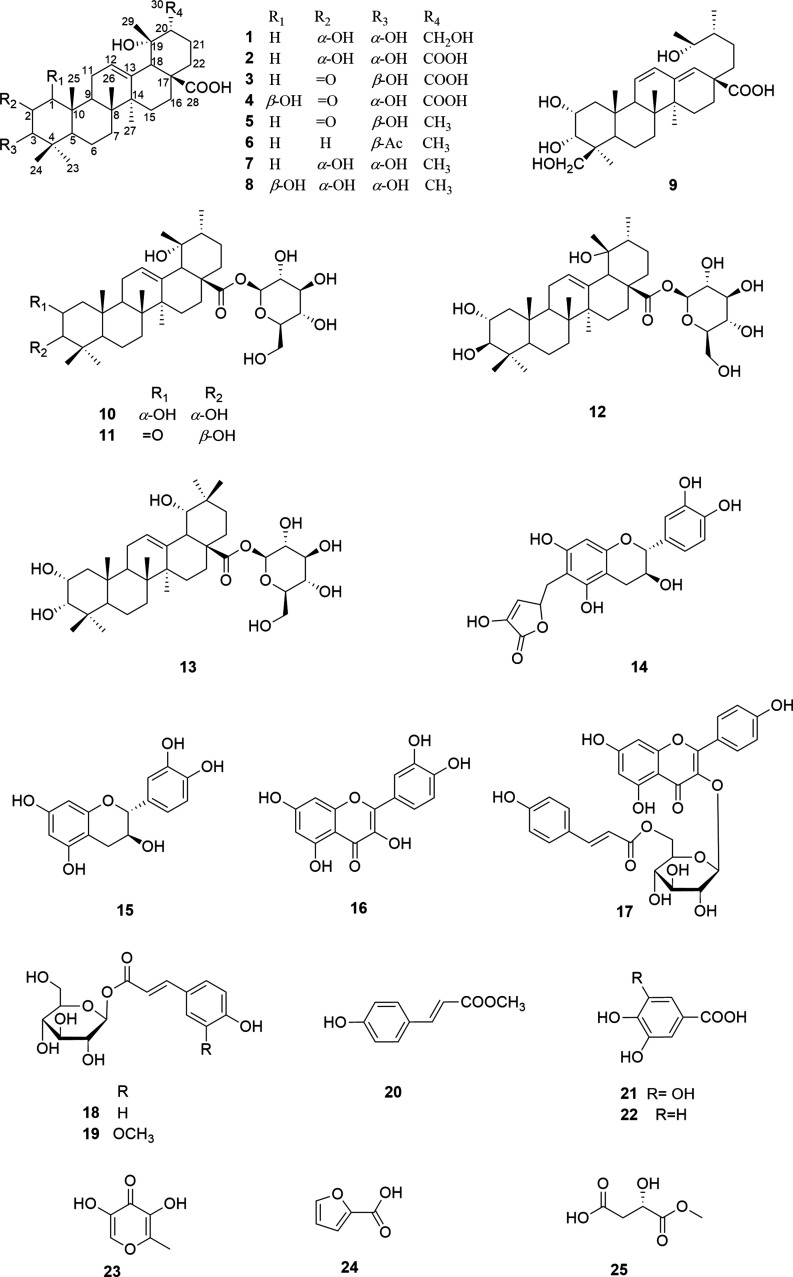
Chemical structures of compounds isolated from *Rosa roxburghii* fruits.

**Figure 2:**
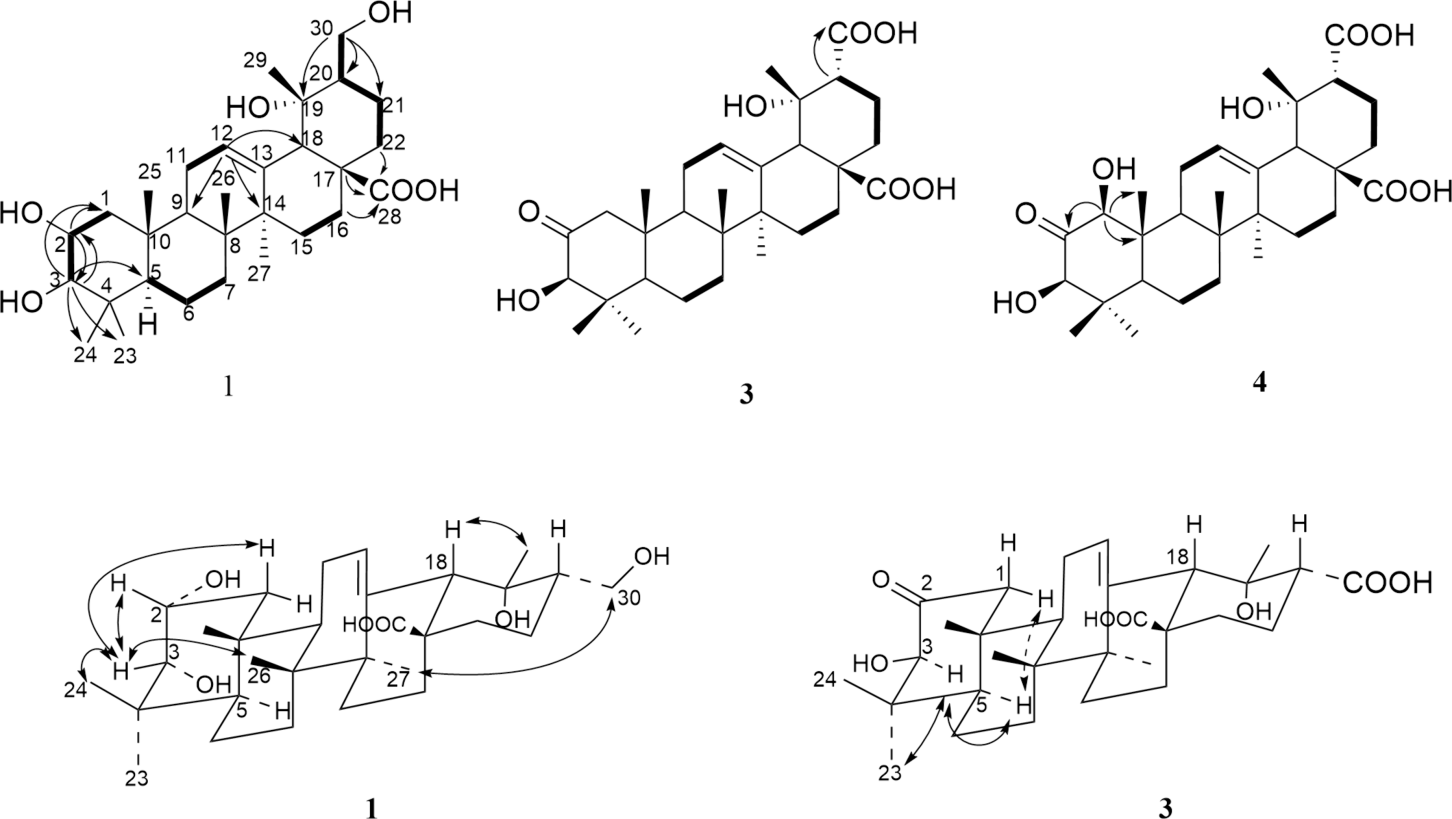
Key HMBC and ^1^H–^1^H COSY correlations of rosaroxine A (**1**), rosaroxine C (**3**), and rosaroxine D (**4**). ROESY correlations are given from rosaroxine A (**1**) and rosaroxine C (**3**).

Compound **1** was obtained as a colorless solid. Its molecular formula C_30_H_48_O_6_ with ten degrees of unsaturation from HRESIMS *m*/*z* = 503.3376 [M–H]^−^ possibly belongs to triterpenoids. The ^1^H NMR spectroscopic data (Table 1) of **1** showed the occurrence of an olefinic proton (δ_H_ 5.29), two hydroxymethine protons (δ_H_ 3.92, 3.31), one hydroxymethylene proton (δ_H_ 3.76, 3.70), and six methyl groups (δ_H_ 1.30, 1.29, 0.97, 0.86, 0.79, 0.98) (Table 1), similar to the characteristics of euscaphic acid,^9^ besides a hydroxymethylene group and the absence of the doublet methyl signal in compound **1**. The ^13^C and DEPT NMR data (Table 1) displayed 30 carbon resonances, consisting of nine methylenes (δ_C_ 65.0, 42.4, 38.7, 34.1, 29.5, 26.7, 24.7, 22.2, 19.2), seven methines (δ_C_ 129.7, 80.1, 67.1, 55.0, 49.7, 49.6, 48.1), eight quaternary carbons (δ_C_ 182.3, 139.5, 74.2, 48.9, 42.7, 41.1, 39.4, 39.3), and six methyl groups (δ_C_ 29.2, 27.2, 24.5, 22.4, 17.5, 16.8). These data suggest that compound **1** belongs to the group of ursane-type triterpenoids. Compared to the ^13^C NMR spectrum of euscaphic acid,^9^ the presumption that an oxygenated methylene signal in compound **1** replaced the doublet methyl (C-30) in euscaphic acid was supported. The cross-peaks visible in the HMBC spectrum from the methylene protons H-30 (δ_H_ 3.70 and 3.76) to δ_C_ 74.1 (s, C-19), 49.7 (d, C-20), and 22.2 (t, C-21) confirmed the presence of a hydroxymethylene group at C-30. The HMBC correlations from oxygenated H-3 (δ_H_ 3.31) to C-1 (δ_C_ 42.4), C-5 (δ_C_ 49.6), C-2 (δ_C_ 67.1), C-23 (δ_C_ 29.2), and C-24 (δ_C_ 22.4), and from H-2 (δ_H_ 3.92) to C-1 and C-3 established a 2,3-bishydroxyl substitution pattern (Figure 2). The double bond C-12/13 was confirmed by the HMBC correlations between the olefinic proton (δ_H_ 5.29) with C-18 (δ_C_ 55.0, d), C-9 (δ_C_ 48.1, d), and C-14 (δ_C_ 42.7). Finally, the carbonyl group was located at C-28 by the HMBC correlations from H-18, H-22 (δ_H_ 1.64), and H-16 (δ_H_ 2.63) and the carbonyl signal (δ_C_ 182.3). On the basis of these data, the structure of **1** was established as shown in Figure 1. The key HMBC and ^1^H–^1^H COSY correlations of **1** and **3**–**4** and ROESY correlations of **1** and **3** are given in Figure 2.

**Table 1:** ^1^H (600 MHz) and ^13^C (150 MHz) NMR Data of the Triterpenoids rosaroxine A–D (**1**–**4**) (Recorded in CD_3_OD; δ in ppm, *J* in Hz)

position	δ_H_ (**1**)	δ_C_ (**1**)	δ_H_ (**2**)	δ_C_ (**2**)	δ_H_ (**3**)	δ_C_ (**3**)	δ_H_ (**4**)	δ_C_ (**4**)
1	1.27 (dd, 12.3, 11.9)	42.4, CH_2_	1.28 (dd, 12.3, 11.9)	42.4, CH_2_	2.25 (d, 12.1)	54.3, CH_2_	4.10 (s)	85.5, CH
	1.56 (dd, 12.3, 4.4)		1.56 (dd, 12.3, 4.4)		2.31 (d, 12.1)			
2	3.92 (ddd, 11.9, 4.4, 2.8)	67.1, CH	3.92 (ddd, 11.9, 4.4, 3.0)	67.1, CH_2_		212.6, C		212.2, C
3	3.31 (d, 2.8)	80.1, CH	3.32 (d, 3.0)	80.1, CH	3.99 (s)	83.9, CH	4.03 (s)	82.2, CH
4		39.4, C		39.4, C		46.5, C		46.3, C
5	1.30 (m)	49.6, CH	1.27 (m)	49.3, CH	1.56 (m)	55.7, CH	1.46 (d, 9.6)	52.6, CH
6	1.38 (td, 11.8, 2.9)	19.2, CH_2_	1.40 (dd, 12.3, 2.9)	19.2, CH_2_	1.50 (m)	19.8, CH_2_	1.46 (dd, 12.3, 2.9)	19.3, CH_2_
	1.44 (m)		1.46 (m)		1.68 (m)		1.70 (m)	
7	1.30 (m)	34.1, CH_2_	1.29 (m)	34.0, CH_2_	1.39 (m)	33.8, CH_2_	1.38 (m)	33.9, CH_2_
	1.58 (m)		1.58 (m)		1.68 (m)		1.66 (m)	
8		41.1, C		41.1, C		41.4, C		41.1, C
9	1.84 (m)	48.1, CH	1.87 (m)	48.1, CH	1.84 (m)	48.1, CH	1.87 (m)	48.1, CH
10		39.3, C		39.3, C		44.5, C		50.1, C
11	2.01 (m)	24.7, CH_2_	2.01 (m)	24.6, CH_2_	1.95 (m)	24.5, CH_2_	2.10 (m)	27.7, CH_2_
							2.51 (dt, 19.5, 5.4)	
12	5.29 (t, 3.6)	129.7, CH	5.31 (t, 3.6)	129.5, CH	5.32 (t, 3.7)	129.1, CH	5.30 (t, 3.1)	130.4, CH
13		139.5, C		139.2, C		139.2, C		138.1, C
14		42.7, C		42.7, C		42.8, C		42.6, C
15	1.00 (m)	29.5, CH_2_	1.00 (m)	29.5, CH_2_	1.05 (m)	29.5, CH_2_	1.02 (m)	29.6, CH_2_
	1.81 (m)		1.80 (m)		1.82 (m)		1.77 (m)	
16	1.52 (m)	26.7, CH_2_	1.53 (m)	26.6, CH_2_	1.55 (m)	26.6, CH_2_	1.54 (m)	26.6, CH_2_
	2.63 (td, 13.3, 4.5)		2.75 (td, 13.2, 4.5)		2.73 (td, 13.2, 4.6)		2.75 (td, 13.2, 4.4)	
17		48.9, C		48.7, C		48.8, C		48.8, C
18	2.49 (s)	55.0, CH	2.48 (s)	53.8, CH	2.49 (s)	53.8, CH	2.47 (s)	53.7, CH
19		74.1, C		71.9, C		71.9, C		71.8, C
20	1.32 (m)	49.7, CH	2.24 (dd, 12.6, 2.6)	55.1, CH	2.28 (m)	54.4, CH	2.23 (m)	55.2, CH
21	1.51 (m)	22.2, CH_2_	1.59 (m)	23.2, CH_2_	1.61 (m)	23.0, CH_2_	1.60 (m)	23.3, CH_2_
	1.91 (m)		2.14 (m)		2.14 (m)		2.16 (m)	
22	1.64 (dd, 13.1, 4.1)	38.7, CH_2_	1.65 (dd, 13.1, 4.3)	38.3, CH_2_	1.64 (m)	38.0, CH_2_	1.63 (m)	38.2, CH_2_
	1.82 (m)		1.82 (m)		1.82 (m)		1.80 (m)	
23	0.97 (s)	29.2, CH_3_	0.98 (s)	29.2, CH_3_	1.17 (s)	29.6, CH_3_	1.16 (s)	29.3, CH_3_
24	0.86 (_S_)	22.4, CH_3_	0.86 (s)	22.4, CH_3_	0.71 (_S_)	17.1, CH_3_	0.68 (s)	16.8, CH_3_
25	0.79 (s)	17.5, CH_3_	0.78 (s)	17.5, CH_3_	0.88 (s)	16.6, CH_3_	0.79 (s)	12.4, CH_3_
26	0.98 (s)	16.8, CH_3_	0.98 (s)	16.8, CH_3_	0.79 (s)	17.0, CH_3_	0.78 (s)	17.3, CH_3_
27	1.30 (s)	24.5, CH_3_	1.36 (s)	24.5, CH_3_	1.39 (s)	24.4, CH_3_	1.41 (s)	24.4, CH_3_
28		182.3, C		181.7, C		181.4, C		181.7, C
29	1.29 (s)	27.2, CH_3_	1.23 (s)	28.5, CH_3_	1.23 (s)	28.4, CH_3_	1.23 (s)	28.5, CH_3_
30	3.70 (dd, 10.9, 5.9)	65.0, CH_2_		181.7, C		181.1, C		182.0, C
	3.76 (dd, 10.9, 3.5)							

The relative configuration of **1** was determined by the ROESY spectrum. The ROESY correlations of H-3 to H-2 (δ_H_ 3.92) and H-24 (δ_H_ 0.86), of H-26 (δ_H_ 0.98) to H-3 (δ_H_ 3.31), of H-18 (δ_H_ 2.49) to H-29 (δ_H_ 1.29), and of H-30 to H-27 suggested their orientations as shown in Figure 2b. Compound **1** was named rosaroxine A.

Compound **2** was obtained as a colorless solid. Its molecular formula was determined as C_30_H_46_O_7_ from HRESIMS *m*/*z* = 517.3176 [M–H]^−^ as well as the NMR spectra, showing an additional degree of unsaturation in comparison to **1**. The ^1^H NMR spectra (Table 1) revealed the similarity to euscaphic acid,^9^ due to the absence of the doublet methyl signal. Its ^13^C NMR as well as HMBC spectra indicated two carbonyl carbons resonating at δ_C_ 181.7 and the absence of the methyl group C-30. The HMBC cross-peaks from H-20 (δ_H_ 1.59) and H-21 (δ_H_ 2.24) to carbonyl supported a new carbonyl of C-30. The assignations of the other signals were performed by using the correlations visible in the HMBC and HSQC spectra. Thus, **2** was subsequently named rosaroxine B.

Compound **3** was obtained as a white, amorphous powder. Its molecular formula was determined as C_30_H_44_O_7_ from HRESIMS and NMR data. The ^1^H and ^13^C NMR spectra of **3** were similar to those of 2-oxo-pomolic acid (**5**).^10^ However, the signals of the doublet CH_3_-30 in compound **5** were absent in compound **3**, and instead, a carbonyl (δ_C_ 181.1, s) was observable in the ^1^H and ^13^C NMR spectra of **3**. The HMBC correlation from H-20 (δ_H_ = 2.28) to the carbonyl function supported this. The ROESY correlations of H-3 with H-5 (δ_H_ 1.56) and H-23 (δ_H_ 1.17) suggest that H-3 was α-oriented. Additionally, the chemical shifts of H-3 (δ_H_ 3.99) and C-4 (δ_C_ 46.5) were consistent with previously reported data (δ_H_ 3.99 and δ_C_ 45.3 at α-orientation, 3.50 and δ_C_ 42.5 at β-orientation).^11^ On the basis of these data, the chemical structure of **3** was established as shown in Figure 1.

Compound **4** was obtained as a white, amorphous powder. The ^1^H and ^13^C NMR spectra of compound **4** were almost identical with those of **3**, except the methylene signal (δ_C_ 54.3) present in compound **3**, which was substituted by a signal of a hydroxymethine (δ_C_ 85.5) in compound **4**. Its molecular formula was determined as C_30_H_44_O_8_ deduced from the HRESIMS data in combination with the NMR data, indicating one OH group more than in compound **3**. The HMBC correlations from H-1 (δ_H_ 4.10) to C-25 (δ_C_ 12.4), C-2 (δ_C_ 212.2), and C-10 (δ_C_ 50.1) confirmed this OH group at position C-1. The configuration of this OH group should be β-orientated, established by the ROESY correlation of H-1 with H-5 as that of **3**. Compounds **3** and **4** were subsequently named as rosaroxine C and rosaroxine D. The NMR and MS spectra of compounds **1**–**4** are given in the Supporting Information (Figures S1–S4).

The other isolated compounds were identified as 2-oxo-pomolic acid (**5**),^10^ 3-*O*-acetyl-pomolic acid (**6**),^12^ euscaphic acid (**7**),^9^ β-hydroxy-euscaphic acid (**8**),^13^ 2α,3α,19,24-tetrahydoxy-18,19-secours-11,13(18)-dien-28-oic acid (**9**),^14^ kajiichigoside F1 (**10**),^15^ 2-oxo-benthamic acid 28-*O*-β-d-*O*-glucopyranosyl ester (**11**),^16^ 2α,3β,19β-trihydroxy-12-en-ursonic acid-28-*O*-β-d-glucopyranosyl ester (**12**),^17^ 2α,3α,19α-trihydroxy-12-en-oleanolic acid-28-*O*-β-d-glucopyranosyl ester (**13**),^20^ (+)-1″-methylene-6″-hydroxy-2*H*-furan-5″-one-6-catechin (**14**),^18^ catechin (**15**),^19^ quercetin (**16**),^19^ tiliroside (**17**),^20^ 1-[(*E*)-3-(4-hydroxy-phenyl)-2-propenoate β-d-glucopyranosyl ester] (**18**),^21^ 1-feruloyl-β-d-glucopyranosyl ester (**19**),^22^ methyl (*E*)-*p*-coumarate (**20**),^23^ gallic acid (**21**),^24^ protocatechuic acid (**22**),^25^ 5-hydroxymaltol (**23**),^26^ furan-2-carboxylic acid (**24**),^27^ and 2-hydroxy-butanedioic acid-1-methyl ester (**25**).^28^ The NMR spectra of **5**–**10** and the data of compounds **11**–**25** are provided in the Supporting Information (Figures S5–S25).

### 2.2 Quantification of the Most Active Compounds

The quantitative assessment of the active compounds **3**, **5**, **15**, **16**, **21**, and **22** by LC-MS in the fruits of *R. roxburghii* revealed contents of about 3.08, 17.40, 76.29, 1.82, 1.70, and 5.58 mg kg^–1^ dried fruits (Table 2). The obtained analytical data are given in Supporting Information (S27). This high content of antioxidants may contribute to the health benefit after uptake.^29,30^

**Table 2:** Concentrations of Isolated Bioactive Compounds in Fruits of *R. roxburghii*

compound	standards (mg mL^–1^)	sample conc (ng mL^–1^)	content in total extract (mg kg^–1^)	content in fruits (mg kg^–1^)
**3**	2.5	153.9	61.56	3.08
**5**	2.5	869.9	347.96	17.40
**15**	2.5	3814.5	1525.8	76.29
**16**	10.0	363.9	36.39	1.82
**21**	2.5	85.2	34.08	1.70
**22**	2.5	278.9	111.56	5.58

### 2.3 Bioassays

#### 2.3.1 Radical Scavenging Activities

Bearing in mind that the fruits of *R. roxburghii* are a part of the regular diet by local people, we systematically assessed potential health promoting properties of identified compounds. An initial screening of all identified compounds including the ethyl acetate and water layer indicated potent radical scavenging activities of compounds **3**, **5**, **15**, **16**, **21**, and **22** as well as the ethyl acetate and water phases. These compounds were further selected for testing the anti-inflammatory, hepatoprotective, anti-aging, and antioxidant activities. For the screening and evaluation of antioxidant activity of obtained extracts and pure compounds, the widely used DPPH assay was performed, with ascorbic acid as a positive control. The ethyl acetate and water fractions and the purified compounds **15**, **16**, **21**, and **22** showed powerful free radical scavenging effects in comparison to ascorbic acid (Table 3). The potent radical scavenging activities of the water and ethyl acetate extracts are in line with the data from *R*. *roxburghii*(6) and other *Rose* species, e.g., *R. davurica* Pall,^31^ due to the high concentration of the water-soluble ascorbic acid as well as other antioxidants in the aqueous extracts.^4,32^

**Table 3:** Phenolics Catechin (**15**), Quercetin (**16**), Gallic Acid (**21**), and Protocatechuic Acid (**22**) Exerted Significant Radical Scavenging Activities in Comparison to Ascorbic Acid

samples	DPPH	COX-2	COX-1
**3**	no activity	37.4	40.3
**5^a^**	no activity	16.6	28.2
**15**	13.4	53.3	29.0
**16**	13.1	18.8	33.1
**21**	10.0	26.3	40.0
**22**	15.2	no activity	no activity
Ascorbic acid	31.3	/	/
EtOAc layer^b^	9.3	<25	<25
Water layer^b^	16.9	55.5	31.4
Acetaminphen		45.0	>100

a The triterpenoid 2-oxo-pomolic acid (**5**) exhibited notable anti-inflammatory activities in the COX-2/1 assay. The EC_50_ values are given in μM.

b The EC_50_ values of the EtOAc and H_2_O layer are given in μg mL^–1^.

#### 2.3.2 COX-1/2 Assay

In the COX-1/COX-2 assays, both triterpenoids **3** and **5** exerted significant activities in comparison to the positive control acetaminophen (Table 3). A possible keto/enol tautomeric effect at C-2 may explain this effect even though no aromatic ring system is present in both compounds.

#### 2.3.3 Anti-aging and Hepatoprotective Assays

**Figure 3:**
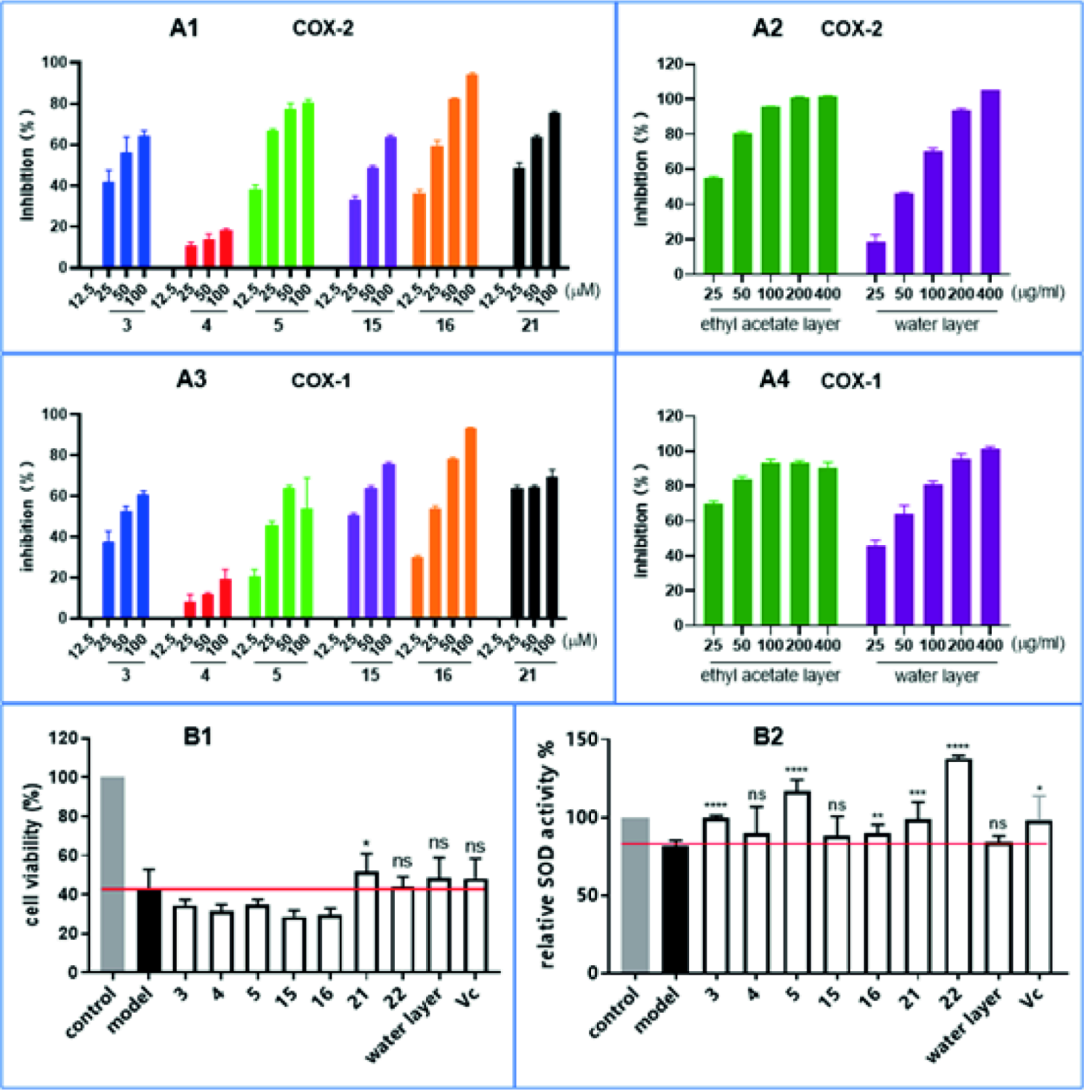
Rosaroxine C (**3**) and 2-oxo-pomolic acid (**5**) and catechin (**15**), quercetin (**16**), and gallic acid (**21**) exhibited high inhibition rates in the COX-2/1 assay (A). Gallic acid (**21**) improved the survival rate of cells in comparison to the model (B1). Rosaroxine C (**3**), 2-oxo-pomolic acid (**5**), and protocatechuic acid (**22**) as the most active compounds in the anti-aging assay after UV–B irradiation (B2). *p < 0.1, **p < 0.01, ***p < 0.001, ****p < 0.0001; ns: not significant compared to the model group.

The anti-aging effects were assessed by using HaCat cells. The results disclosed rosaroxine C (**3**), 2-oxo-pomolic acid (**5**), and protocatechuic acid (**22**) as the most active compounds (Figure 3, B2). According to a previous study^33^ protocatechuic acid (**22**) is regarded as a natural anti-aging agent; therefore, this result is not a surprise. In contrast, the positive effects of the triterpenoids rosaroxine C (**3**) and 2-oxo-pomolic acid (**5**) in this assay were somehow unexpected. A possible keto/enol tautomeric effect at C-2 involving adjacent carbons may explain the observed effects. The selected samples **3**, **5**, **15**, **16**, **21**, **22**, the ethyl acetate, and water phase were also used to test the cell viability of HepG2 cells at the tested concentration of 100 μM. The obtained results exhibited no protective effects in this assay (Figure 3, B1).

## 3 Materials and Methods

### 3.1 General Information

Optical rotations were measured with a RUDOLPH APVI-6 automatic polarimeter. UV spectra were recorded on a Shimadzu 2401A spectrophotometer. CD data were recorded with an Applied Photophysics V100 (Agilent, USA). 1D and 2D NMR spectra were acquired using Bruker AVANCE III-600 MHz spectrometers with SiMe_4_ as an internal standard. MS data were obtained using a Shimadzu UPLC-IT-TOF (Shimadzu Corp., Kyoto, Japan).

Column chromatography (CC) was performed on either silica gel (200 to 300 mesh, Qing-dao Haiyang Chemical Co., Ltd., Qingdao, China) or RP-18 silica gel (20 to 45 μm, Fuji Silysia Chemical Ltd., Japan). Fractions were monitored by TLC on silica gel plates (GF254, Qingdao Haiyang Chemical Co., Ltd., Qingdao, China), and spots were visualized with 10% sulfuric acid in ethanol. MPLC was performed using a Buchi pump system coupled with RP-18 silica gel-packed glass columns (19 × 480, 40 × 480, 45 × 480, and 55 × 480 mm, respectively).

HPLC was performed using Waters 1525EF pumps coupled with analytical semipreparative or preparative Xbridge C_18_ columns (4.6 × 150 and 19 × 250 mm). The HPLC system consisted of a Waters 2998 photodiode array detector and Waters fraction collector III. 1,1-Diphenylpicrylhydrazyl (DPPH) was purchased from Sigma Co. For the UPLC-MS analysis, an electrospray ion source (ESI) was used, with a nebulizer temperature of 300 °C, gas flow rate of 8 L min^–1^, spray pressure of 30 psi, collision voltage of 135 V, capillary voltage of 3500 V, cone hole voltage of 65 V, and mass range of 50–1700 *m*/*z*. An Agilent Zorbax SB C18 (150 × 4.6 mm, 5 μm grain size) column was used with a detection wavelength of 210–300 nm and a flow rate of 1.0 mL min^–1^. The injection volume was 10 μL, and the column temperature was 30 °C. MeOH is used as the mobile phase A, and water is used as the mobile phase B. The gradient elution program was as follows: 0–5 min, 5% A; 5–25 min, 5%–95% A; 25–30 min, 95% A; 30–35 min, 100% A.

For quantification, the following parameters were used: Agilent Zorbax SB C18 column (4.6 × 150 mm, 5 μm, grain size); column temperature: 30 °C; flow rate: 1.0 mL min^–1^, injection volume: 10 μL. Gradient of elution: 0–25 min 5% methanol, 95% water, isocratic elution. Mass spectrum parameters: Dual ESI source; atomization pressure of 30 psi; drying gas flow rate: 8 L min^–1^; drying temperature: 350 °C; ion source pressure: 3500 V; capillary pressure: 175 V; taper hole voltage: 65 V; level 4 pole voltage: 750 V; acquisition mode: Auto MS/MS; MS full scan range *m*/*z*: 100–1700; MS/MS full scan range *m*/*z*: 50–1700; acquisition frequency: 1 spectra s^–1^; data storage mode: Centroid.

### 3.2 Plant Material

Mature fruits of *Rosa roxburghii* were collected in October 2021 in Panzhou, Guizhou Province, People’s Republic of China, and identified by Dr. Jie Cai. The voucher specimen (Cai20211002) was preserved in the State Key Laboratory of Phytochemistry and Plant Resources in West China, Kunming Institute of Botany, Chinese Academy of Sciences.

### 3.3 Extraction and Isolation

Twenty kilogram of fresh fruits of *Rosa roxburghii* were crushed and extracted with 200 L of MeOH at room temperature and filtered, and the solvent was evaporated *in vacuo*. The concentrated extract (1 kg) was partitioned between water and ethyl acetate (EtOAc), and the organic solvent was removed under reduced pressure. The mother liquor (100 g) was further subjected to column chromatography (CC) over silica gel (1 kg) eluted with mixtures of CHCl_3_–MeOH (1:0 to 0:1, v/v) to give nine fractions (I–IX). Fraction I (8 g) was subjected to C_18_ MPLC eluted with 60%, 70%, 80%, 90%, and 100% aqueous MeOH. This separation step yielded subfractions I-1–5. Compound **5** (330 mg) crystallized from subfraction I-1. Subfraction I-2 containing impure **6** was chromatographed over prep HPLC using 70–85% aqueous CH_3_CN as eluents. This yielded 10.4 mg of **6**. Subfraction I-3 was also separated by prep HPLC eluted with 25–35% aqueous CH_3_CN to give 5.8 mg of **17**. Subfraction I-4 was also separated by prep HPLC eluted with 30–40% aqueous CH_3_CN to yield 4.7 mg of **13**. Compound **12** (5.0 mg) was crystallized from subfraction I-5. The mother liquid of subfraction I-5 was further subjected to prep HPLC using 30–40% aqueous CH_3_CN to yield 3.6 mg of **9**. Fraction III (15 g) was subjected to C_18_ MPLC eluted with 70–100% methanol–water deionization mixtures followed by 1% acidic methanol to give the 3 subfractions III-1–3. Compound **8** crystallized in an impure form from subfraction III-3. The crystals were dissolved and subsequently purified by prep HPLC with 50–65% aqueous CH_3_CN. This yielded 12.8 mg of **8** and 9.5 mg of **7**. Fraction IV (6 g) was subjected to C_18_ MPLC eluted with mixtures consisting of 40%, 50%, 60%, 70%, 80%, and 90% aqueous methanol followed by 100% methanol to yield seven subfractions (subfractions IV-1–7). Compounds **16** (36 mg) and **15** (1.5 g) crystallized from subfractions IV-2 and subfraction IV-4. Subfraction IV-3 was separated by prep HPLC with 40–55% aqueous CH_3_CN. This step yielded 15.7 mg of **1**. Subfraction IV-4 was further purified with 30–45% aqueous CH_3_CN to afford 8.1 mg of **2** and **4** (6.4 mg). Fraction V (4 g) was subjected to C_18_ MPLC with 20%, 50%, 60%, and 100% aqueous methanol to give subfraction V-1–3. Compound **10** (463 mg) was crystallized from subfraction V-3. Fraction VI (6 g) was subjected to C_18_ MPLC with 30% aqueous methanol to yield subfractions VI-1, VI-2, and VI-3. Subfraction VI-1 was subjected to size exclusion chromatography over Sephadex LH-20 eluted with 30% aqueous methanol, followed by prep HPLC eluted with 5–20% aqueous CH_3_CN to give 2.1 mg of **24**. Fraction VI-2 was also chromatographed over Sephadex LH-60 eluted with MeOH to yield a further 3 subfractions (VI-1–1–VI-1–3). Subfraction VI-1–1 was subjected to prep HPLC using 5–20% aqueous CH_3_CN as eluents to give **18** (5.5 mg). Compound **21** (34.0 mg) was also obtained by separating subfraction VI-1–3 prep HPLC using 5–20% aqueous CH_3_CN as the eluents. Fraction VII (7 g) was subjected to C_18_ MPLC eluted with mixtures of 20%, 40%, and 50% aqueous methanol to give subfractions VII-1–3. Subfraction VII-1 was subjected to Sephadex LH-20 eluted with methanol to give two fractions. One fraction contained a mixture of compounds **23** and **25**, and the other fraction contained impure **22**. Compounds **23** and **25** were separated by prep HPLC eluted with 5–20% aqueous CH_3_CN. This step afforded 1.9 mg of **23** and 15.1 mg of **25**. Compound **22** (110 mg) was also purified in the same manner like compounds **23** and **25**. Fraction VII-2 was subjected to a Sephadex LH-20 gel eluted with methanol to give two subfractions (VII-2–1–VII-2–2). The subfraction VII-2–1 containing a mixture of compounds **14** and **19** was further separated by prep HPLC eluted with 5–20% aqueous CH_3_CN. This yielded 3.9 mg of **14** and 6.6 mg of **19**. Compounds **3** (580 mg) and **11** (11.7 mg) were further purified from subfraction VII-2–2 by prep HPLC eluted with 40–55% aqueous CH_3_CN.

### 3.4 Cyclo-oxygenase (COX-1/2) Inhibition Assay

The inhibition activity of tested compounds against COX-1 and COX-2 was determined by using the COX (ovine) colorimetric inhibitor screening assay kit (Cayman, USA, item number: 760110) according to the manufacturer’s protocol. Each compound was assayed in triplicate, and GraphPad Prism 8 software was used to calculate EC_50_ values. All statistical analyses were performed by Microsoft Excel.

### 3.5 DPPH Assay

The DPPH radical scavenging capacity assay was performed according to a previously described method with slight modifications.^34^ Briefly, the tested compounds or extracts were dissolved in a small volume of DMSO to generate a 10 mM or 100 mg mL^–1^ (for the extracts) stock solution and diluted to generate the final testing solution with methanol. The 0.2 mM fresh DPPH methanol solution was prepared daily. The 90 μL of methanolic solution of DPPH was added to 60 μL of tested compound solution with different concentrations (or 60 μL of methanol as blank) in 96-well plates and allowed to react for 30 min at ambient temperature in the dark. The absorbance (OD) was measured at 517 nm on an Infinite M200 Pro (Tecan, Austria) microplate reader. The level of the DPPH• scavenging activity was calculated as
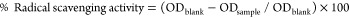
The EC_50_ values were calculated by using GraphPad Prism 8 software 4.6.

### 3.6 Quantification of Compounds **3**, **5**, **15**, **16**, **21**, and **22** by LC-MS

The selected triterpenoids **3** and **5** as well as the phenolics **15**, **16**, **21**, and **22** were quantified by employing the external standard method. The previously purified compounds were dissolved in methanol to reach a concentration of up to 2000 μg mL^–1^. From these stock solutions, dilution series from 2000 to 220 μg mL^–1^ were prepared (three standards per sample; for the concentration of each standard see Supporting Information S27) and measured by LC-MS. The crude extract was diluted to 2.5 mg mL^–1^ for compounds **3**, **5**, **15**, **21**, and **22** and 10 mg mL^–1^ for compound **16**. From the obtained data, the concentration of these compounds in the plant material was calculated.

### 3.7 Anti-aging Bioassay (UV–B Irradiation Model and Viability Assay)

Human HaCaT cells were cultured routinely in DMEM (high glucose) supplemented with 10% fetal bovine serum and 1% penicillin–streptomycin solution in a humidified incubator at 37 °C and 5% CO_2_.^19^ All cells were cultured in culture dishes, and the medium was changed every day. The cells were cultured when the cell density reached 80%. Briefly, the HaCaT cells were cultured for 24 h after plating at a density of 4 × 10^4^ cells/100 μL in 96-well plates. Subsequently, cells were irradiated according to the grouping using a 20 W UV–B lamp (SiTing, Shanghai, CN) at a distance of 10 cm for 5 s. The irradiation dose was calculated to be approximately 0.80 J cm^–2^. Cells were divided into the following groups: control, UV–B alone, and UV–B + samples (25 μM). After UV–B irradiation, HaCaT cells continued to culture for 24 h with the medium containing compounds to be measured. The effects of those compounds on cell protection were determined by the MTS assay (BestBio, Shanghai, CN). After incubation, cells in each well were treated with 20 μL MTS solution for 1 h. The absorbance at 490 nm was measured directly with a microplate reader (FlexStation3, Molecular Devices, CA, US). Cell viability was expressed as the ratio percentage of MTS after deducting background value, assuming that the absorbance of control cells with deducting background absorbance was 100%.

#### 3.7.1 *In Vitro* SOD Activity

The superoxide dismutase type assay kit (Nanjing Jiancheng Bioengineering Institute, Jiangsu, CN) was used to detect whether the compounds in *R. roxburghii* possess a direct promoting effect on the SOD enzymatic activity. Briefly, the cell samples were extracted with lysate buffer after the cells grew to 80% in one cell dish; 25 μM compounds were added to the experimental wells; and the same volume of distilled water was added to the control wells to maintain a constant final volume. Finally, SOD enzyme activity was detected according to the manufacturer’s instructions. Adherent HaCaT cells were incubated in a six-well plate at a density of 1 × 10^6^ cells per well for 24 h. According to the UV–B irradiation model, the cells were treated with UV–B irradiation and different compounds. After 24 h, the total protein was extracted by cell lysis buffer. After the protein concentration of all samples is unified, the enzyme activity is detected according to the instruction of the assay kit.

### 3.8 Hepatoprotective Assay

HepG2 cells were seeded into 96-well tissue culture dishes at 5 × 10^3^ cells/well and cultured overnight in DMEM medium supplemented with 10% FBS and 1% penicillin/streptomycin antibiotics at 37 °C in a humidified 5% CO_2_ incubator. The cells were then incubated with test compounds at 50 μM or extracts at 200 mg mL^–1^ for 4 h before acetaminophen (APAP) exposure. After this pretreatment, the experimental group was treated with 20 mM APAP together with the compounds; the APAP-only group was treated with 20 mM neomycin and an equivalent volume of DMSO; and the control group was treated with an equivalent volume of DMSO without APAP or test compounds. After another 24 h of culture, the cell viability was then measured using the CellTiter 96 Aqueous One Solution Cell Proliferation Assay kit (Promega, USA).^12^

### 3.9 Isolated Compounds

Rosaroxine A (**1**): C_30_H_48_O_6_; [α]_D_^24.8^ +31.2 (c 0.05, CH_3_OH); ^1^H (600 MHz) and ^13^C (150 MHz) NMR data (CD_3_OD) see Table 1; negative HRESIMS *m*/*z* 503.3376 [M–H]^−^ (calcd. for C_30_H_47_O_6_, 503.3378).

Rosaroxine B (**2**): C_30_H_46_O_7_; [α]_D_^25.8^ +4.6 (*c* 0.12, CH_3_OH); ^1^H (600 MHz) and ^13^C (150 MHz) data (CD_3_OD) see Table 1; negative HRESIMS *m*/*z* 517.3176 [M–H]^−^ (calcd. for C_30_H_45_O_7_, 517.3171).

Rosaroxine C (**3**): C_30_H_44_O_7_; [α]_D_^24.7^ +56 (*c* 0.11, CH_3_OH); ^1^H (600 MHz) and ^13^C (150 MHz) data (CD_3_OD) see Table 1; negative HRESIMS *m*/*z* 515.3019 [M–H]^−^ (calcd. for C_30_H_43_O_7_, 515.3014).

Rosaroxine D (**4**): C_30_H_44_O_8_; [α]_D_^25^*+*55 (*c* 0.05, CH_3_OH); ^1^H (600 MHz) and ^13^C (150 MHz) NMR data (CD_3_OD) see Table 1; negative HRESIMS *m*/*z* 531.2965 [M–H]^−^ (calcd. for C_30_H_43_O_8_, 531.2963).

## 4 Conclusions

In this work, we could show that mature rosehips from *R*. *roxburghii* contain a diversity of ursane-type triterpenoids, which may contribute to the taste of these fruits^35^ and phenolics belonging to several subclasses of compounds derived from the shikimic acid pathway. Some of the identified compounds exhibited excellent bioactivities *in vitro*, especially with regard to the radical scavenging activities. Taking the presence of ascorbic acid derivatives as reported recently^4^ into account, these fruits are a rich source of potent antioxidants. The regular uptake of these fruits by the local population in China suggests a positive effect on the health of consumers due to the identified constituents. These results contribute to the phytochemical knowledge of *R. roxburghii* and indicate possible health-promoting properties of these fruits. However, further studies are needed to elaborate these effects.

## Supporting Information Available

Relevant NMR and MS data from all isolated compounds as well as LC-MS from the quantification experiment (PDF)
