# Supplementary material for: Fruits from Rosa roxburghii: A Valuable Bioresource of Potent Radical Scavengers and Novel Ursane-Type Triterpenoids
Source: ACS Omega. 2024 Aug 27;9(36):38023–31. doi: 10.1021/acsomega.4c04893 (PMC11391452; doi:10.1021/acsomega.4c04893)
Supplement: Supplementary file 1 — ao4c04893_si_001.pdf [file ao4c04893_si_001.pdf]

Supplementary Material

Fruits from *Rosa roxburghii*: A valuable bioresource of potent Radical Scavengers and novel ursane-type Triterpenoids

Yang Yu [a,†], Jing Wu [a,†], Mei-Fen Bao [a], Liu Yang[a], Zhi-Lin Cai [b], Johann Schinnerl [c,*] and Xiang-Hai Cai [a,*]

[a] State Key Laboratory of Phytochemistry and Plant Resources in West China, Kunming Institute of Botany, Chinese Academy of Sciences, Kunming 650201, People's Republic of China.

[b] Zhongzhihao Cili Industrial Development (Guizhou) Co., LTD, Panzhou 553599, People's Republic of China.

[c] Department of Botany and Biodiversity Research, University of Vienna, Rennweg 14, A-1030 Vienna, Austria.

[†]Contributed equally to this work

[*]Corresponding Authors:

johann.schinnerl@univie.ac.at (Johann Schinnerl)

xhcai@mail.kib.ac.cn (Xianghai Cai) Tel: +86-871-65223242; Fax: +86-871-65150227

Table of Contents	Page

S1: Spectroscopic analysis of compound **1**..........S3-10
S2: Spectroscopic analysis of compound **2**..........S11-18
S3: Spectroscopic analysis of compound **3**..........S19-26
S4: Spectroscopic analysis of compound **4**..........S27-34
S5: Spectroscopic analysis of compound **5**..........S35-36
S6: Spectroscopic analysis of compound **6**..........S37-38
S7: Spectroscopic analysis of compound **7**..........S39-40
S8: Spectroscopic analysis of compound **8**..........S41-42
S9: Spectroscopic analysis of compound **9**..........S43-44
S10: Spectroscopic analysis of compound **10**..........S45-46
S11: Spectroscopic analysis of compound **11**..........S47-48
S12: Spectroscopic analysis of compound **12**..........S49-50
S13: Spectroscopic analysis of compound **13**..........S51-52
S14: Spectroscopic analysis of compound **14**..........S53-58
S15: Spectroscopic analysis of compound **15**..........S59-60
S16: Spectroscopic analysis of compound **16**..........S61-62
S17: Spectroscopic analysis of compound **17**..........S63-64
S18: Spectroscopic analysis of compound **18**..........S65-66
S19: Spectroscopic analysis of compound **19**..........S67-68
S20: Spectroscopic analysis of compound **20**..........S69-70
S21: Spectroscopic analysis of compound **21**..........S71-72
S22: Spectroscopic analysis of compound **22**..........S73-74
S23: Spectroscopic analysis of compound **23**..........S75-76
S24: Spectroscopic analysis of compound **24**..........S77-78
S25: Spectroscopic analysis of compound **25**..........S79-80
S26: ^{1}H and ^{13}C NMR data of compounds **5**– **25**..........S81-86
S27: Quantification by LC-MS of compounds **3**, **5**, **15**, **16**, **21** and **22**..........from S87

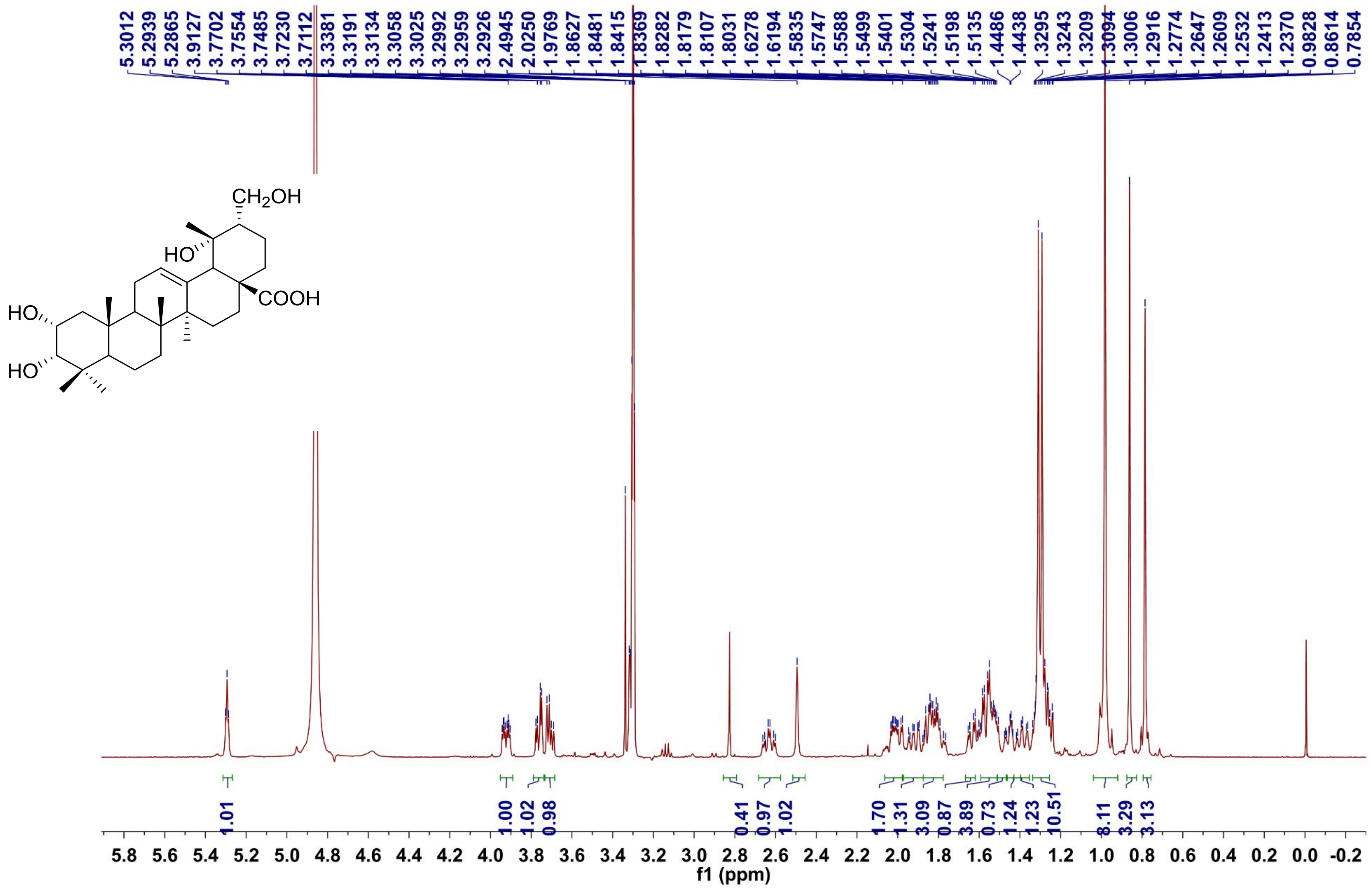

S1-1 ^{1}H NMR spectrum of compound **1** in CD_3OD (500 MHz)

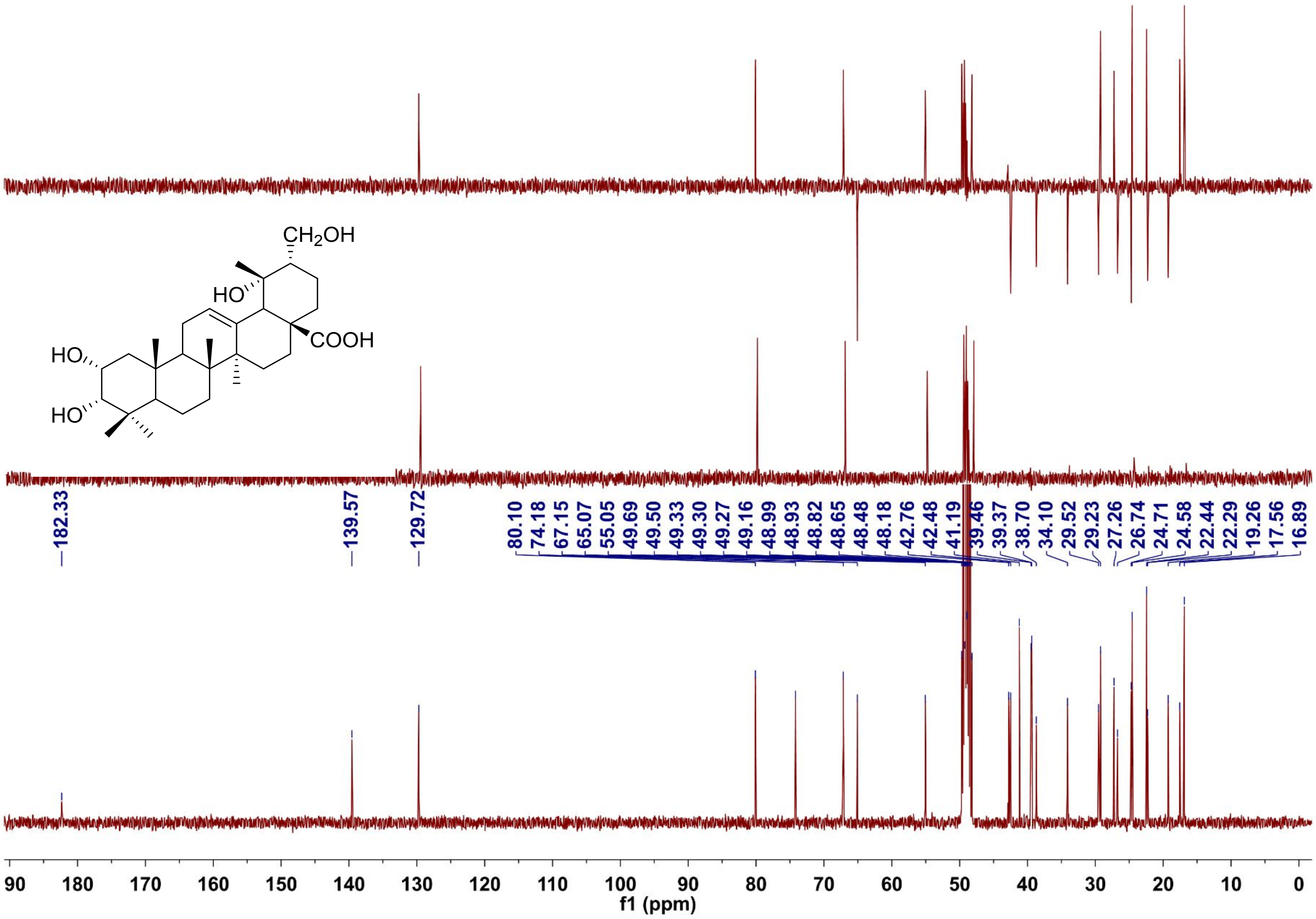

S1-2 ^{13}C NMR spectrum of compound **1** in CD_3OD (125 MHz)

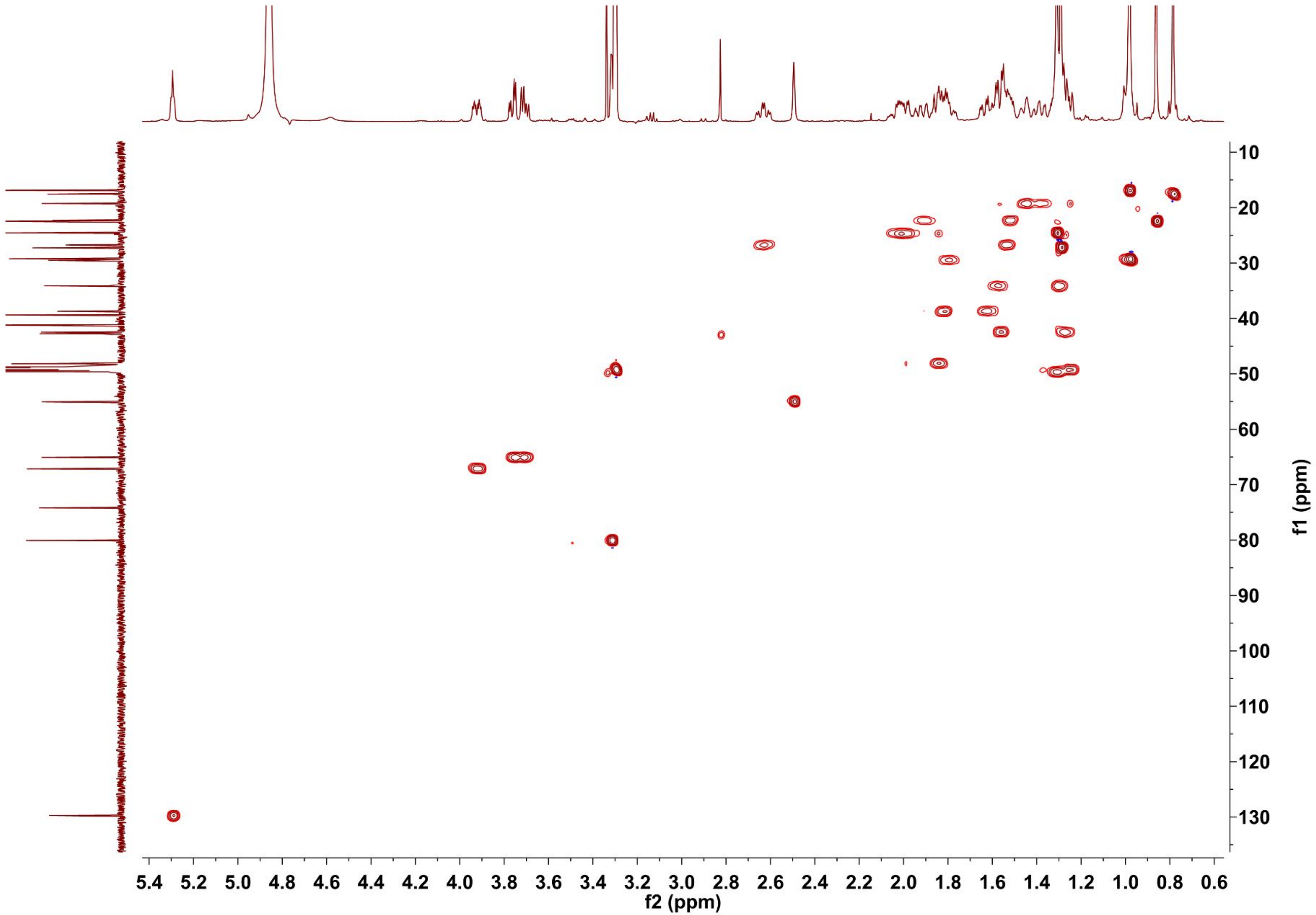

S1-3 HSQC spectrum of compound **1** in CD_3OD (500 MHz)

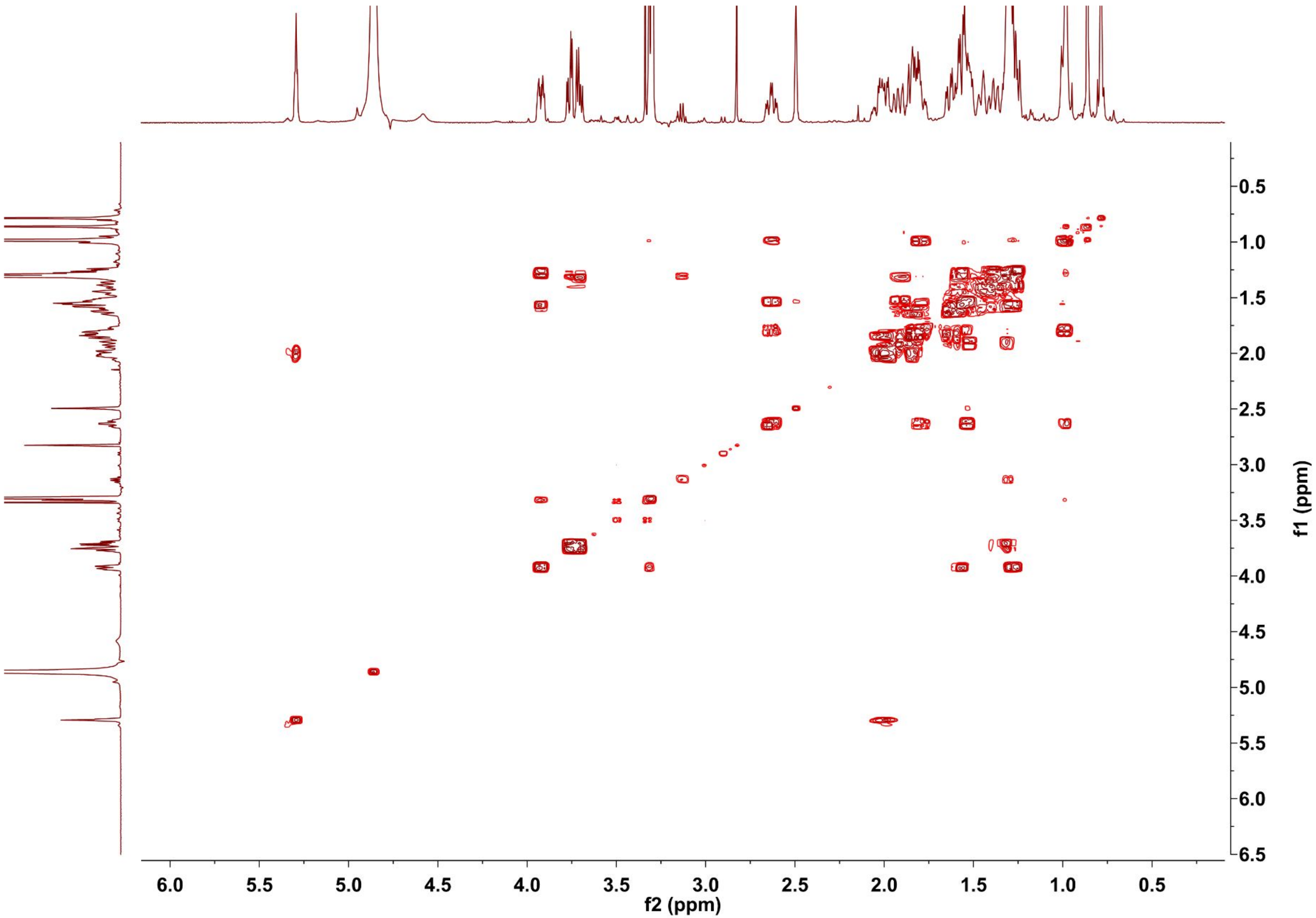

S1-4 ^{1}H-^{1}H COSY spectrum of compound **1** in CD_3OD (500 MHz)

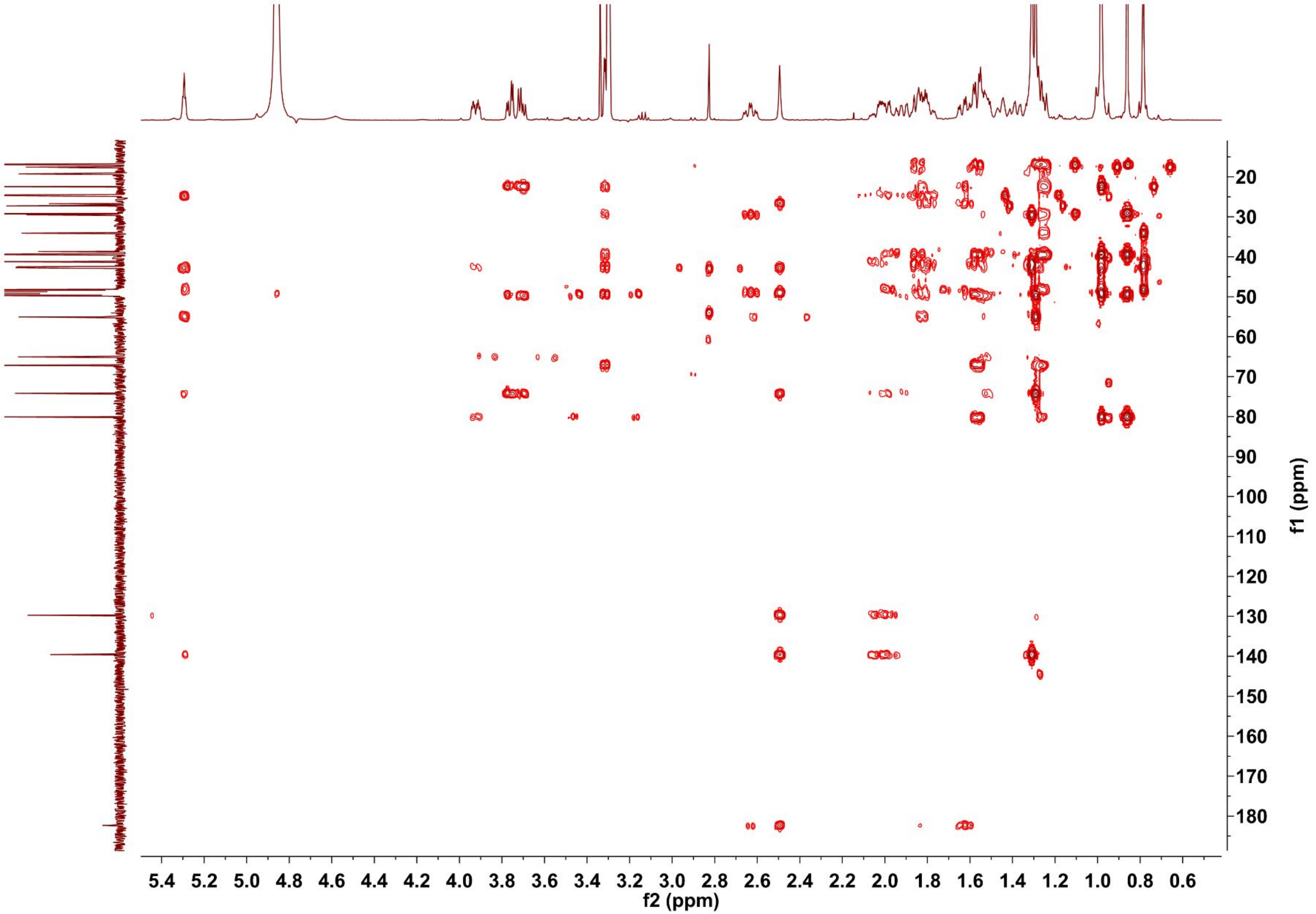

S1-5 HMBC spectrum of compound **1** in CD_3OD (500 MHz)

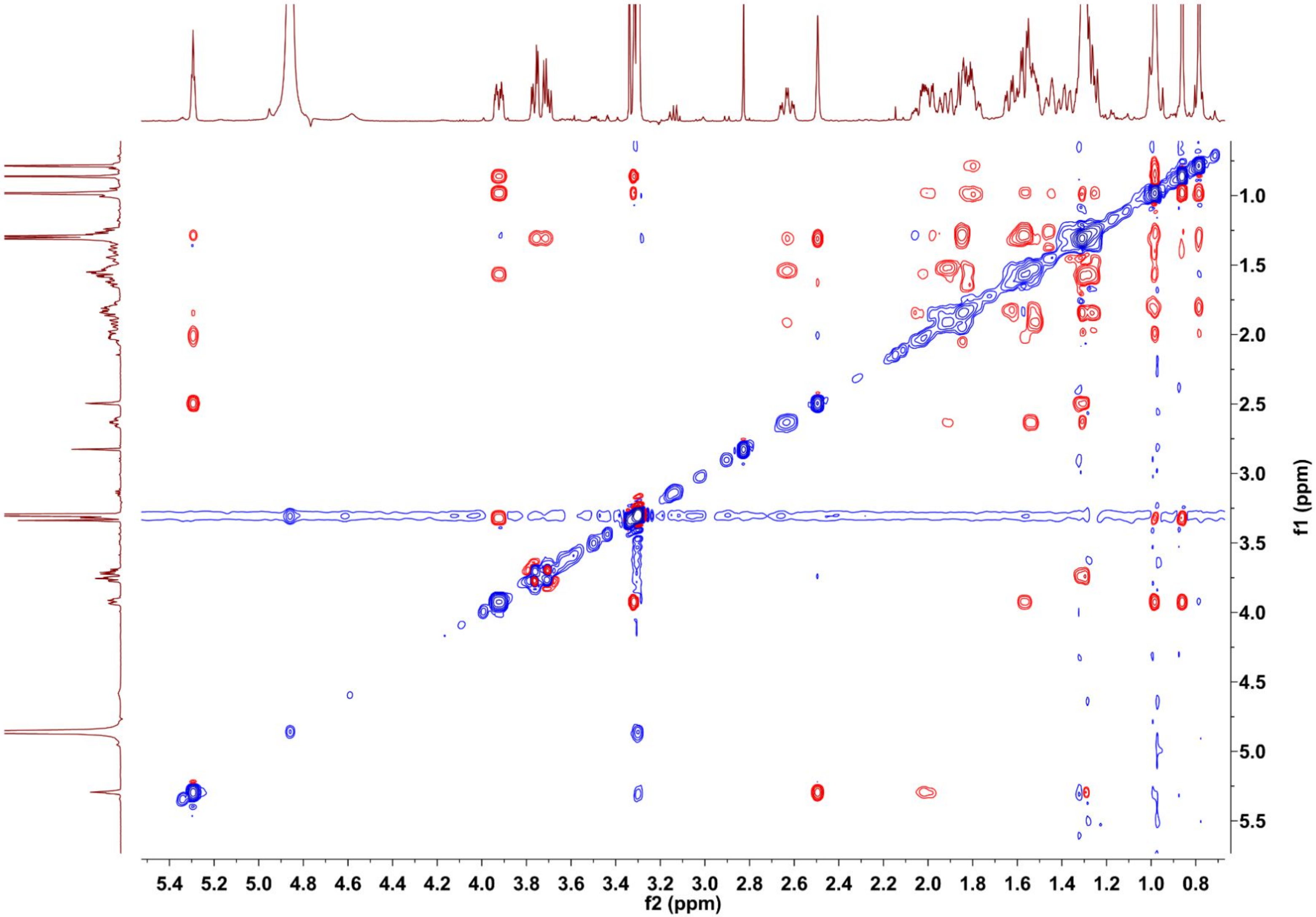

S1-6 ROESY spectrum of compound **1** in CD_3OD (500 MHz)

Data File: E:\DATA\2022\0729\wcl-4a.lcd

Elmt	Val.	Min	Max	Elmt	Val.	Min	Max	Elmt	Val.	Min	Max	Elmt	Val.	Min	Max	Use Adduct
H	1	5	100	F	1	0	0	Cl	1	0	0	Ag	1	0	0	H
2H	1	0	0	Na	1	0	0	Co	2	0	0	I	3	0	0	
B	3	0	0	Mg	2	0	0	Cu	2	0	0	Ir	3	0	0	
C	4	10	60	Si	4	0	0	Se	2	0	0					
N	3	0	10	P	3	0	0	Br	1	0	5					
O	2	0	30	S	2	0	0	Pd	2	0	0					

Error Margin (ppm):	5	DBE Range:	not fixed	Electron Ions:	both
HC Ratio:	unlimited	Apply N Rule:	no	Use MSn Info:	yes
Max Isotopes:	all	Isotope RI (%):	1.00	Isotope Res:	10000
MSn Iso RI (%):	75.00	MSn Logic Mode:	OR	Max Results:	30

Event#: 2 MS(E-) Ret. Time : 0.240 -> 0.360 Scan# : 38 -> 56

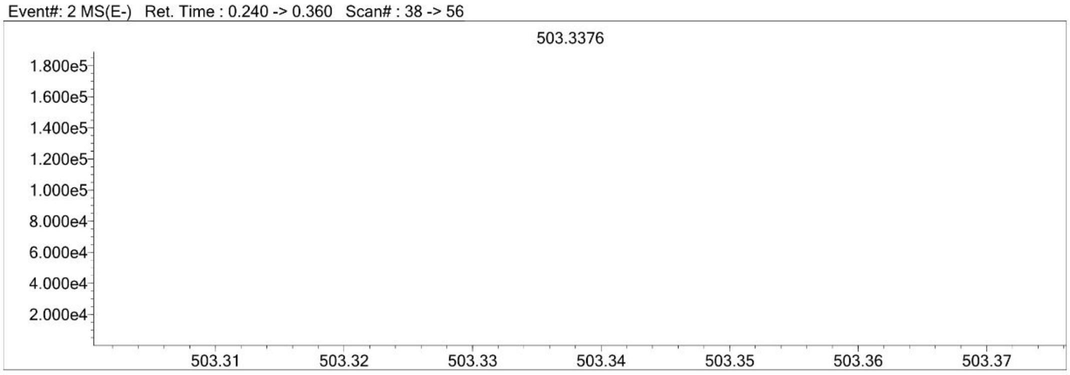

Measured region for 503.3376 m/z

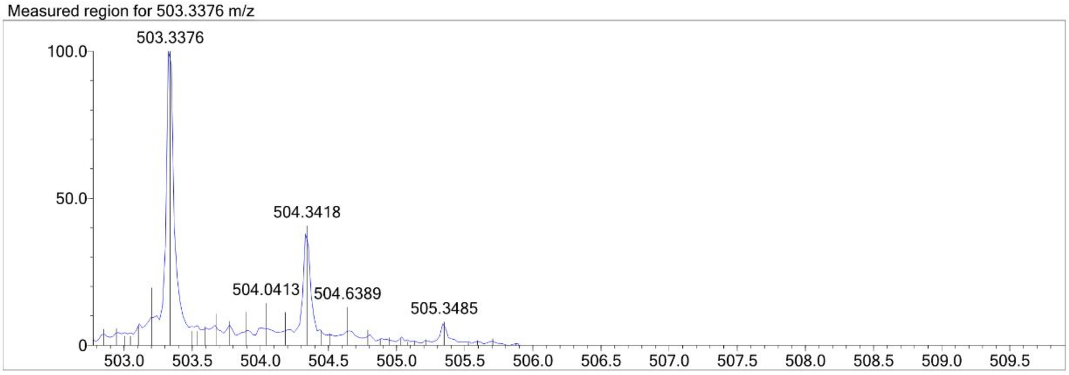

C30 H48 O6 [M-H]- : Predicted region for 503.3378 m/z

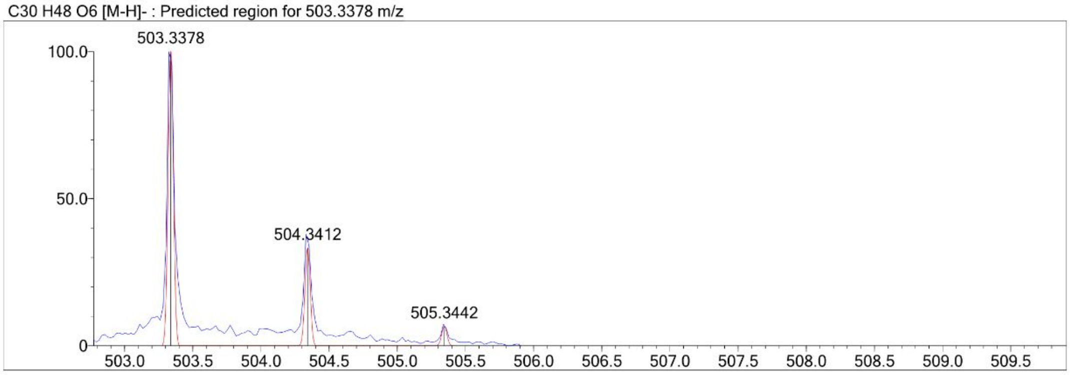

Formula (M)	Ion	Meas. m/z	Pred. m/z	Df. (mDa)	Df. (ppm)	DBE
C30 H48 O6	[M-H]-	503.3376	503.3378	-0.2	-0.40	7.0

S1-7 HRESIMS spectrum of compound **1**

Rudolph Research Analytical

This sample was measured on an Autopol VI, Serial #91058
Manufactured by Rudolph Research Analytical, Hackettstown, NJ, USA.

Measurement Date : Wednesday, 27-JUL-2022

Set Temperature : 25.0

Time Delay : Disabled

Delay between Measurement : Disabled

n	Average	Std.Dev.	% RSD	Maximum	Minimum
5	31.20	1.10	3.52	32.00	30.00

S.No	Sample ID	Time	Result	Scale	OR °Arc	WLG.nm	Lg.mm	Conc.g/100ml	Temp.
1	WCL-4A	07:45:40 PM	32.00	SR	0.016	589	100.00	0.050	24.8
2	WCL-4A	07:45:46 PM	30.00	SR	0.015	589	100.00	0.050	24.8
3	WCL-4A	07:45:53 PM	32.00	SR	0.016	589	100.00	0.050	24.9
4	WCL-4A	07:45:59 PM	32.00	SR	0.016	589	100.00	0.050	24.9
5	WCL-4A	07:46:05 PM	30.00	SR	0.015	589	100.00	0.050	24.9

Page 1 of 1

S1-8 OR spectrum of compound **1** in MeOH

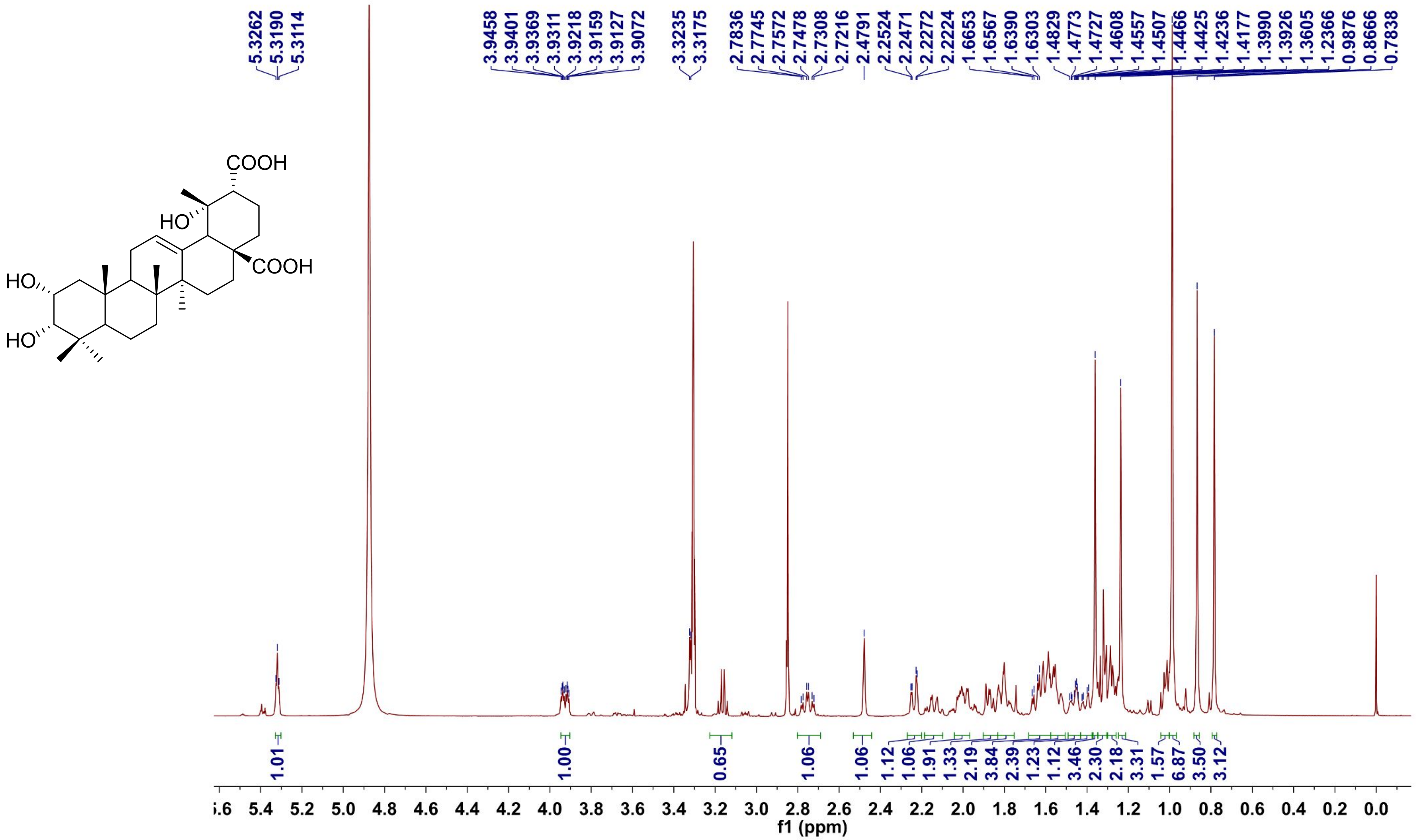

S2-1 [1] H NMR spectrum of compound **2** in CD_3OD (500 MHz)

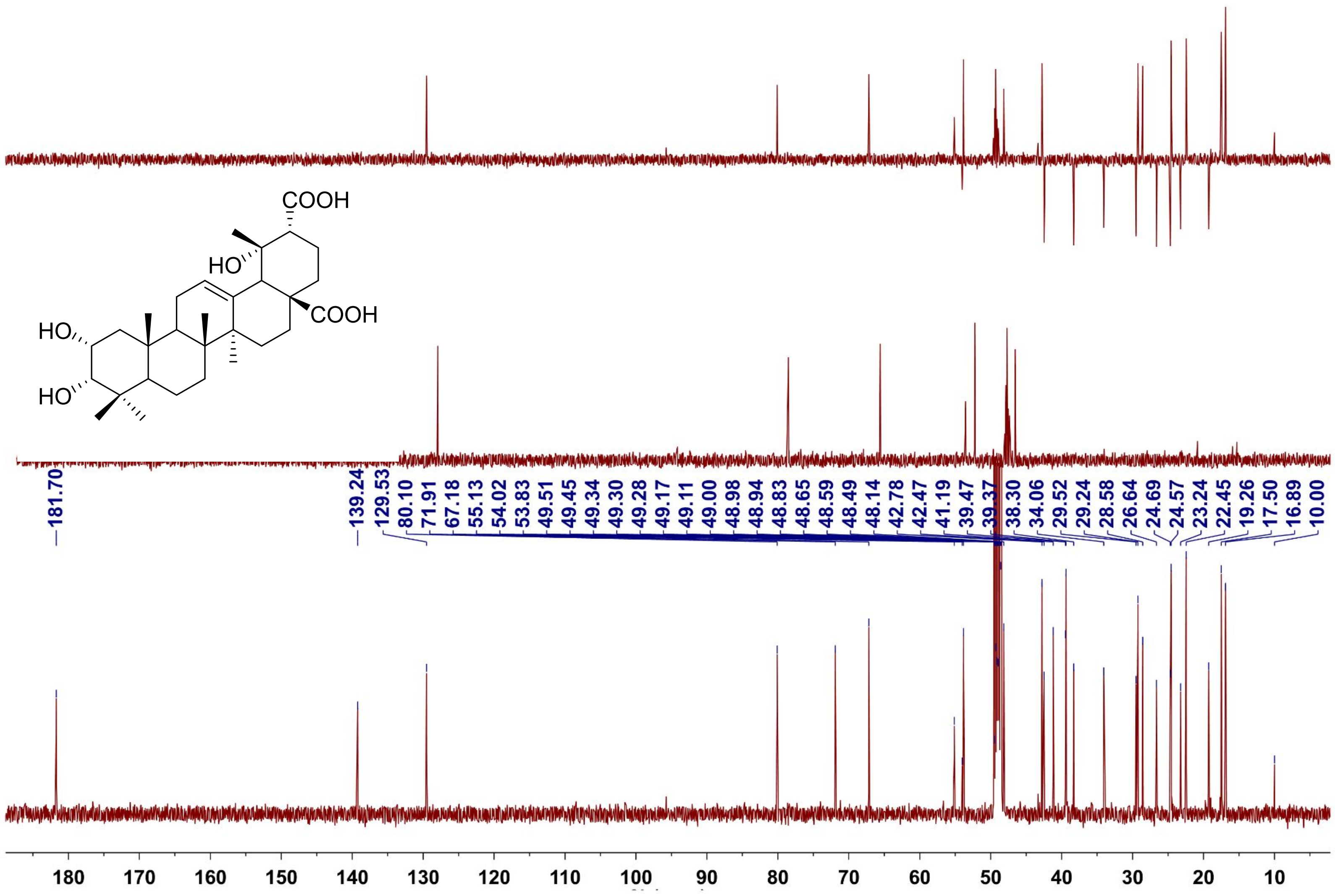

S2-2 ^{13}C NMR spectrum of compound **2** in CD_3OD (125 MHz)

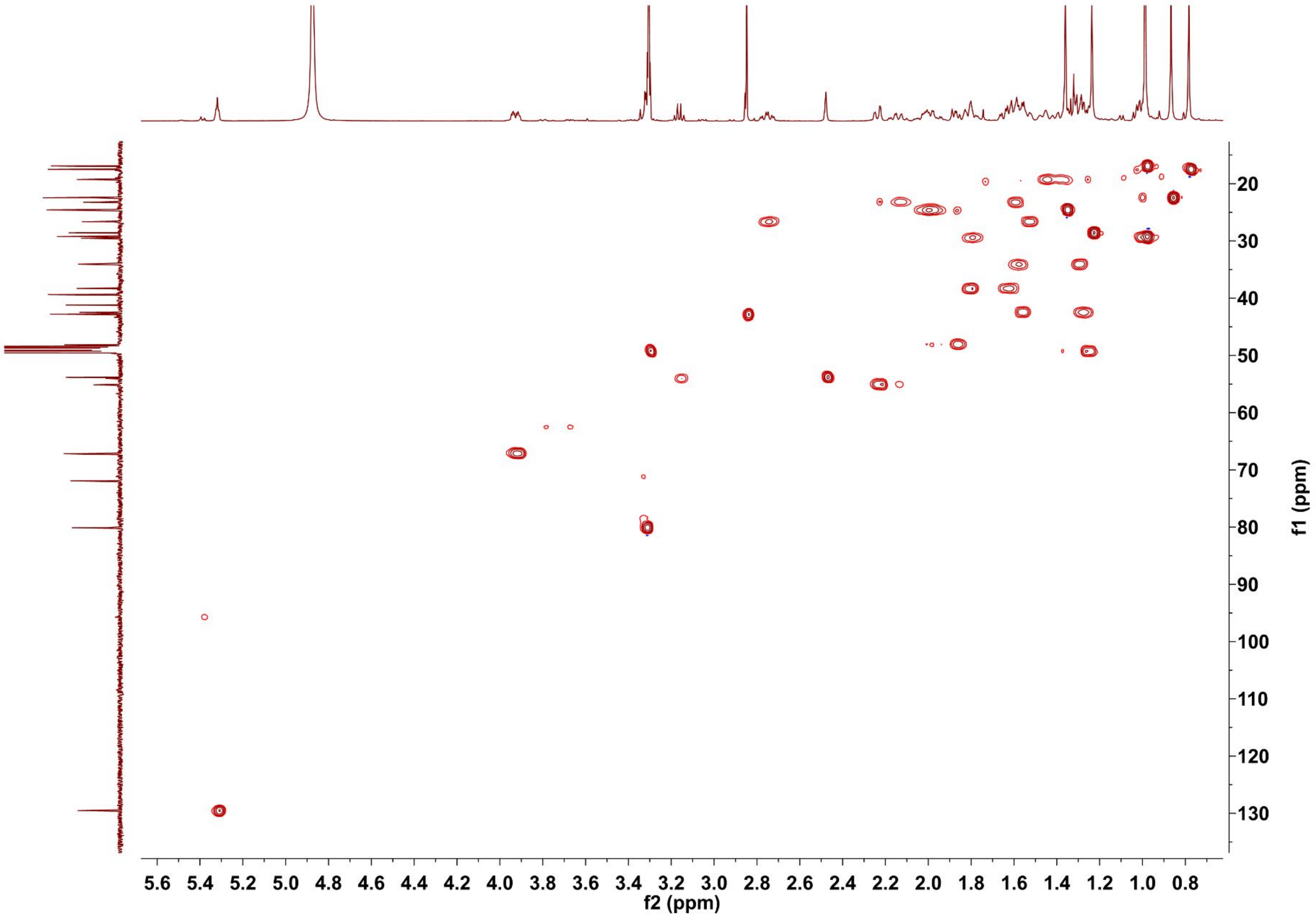

S2-3 HSQC spectrum of compound **2** in CD_3OD (500 MHz)

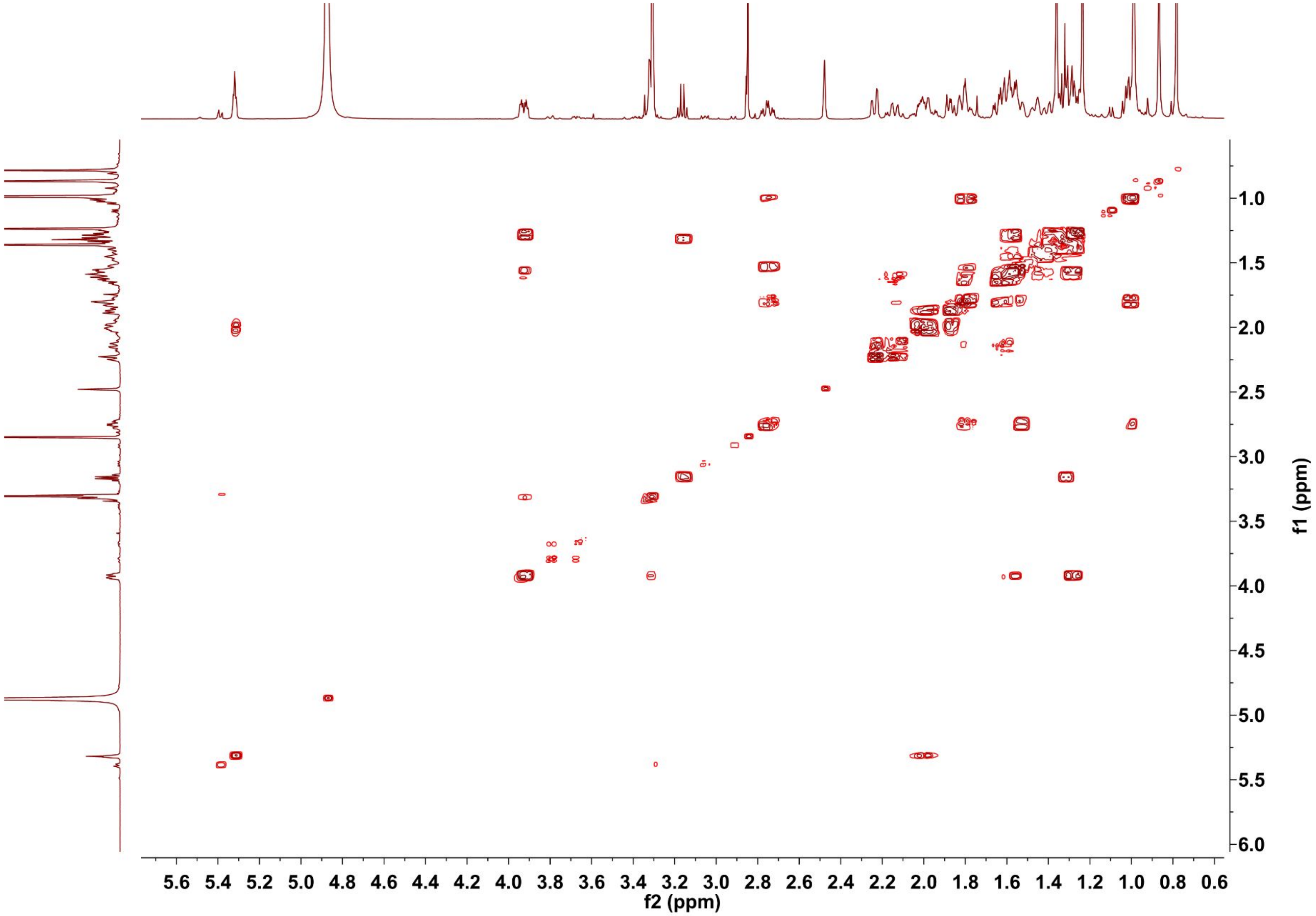

S2-4 ^{1}H-^{1}H COSY spectrum of **2** in CD_3OD (500 MHz)

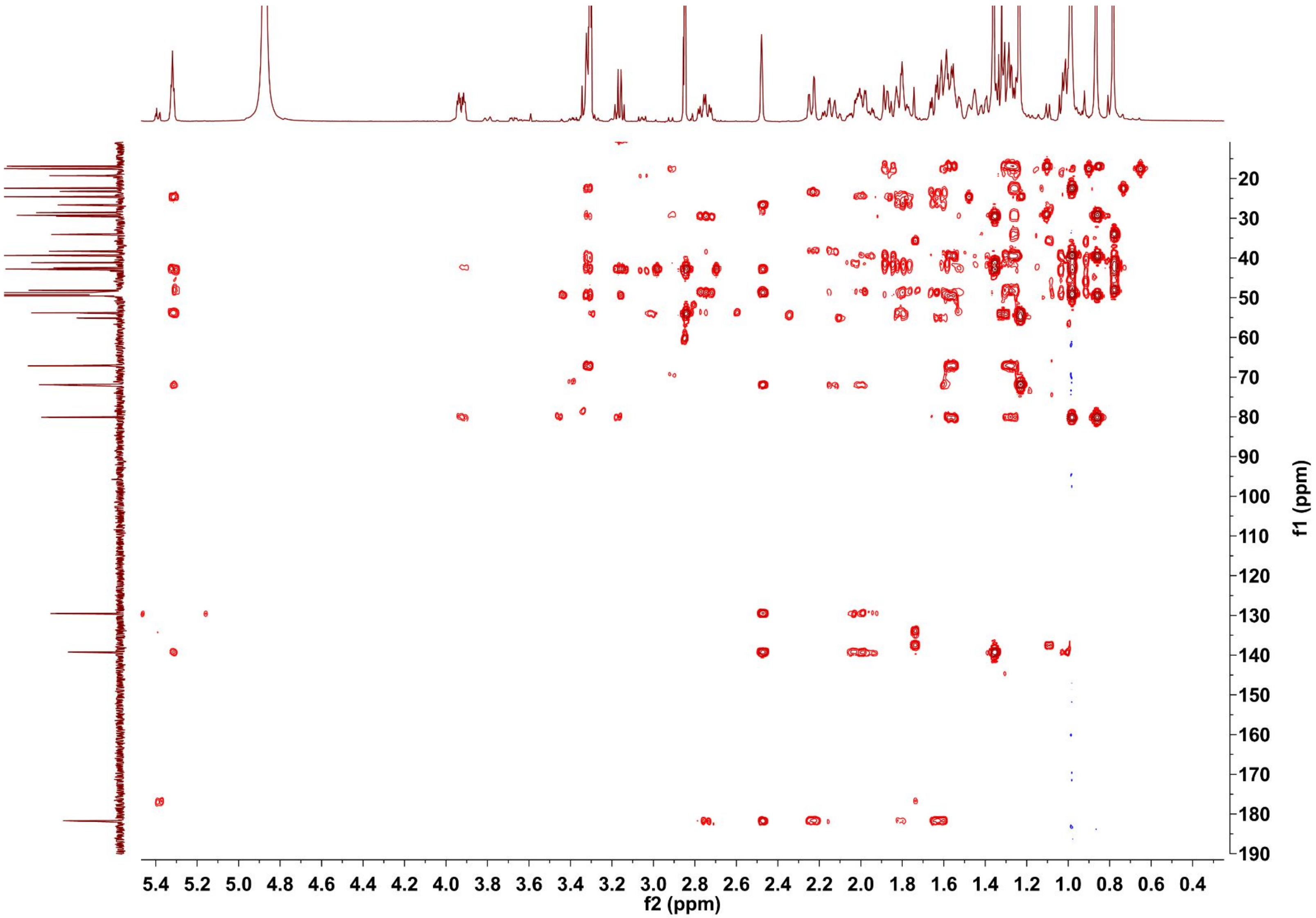

S2-5 HMBC spectrum of **2** in CD_3OD (500 MHz)

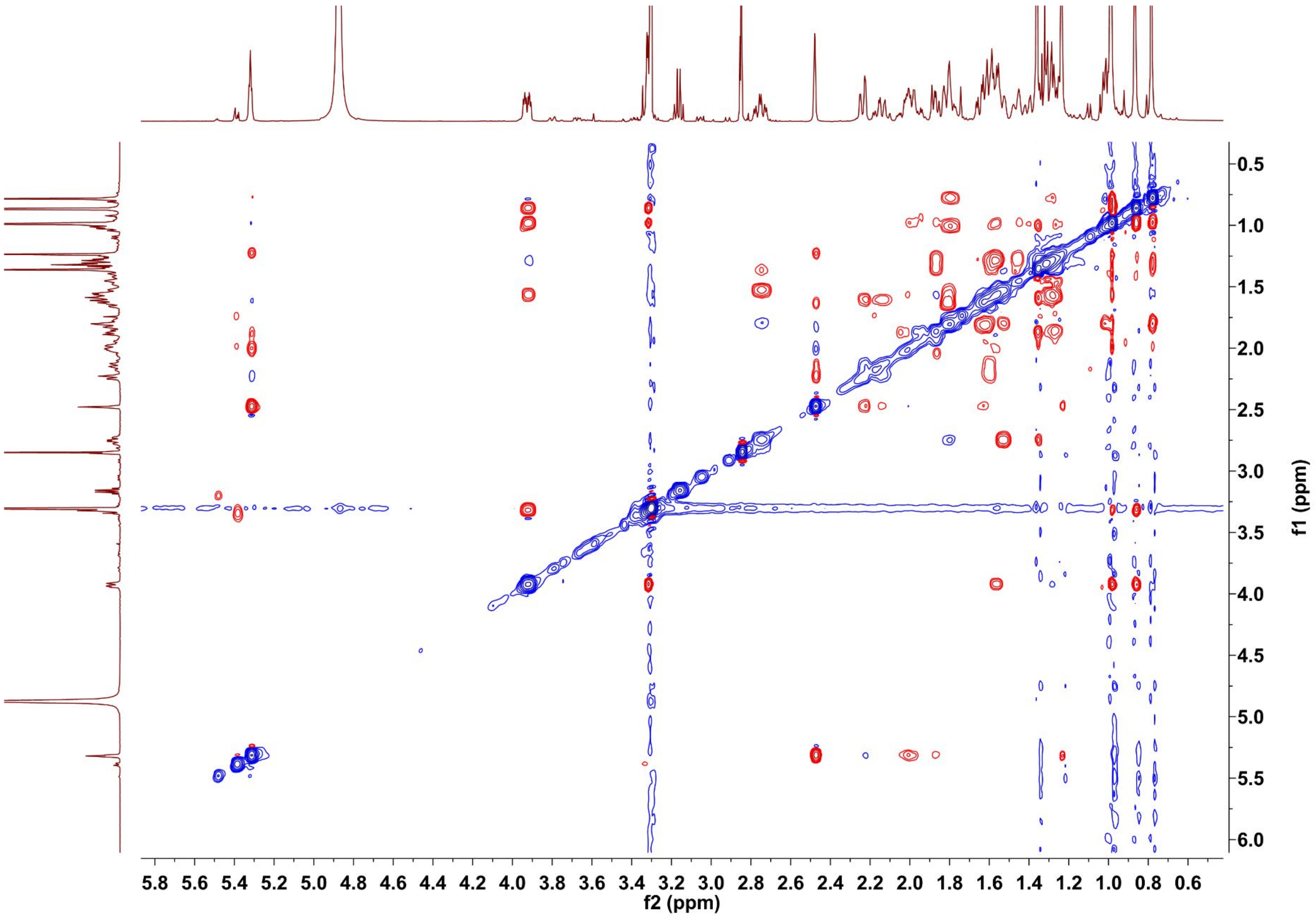

S2-6 ROESY spectrum of **2** in CD_3OD (500 MHz)

Data File: E:\DATA\2022\0729\wcl-8a.lcd

Elmt	Val.	Min	Max	Elmt	Val.	Min	Max	Elmt	Val.	Min	Max	Elmt	Val.	Min	Max	Use Adduct
H	1	5	100	F	1	0	0	Cl	1	0	0	Ag	1	0	0	H
2H	1	0	0	Na	1	0	0	Co	2	0	0	I	3	0	0	
B	3	0	0	Mg	2	0	0	Cu	2	0	0	Ir	3	0	0	
C	4	10	60	Si	4	0	0	Se	2	0	0					
N	3	0	10	P	3	0	0	Br	1	0	5					
O	2	0	30	S	2	0	0	Pd	2	0	0					

Error Margin (ppm):	5	DBE Range:	not fixed	Electron Ions:	both
HC Ratio:	unlimited	Apply N Rule:	no	Use MSn Info:	yes
Max Isotopes:	all	Isotope RI (%):	1.00	Isotope Res:	10000
MSn Iso RI (%):	75.00	MSn Logic Mode:	OR	Max Results:	30

Event#: 2 MS(E-) Ret. Time : 0.333 -> 0.680 Scan# : 52 -> 104

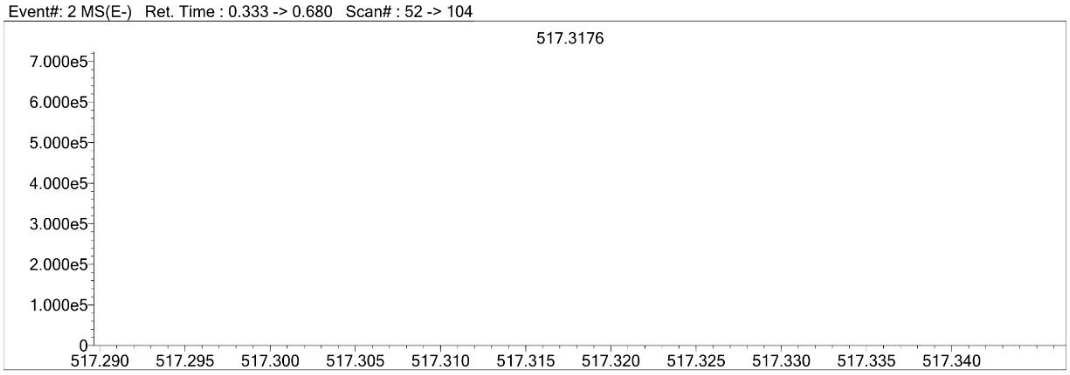

Measured region for 517.3176 m/z

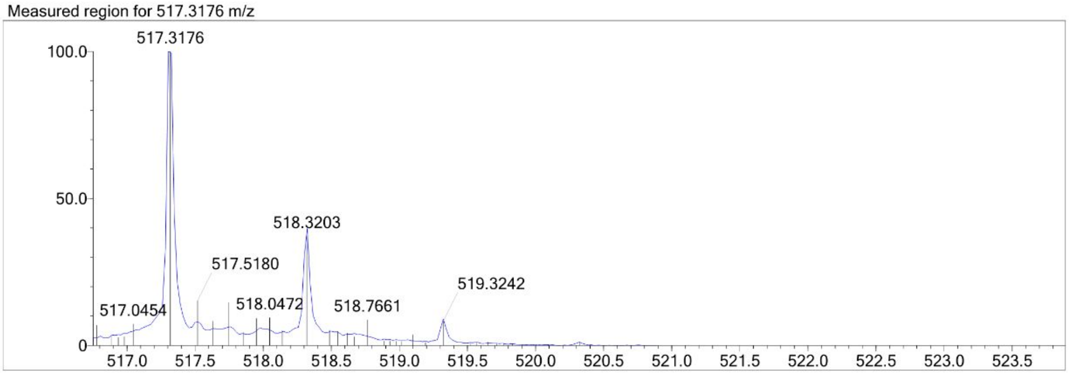

C30 H46 O7 [M-H]- : Predicted region for 517.3171 m/z

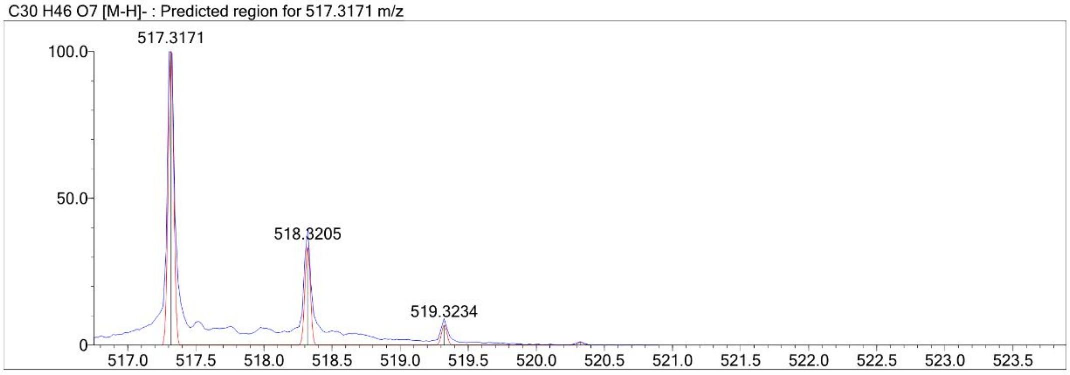

Formula (M)	Ion	Meas. m/z	Pred. m/z	Df. (mDa)	Df. (ppm)	DBE
C30 H46 O7	[M-H]-	517.3176	517.3171	0.5	0.97	8.0

S2-7 HRESIMS spectrum of compound **2**

Rudolph Research Analytical

This sample was measured on an Autopol VI, Serial #91058
Manufactured by Rudolph Research Analytical, Hackettstown, NJ, USA.

Measurement Date : Wednesday, 27-JUL-2022

Set Temperature : 25.0

Time Delay : Disabled

Delay between Measurement : Disabled

n	Average	Std.Dev.	% RSD	Maximum	Minimum
5	4.62	0.34	7.35	5.04	4.34

S.No	Sample ID	Time	Result	Scale	OR °Arc	WLG.nm	Lg.mm	Conc.g/100ml	Temp.
1	WCL-8A	07:57:55 PM	4.34	SR Plus	0.043	589	100.00	0.120	24.7
2	WCL-8A	07:58:02 PM	4.34	SR Plus	0.043	589	100.00	0.120	24.8
3	WCL-8A	07:58:08 PM	4.44	SR Plus	0.044	589	100.00	0.120	24.8
4	WCL-8A	07:58:14 PM	4.94	SR Plus	0.049	589	100.00	0.120	24.9
5	WCL-8A	07:58:21 PM	5.04	SR Plus	0.050	589	100.00	0.120	24.9

Page 1 of 1

S2-8 OR spectrum of compound **2** in MeOH

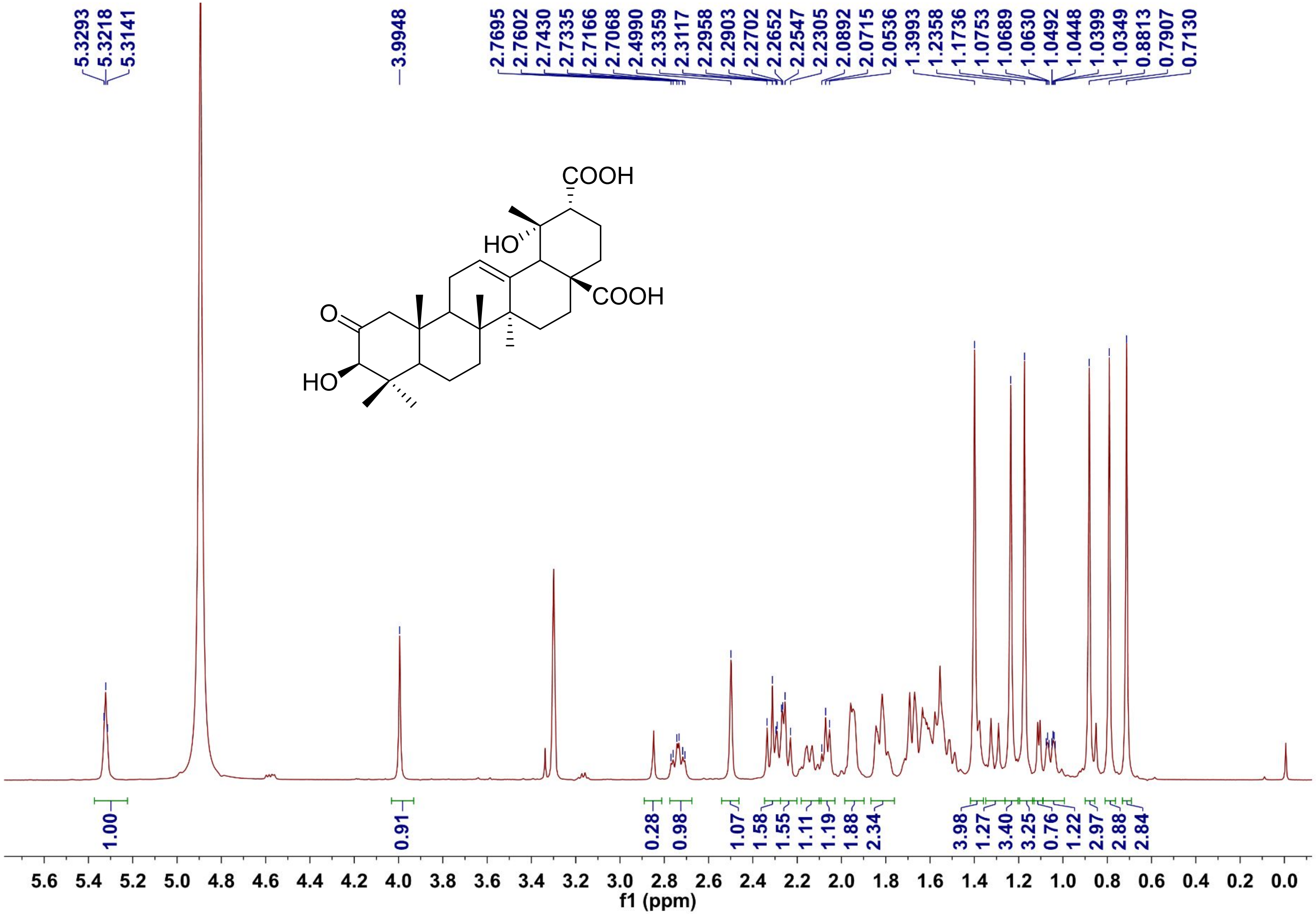

S3-1 ^{1}H NMR spectrum of compound **3** in CD_3OD (500 MHz)

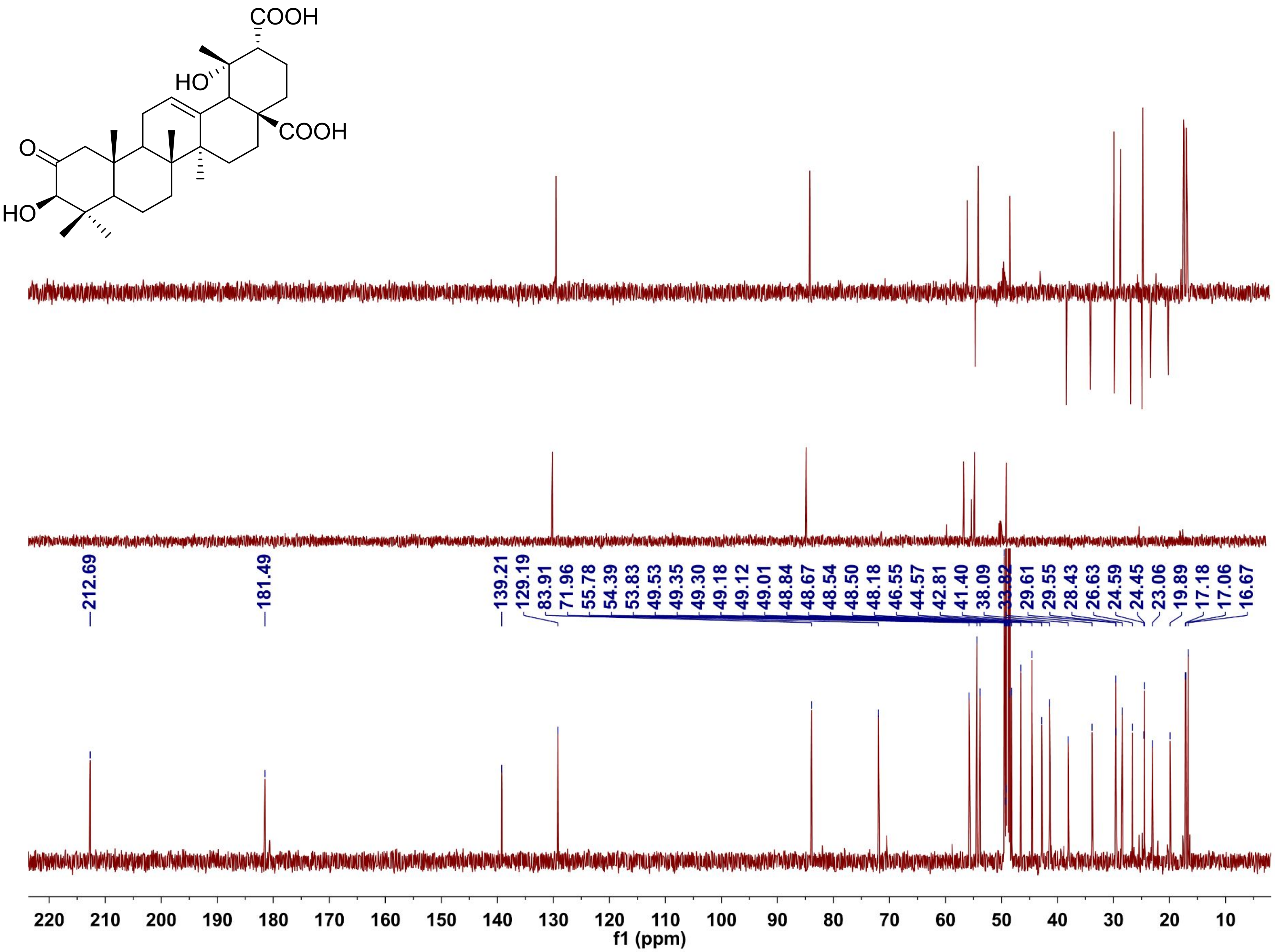

S3-2 ^{13}C NMR spectrum of compound **3** in CD_3OD (125 MHz)

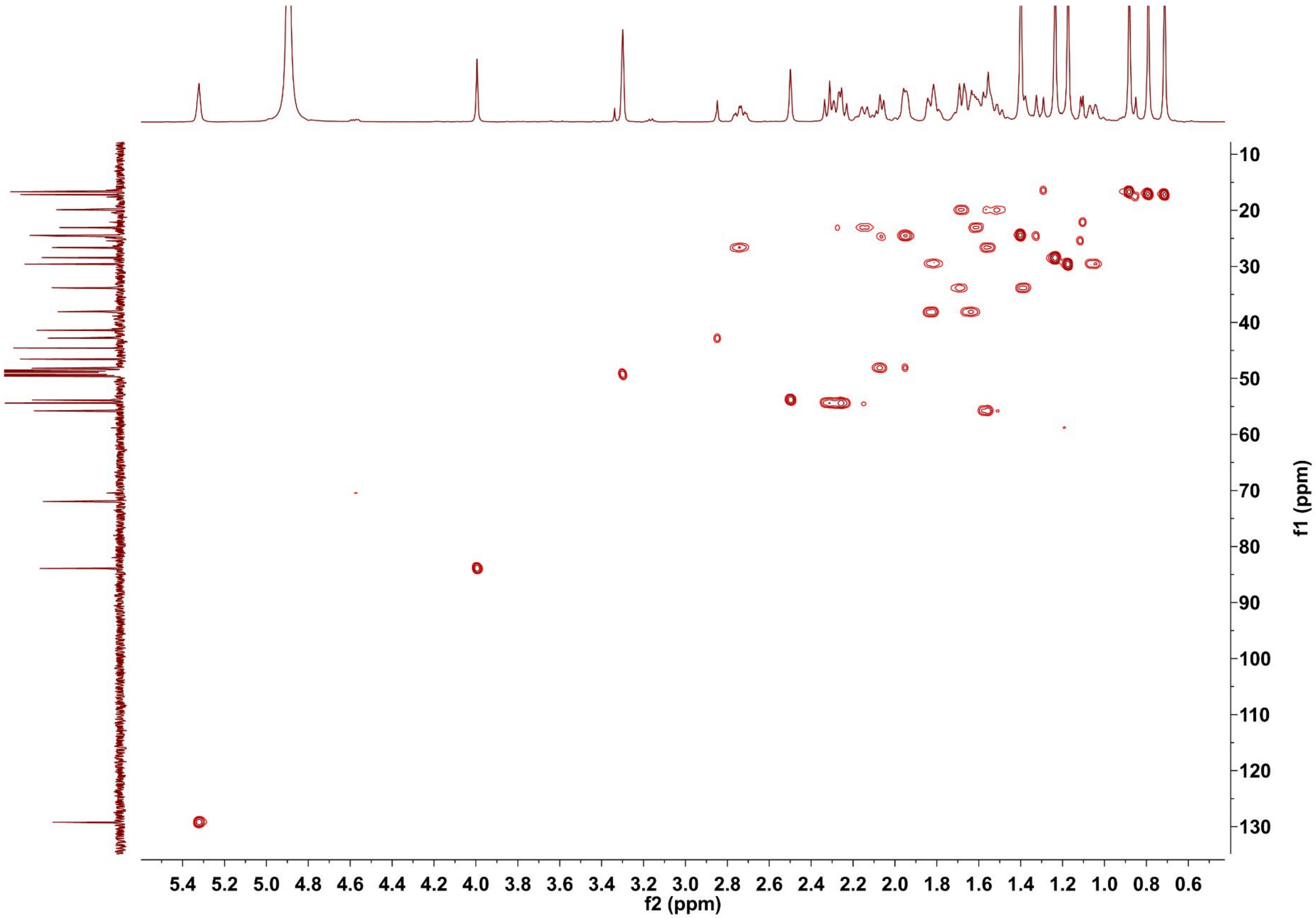

S3-3 HSQC spectrum of compound **3** in CD_3OD (125 MHz)

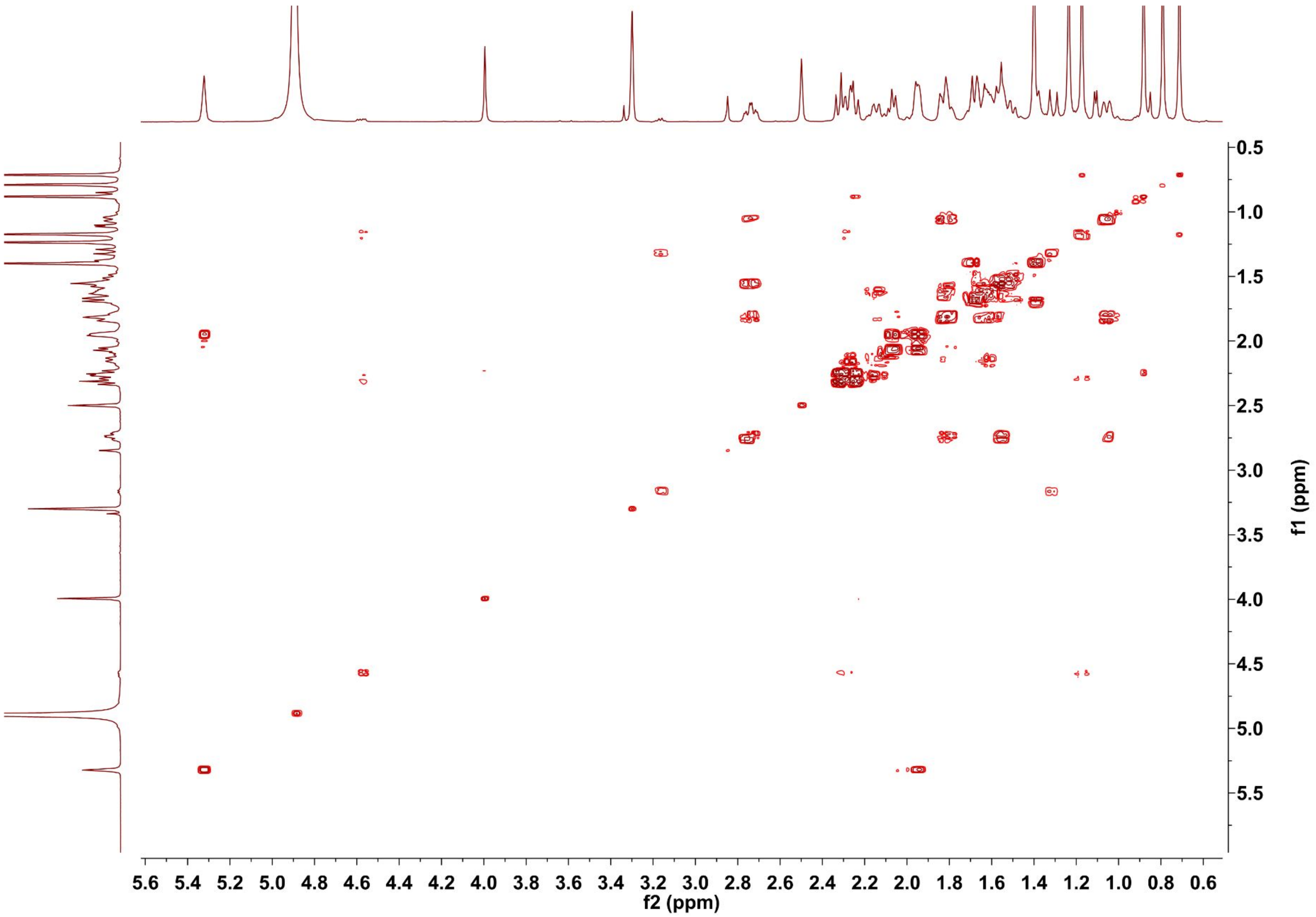

S3-4 ^{1}H-^{1}H COSY spectrum of compound **3** in CD_3OD (500 MHz)

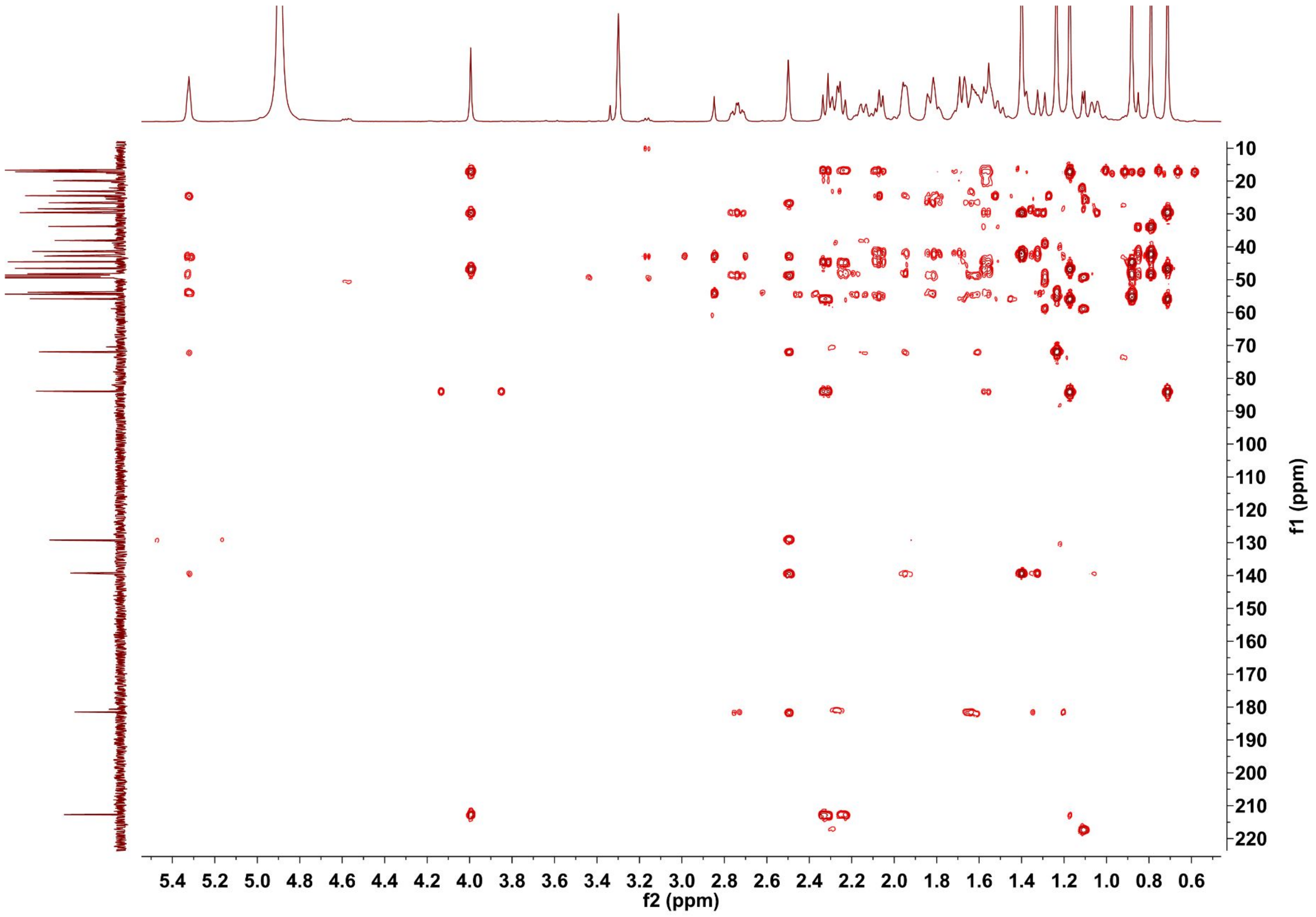

S3-5 HMBC spectrum of compound **3** in CD_3OD (125 MHz)

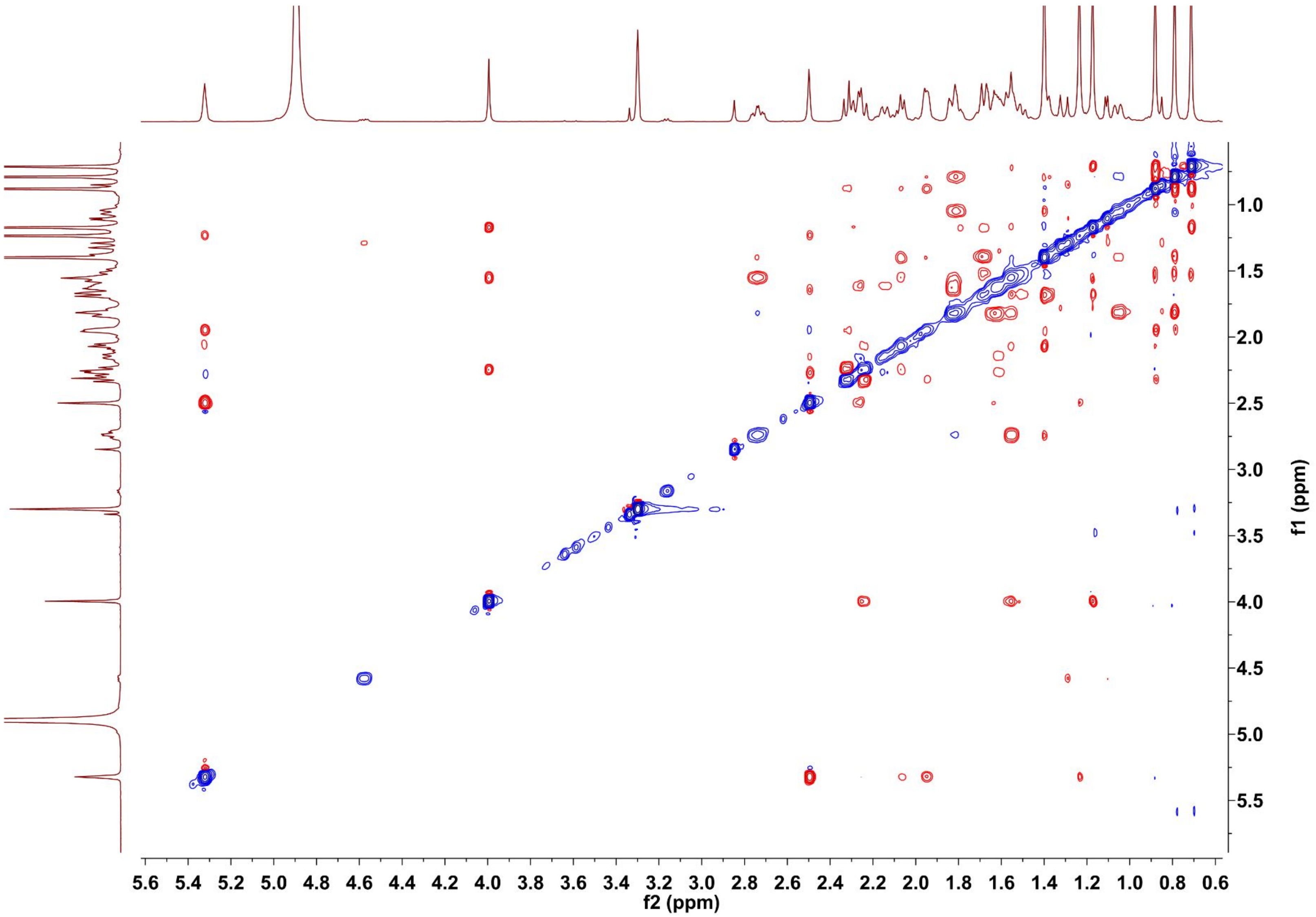

S3-6 ROESY spectrum of compound **3** in CD_3OD (500 MHz)

Formula Predictor Report - WCL-6c.lcd Page 1 of 1

Data File: E:\DATA\2022\0301\WCL-6c.lcd

Elmt	Val.	Min	Max	Elmt	Val.	Min	Max	Elmt	Val.	Min	Max	Elmt	Val.	Min	Max	Use Adduct
H	1	5	100	F	1	0	0	Cl	1	0	0	Ag	1	0	0	H
2H	1	0	0	Na	1	0	0	Co	2	0	0	I	3	0	0	
B	3	0	0	Mg	2	0	0	Cu	2	0	0	Ir	3	0	0	
C	4	10	60	Si	4	0	0	Se	2	0	0					
N	3	0	10	P	3	0	0	Br	1	0	5					
O	2	0	30	S	2	0	0	Pd	2	0	0					

Error Margin (ppm): 5
HC Ratio: unlimited
Max Isotopes: all
MSn Iso RI (%): 75.00

DBE Range: not fixed
Apply N Rule: no
Isotope RI (%): 1.00
MSn Logic Mode: OR

Electron Ions: both
Use MSn Info: yes
Isotope Res: 10000
Max Results: 30

Event#: 2 MS(E-) Ret. Time : 0.413 Scan# : 64

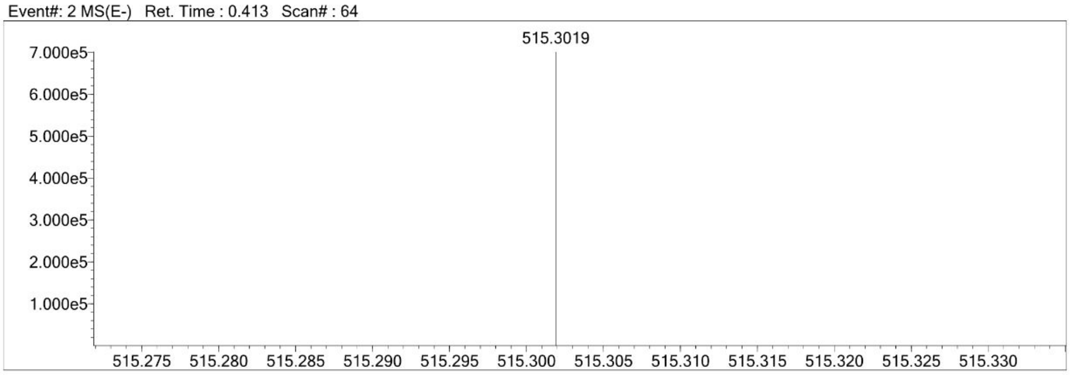

Measured region for 515.3019 m/z

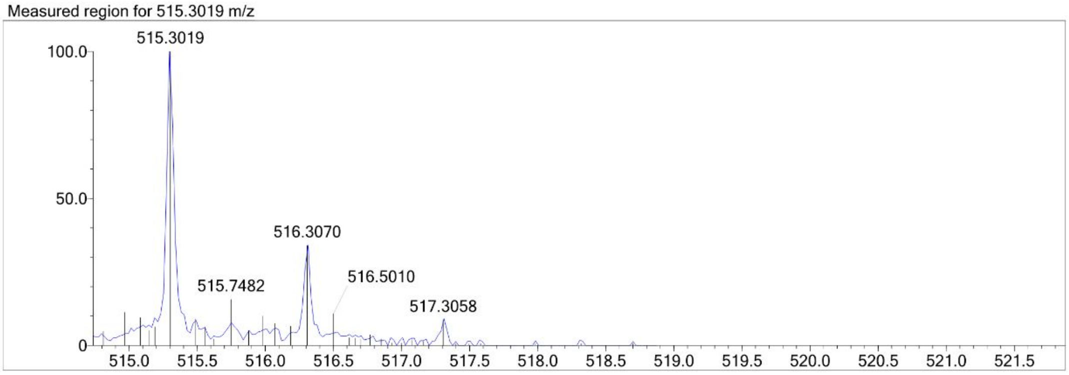

C30 H44 O7 [M-H]- : Predicted region for 515.3014 m/z

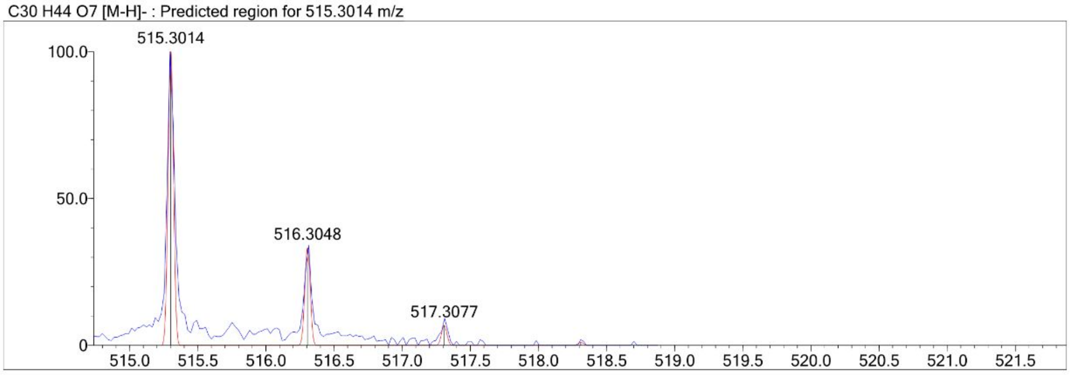

Formula (M)	Ion	Meas. m/z	Pred. m/z	Df. (mDa)	Df. (ppm)	DBE
C30 H44 O7	[M-H]-	515.3019	515.3014	0.5	0.97	9.0

S3-7 HRESIMS spectrum of compound **3**

Rudolph Research Analytical

This sample was measured on an Autopol VI, Serial #91058
Manufactured by Rudolph Research Analytical, Hackettstown, NJ, USA.

Measurement Date : Wednesday, 27-JUL-2022

Set Temperature : 25.0

Time Delay : Disabled

Delay between Measurement : Disabled

n	Average	Std.Dev.	% RSD	Maximum	Minimum
5	56.00	0.50	0.89	56.36	55.45

S.No	Sample ID	Time	Result	Scale	OR °Arc	WLG.nm	Lg.mm	Conc.g/100ml	Temp.
1	WCL-6C	07:52:31 PM	55.45	SR	0.061	589	100.00	0.110	24.7
2	WCL-6C	07:52:38 PM	55.45	SR	0.061	589	100.00	0.110	24.8
3	WCL-6C	07:52:44 PM	56.36	SR	0.062	589	100.00	0.110	24.8
4	WCL-6C	07:52:50 PM	56.36	SR	0.062	589	100.00	0.110	24.9
5	WCL-6C	07:52:57 PM	56.36	SR	0.062	589	100.00	0.110	24.9

S3-8 OR spectrum of compound **3** in MeOH

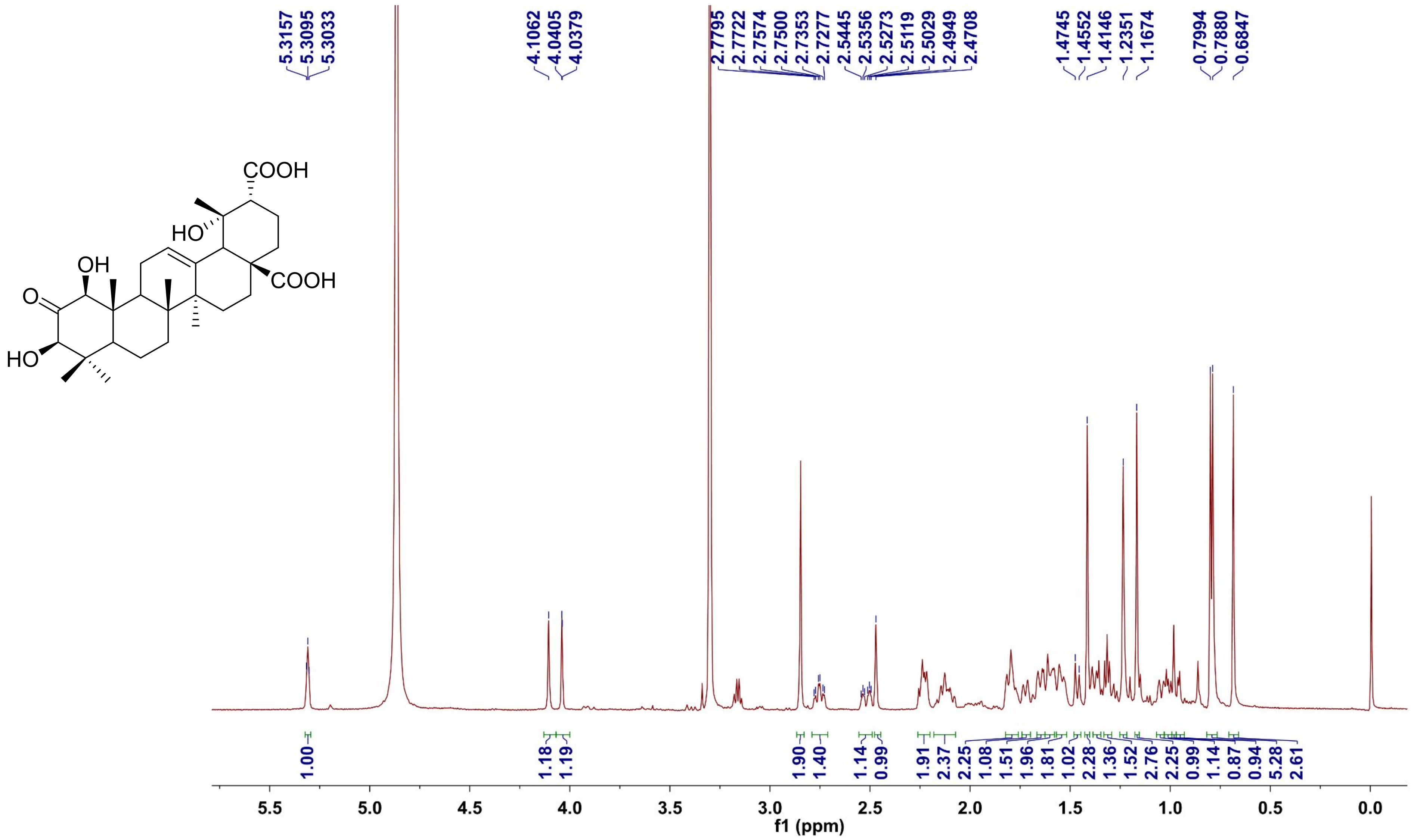

S4-1 ^{1}H NMR spectrum of compound **4** in CD_3OD (600 MHz)

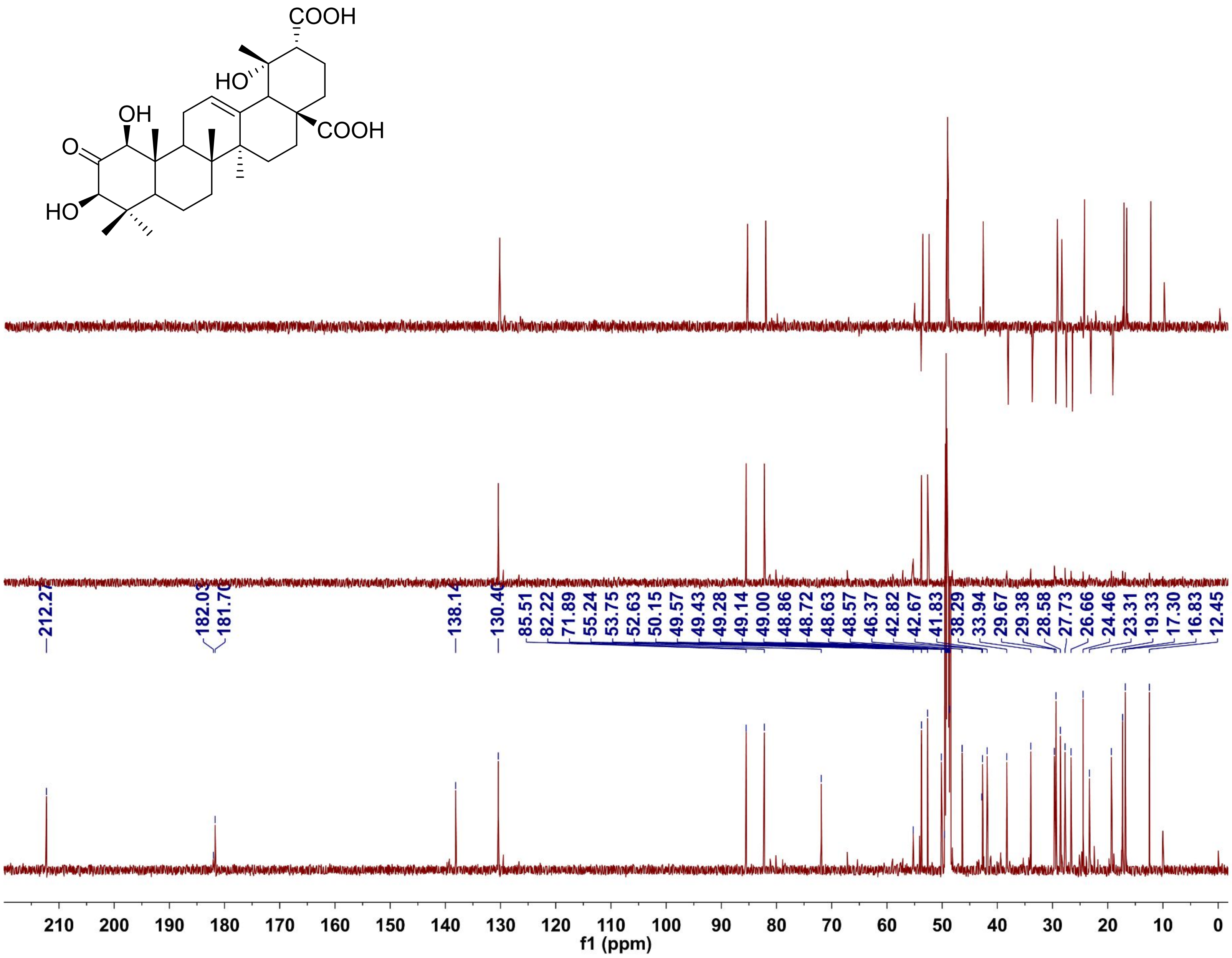

S4-2 ^{13}C NMR spectrum of compound **4** in CD_3OD (150 MHz)

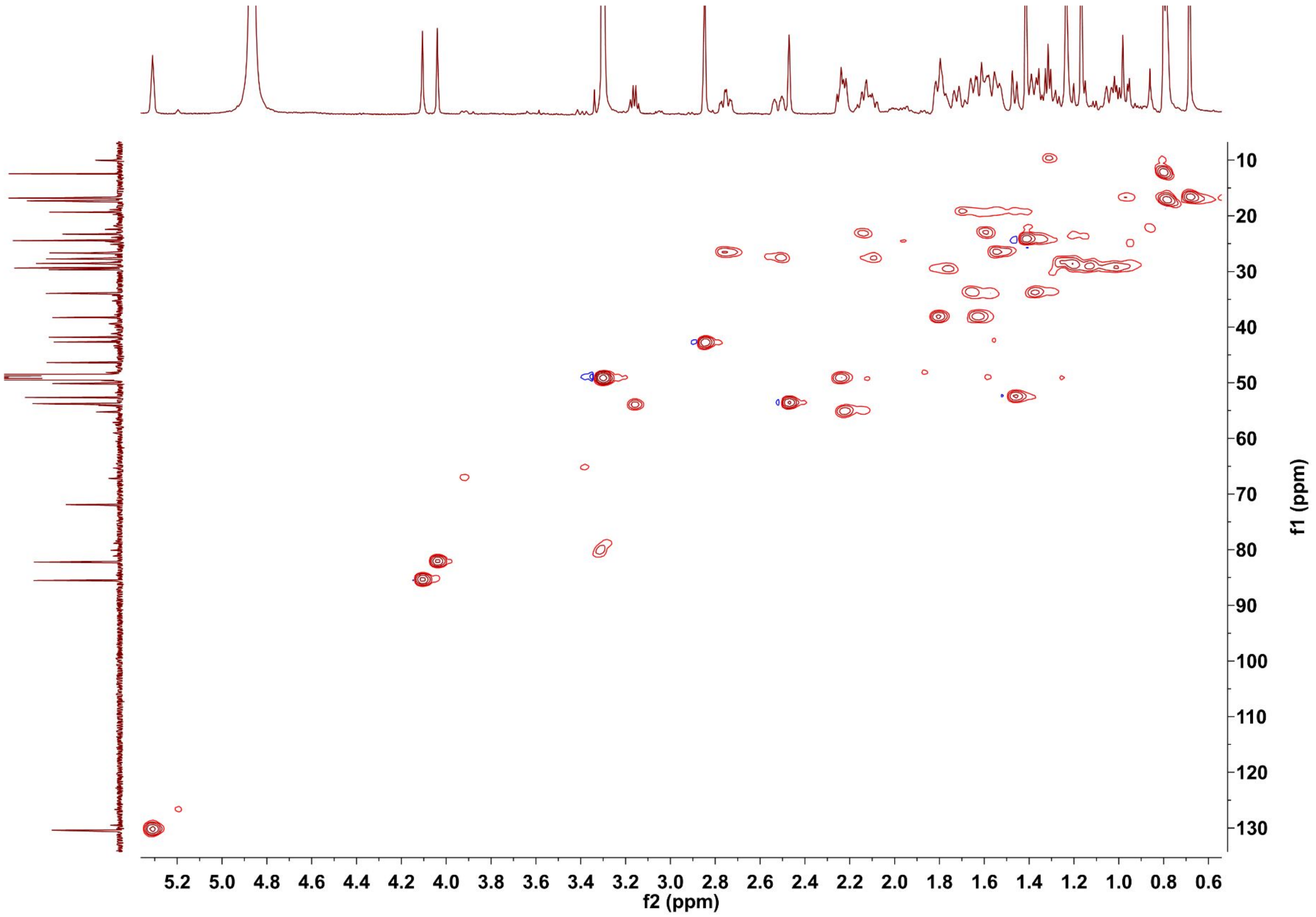

S4-3 HSQC spectrum of compound **4** in CD_3OD (600 MHz)

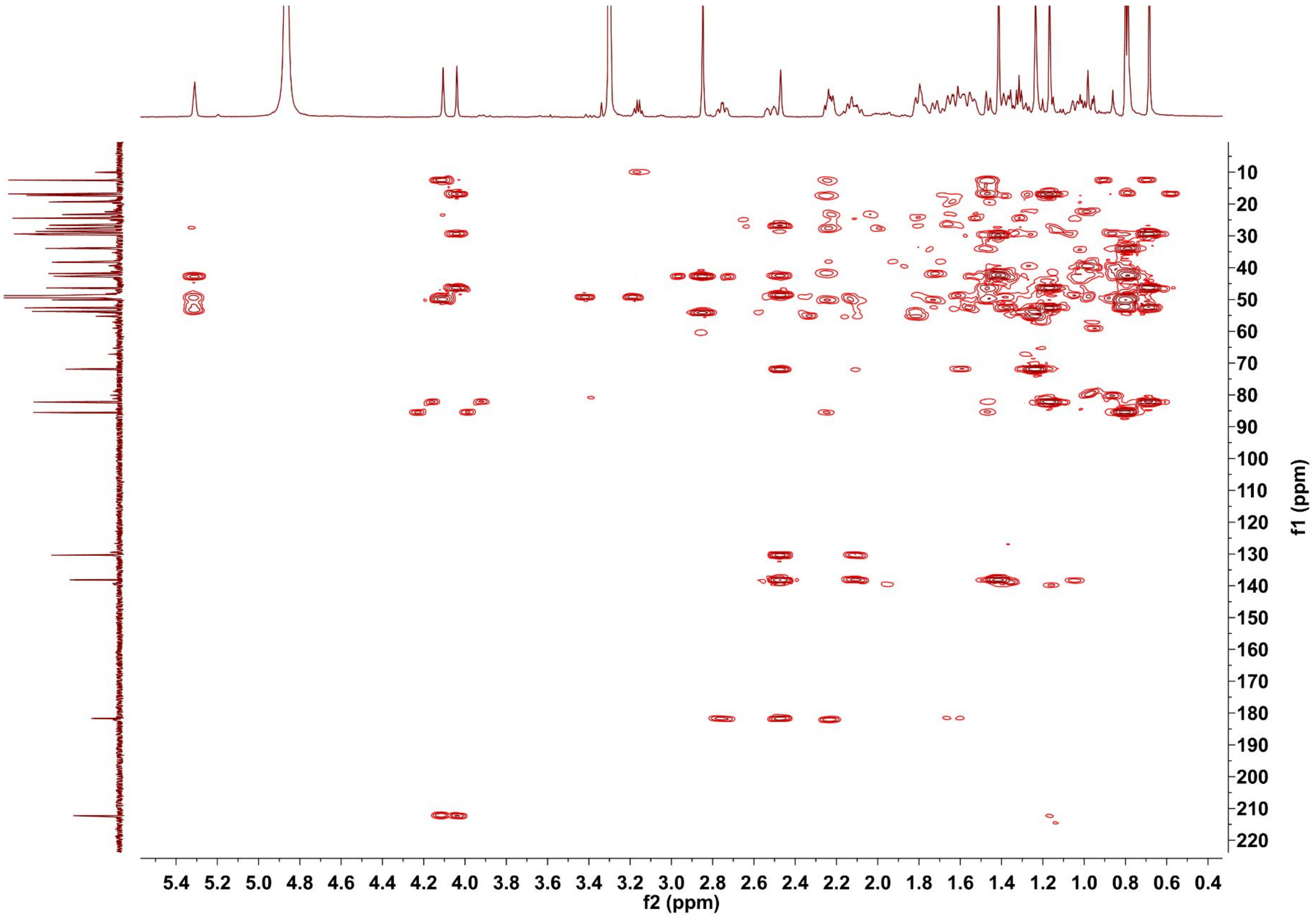

S4-4 HMBC spectrum of compound **4** in CD_3OD (600 MHz)

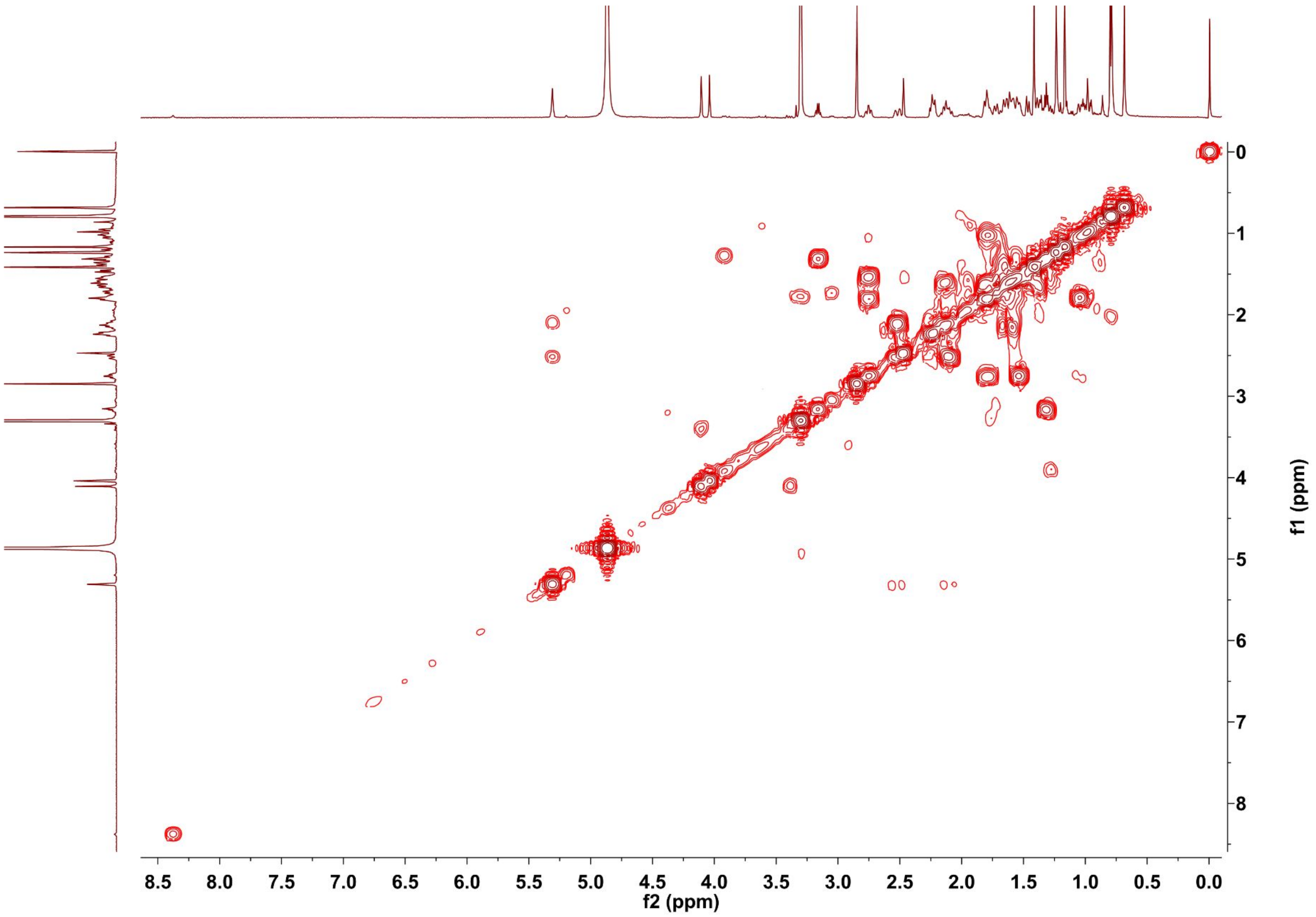

S4-5 ^{1}H-^{1}H COSY spectrum of compound **4** in CD_3OD (600 MHz)

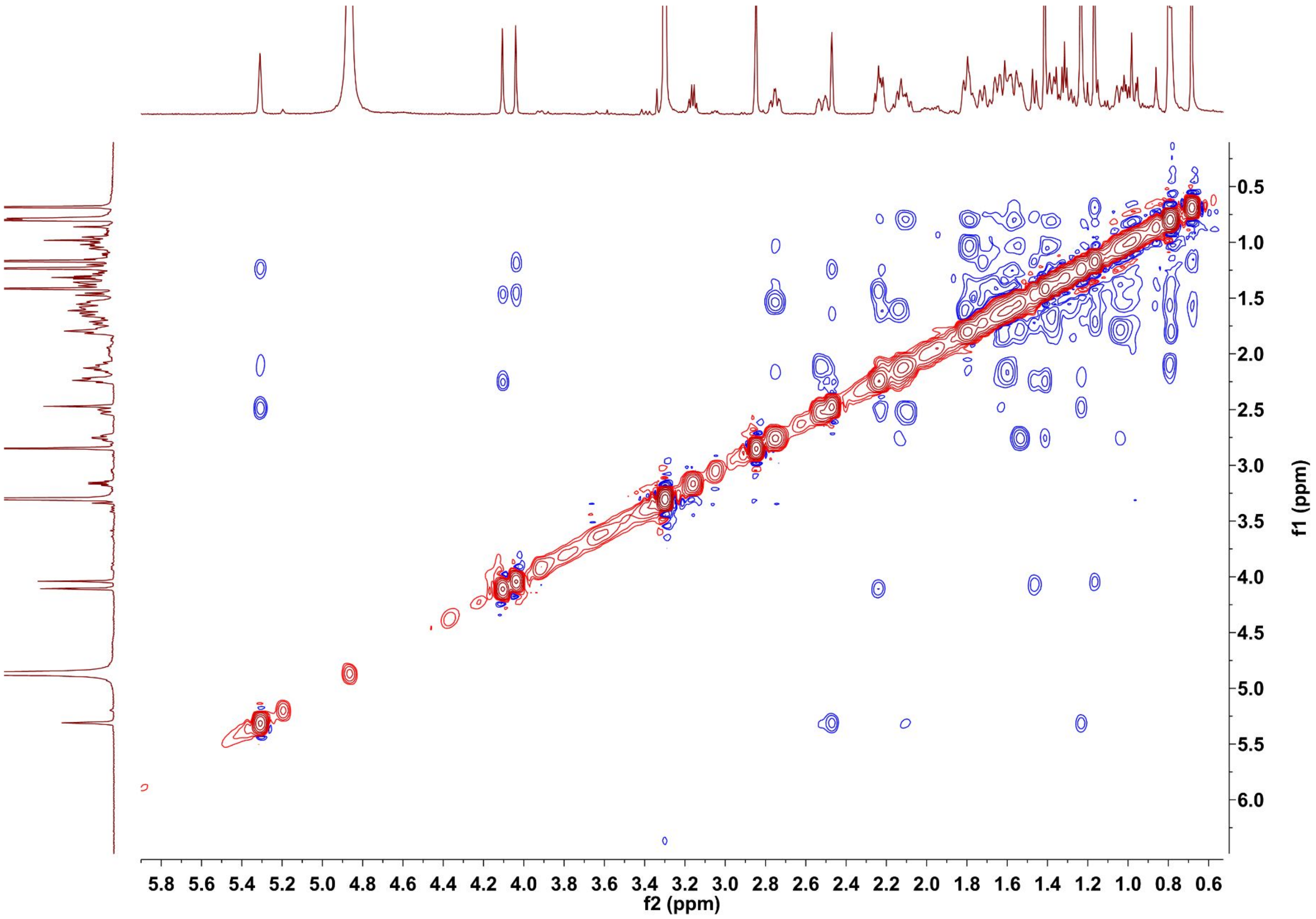

S4-6 ROESY spectrum of compound **4** in CD_3OD (600 MHz)

Data File: E:\DATA\2022\0301\WCL-8b.lcd

Elmt	Val.	Min	Max	Elmt	Val.	Min	Max	Elmt	Val.	Min	Max	Elmt	Val.	Min	Max	Use Adduct
H	1	5	100	F	1	0	0	Cl	1	0	0	Ag	1	0	0	H
2H	1	0	0	Na	1	0	0	Co	2	0	0	I	3	0	0	
B	3	0	0	Mg	2	0	0	Cu	2	0	0	Ir	3	0	0	
C	4	10	60	Si	4	0	0	Se	2	0	0					
N	3	0	10	P	3	0	0	Br	1	0	5					
O	2	0	30	S	2	0	0	Pd	2	0	0					

Error Margin (ppm):	5	DBE Range:	not fixed	Electron Ions:	both
HC Ratio:	unlimited	Apply N Rule:	no	Use MSn Info:	yes
Max Isotopes:	all	Isotope RI (%):	1.00	Isotope Res:	10000
MSn Iso RI (%):	75.00	MSn Logic Mode:	OR	Max Results:	30

Event#: 2 MS(E-) Ret. Time : 0.440 -> 0.453 Scan# : 68 -> 70

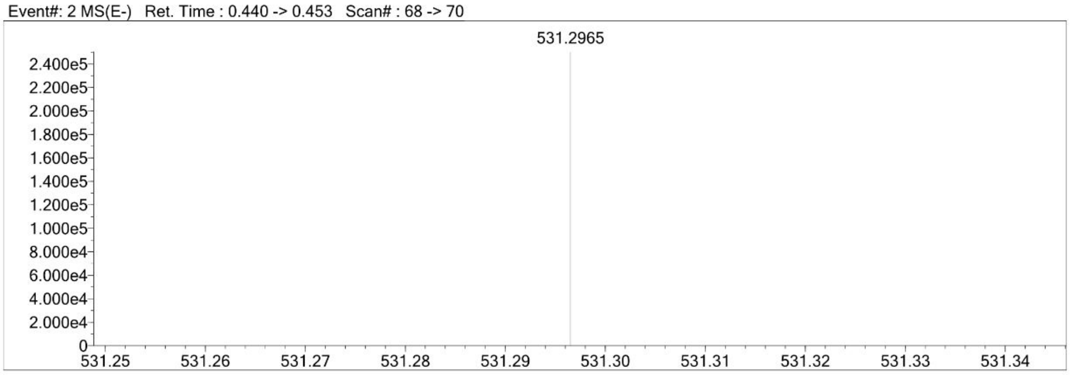

Measured region for 531.2965 m/z

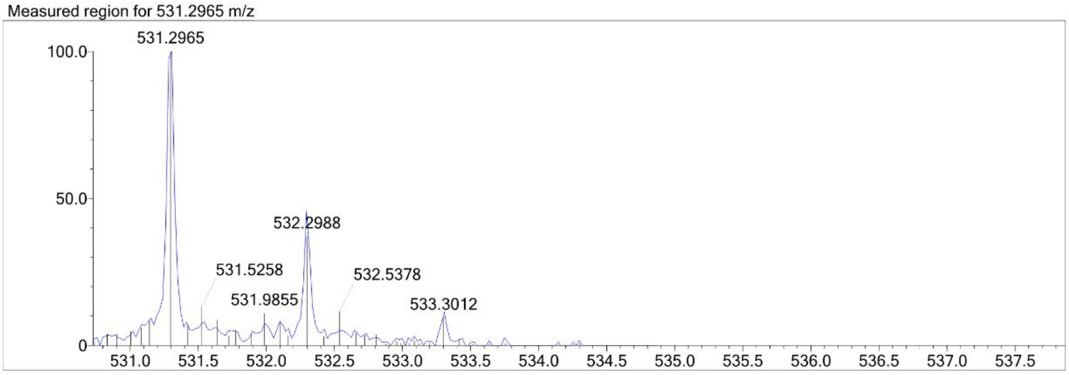

C30 H44 O8 [M-H]- : Predicted region for 531.2963 m/z

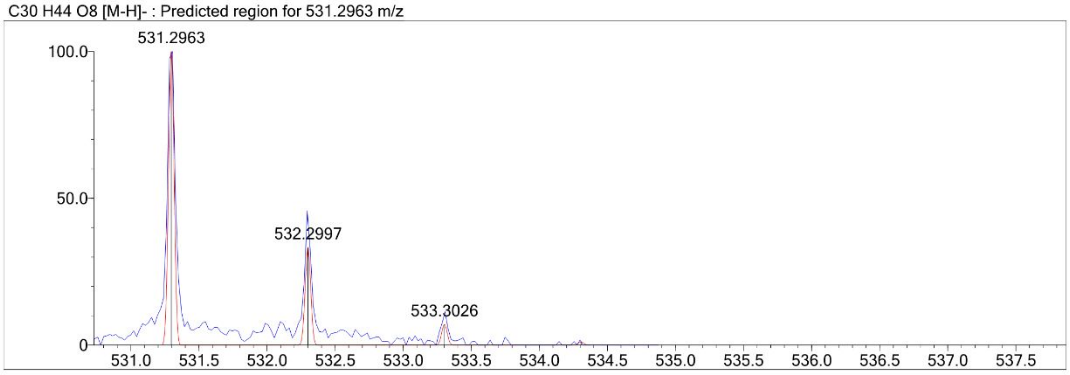

Formula (M)	Ion	Meas. m/z	Pred. m/z	Df. (mDa)	Df. (ppm)	DBE
C30 H44 O8	[M-H]-	531.2965	531.2963	0.2	0.38	9.0

S4-7 HRESIMS spectrum of compound **4**

Rudolph Research Analytical

This sample was measured on an Autopol VI, Serial #91058
Manufactured by Rudolph Research Analytical, Hackettstown, NJ, USA.

Measurement Date : Wednesday, 27-JUL-2022

Set Temperature : 25.0

Time Delay : Disabled

Delay between Measurement : Disabled

n	Average	Std.Dev.	% RSD	Maximum	Minimum
5	55.20	1.10	1.99	56.00	54.00

S.No	Sample ID	Time	Result	Scale	OR °Arc	WLG.nm	Lg.mm	Conc.g/100ml	Temp.
1	WCL-8B	08:08:41 PM	54.00	SR	0.027	589	100.00	0.050	25.0
2	WCL-8B	08:08:52 PM	56.00	SR	0.028	589	100.00	0.050	25.0
3	WCL-8B	08:08:58 PM	56.00	SR	0.028	589	100.00	0.050	25.0
4	WCL-8B	08:09:04 PM	56.00	SR	0.028	589	100.00	0.050	25.0
5	WCL-8B	08:09:11 PM	54.00	SR	0.027	589	100.00	0.050	25.0

Page 1 of 1

S4-8 OR spectrum of compound **4** in MeOH

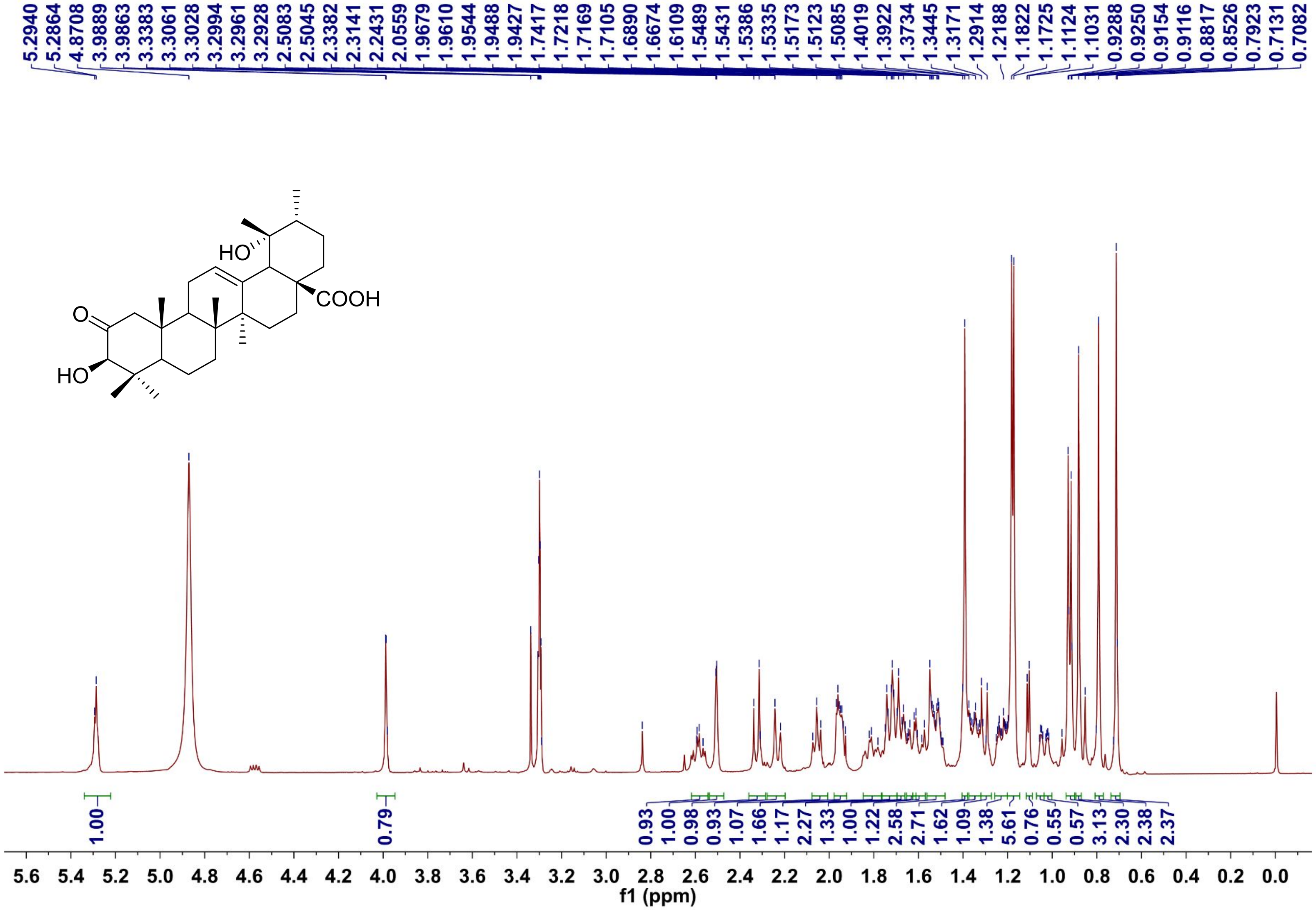

S5-1 ^{1}H NMR spectrum of compound **5** in CD_3OD (500 MHz)

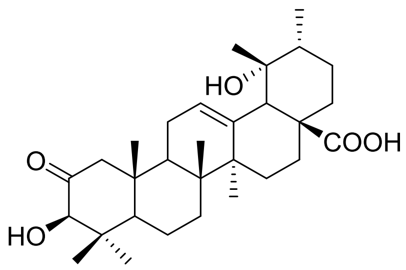

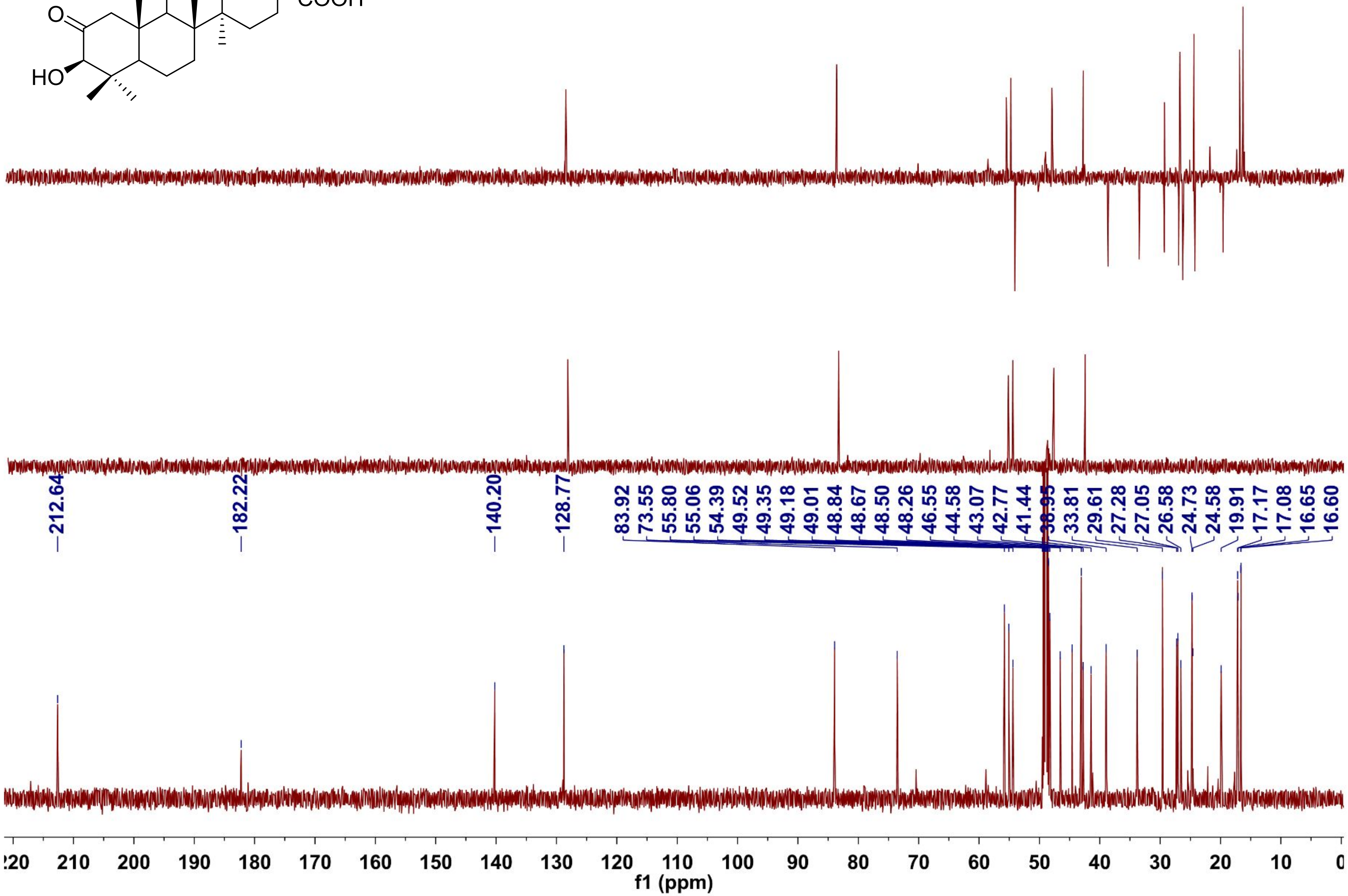

S5-2 ^{13}C NMR spectrum of compound **5** in CD_3OD (125 MHz)

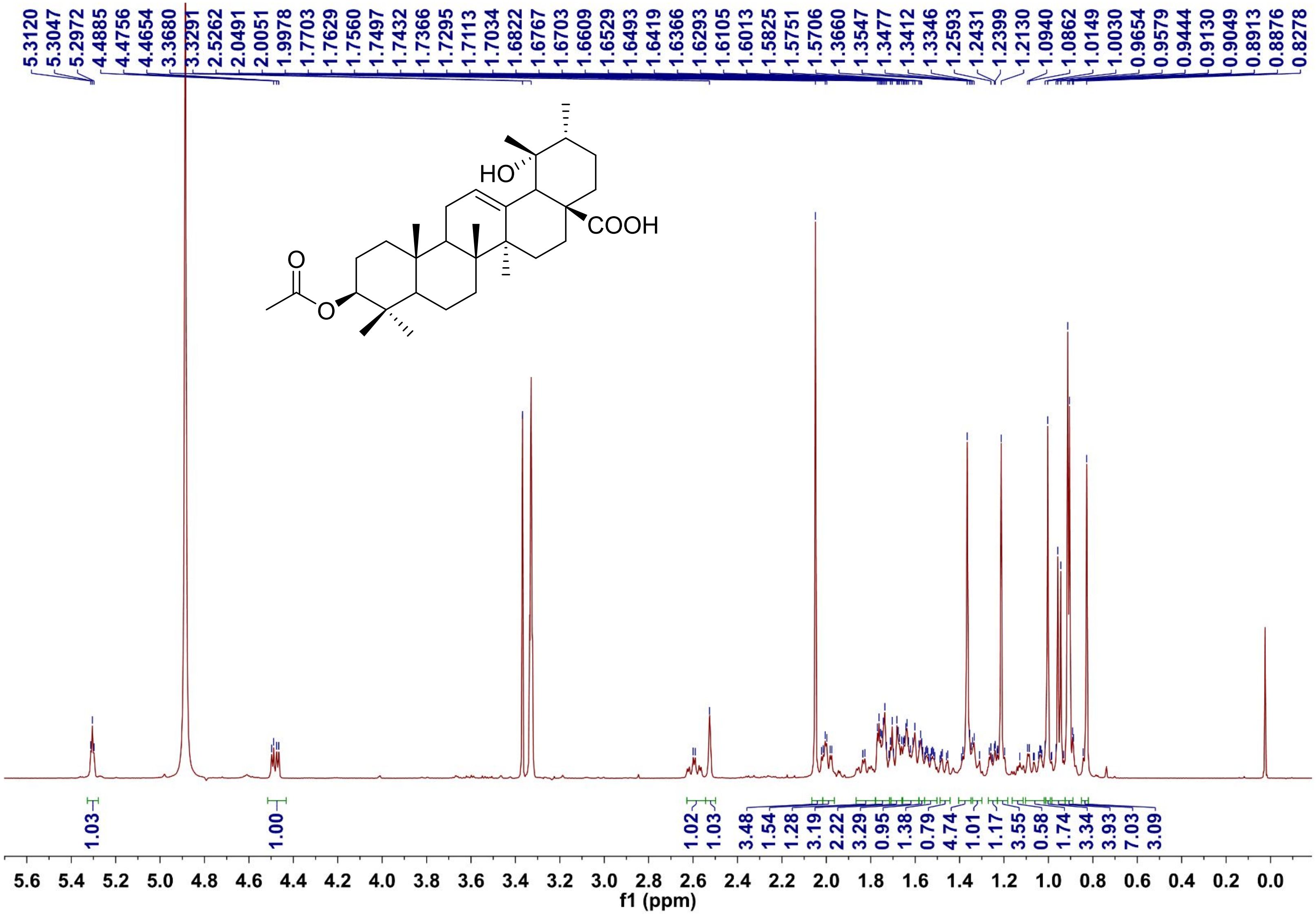

S6-1 ^{1}H NMR spectrum of compound **6** in CD_3OD (500 MHz)

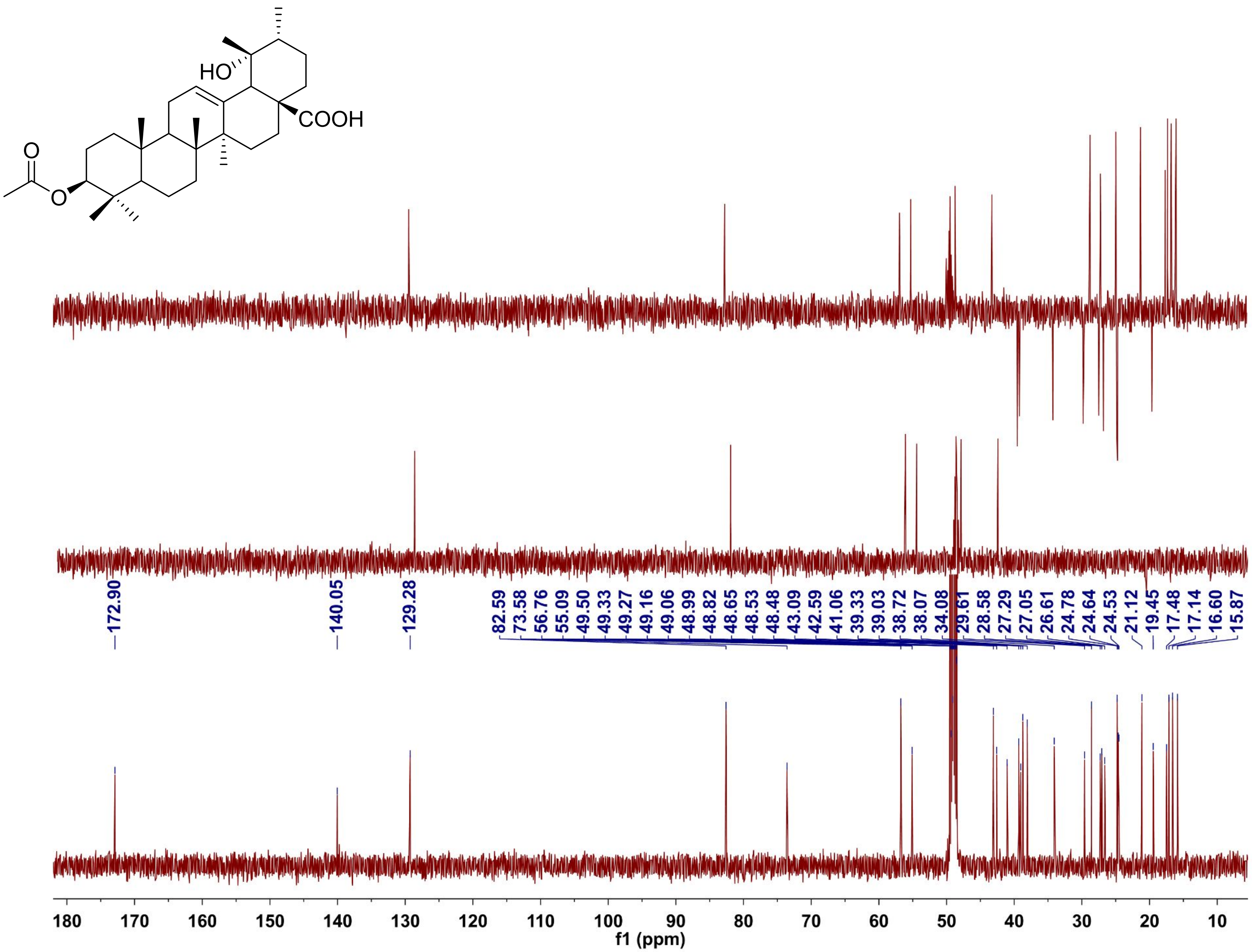

S6-2 ^{13}C NMR spectrum of compound **6** in CD_3OD (125 MHz)

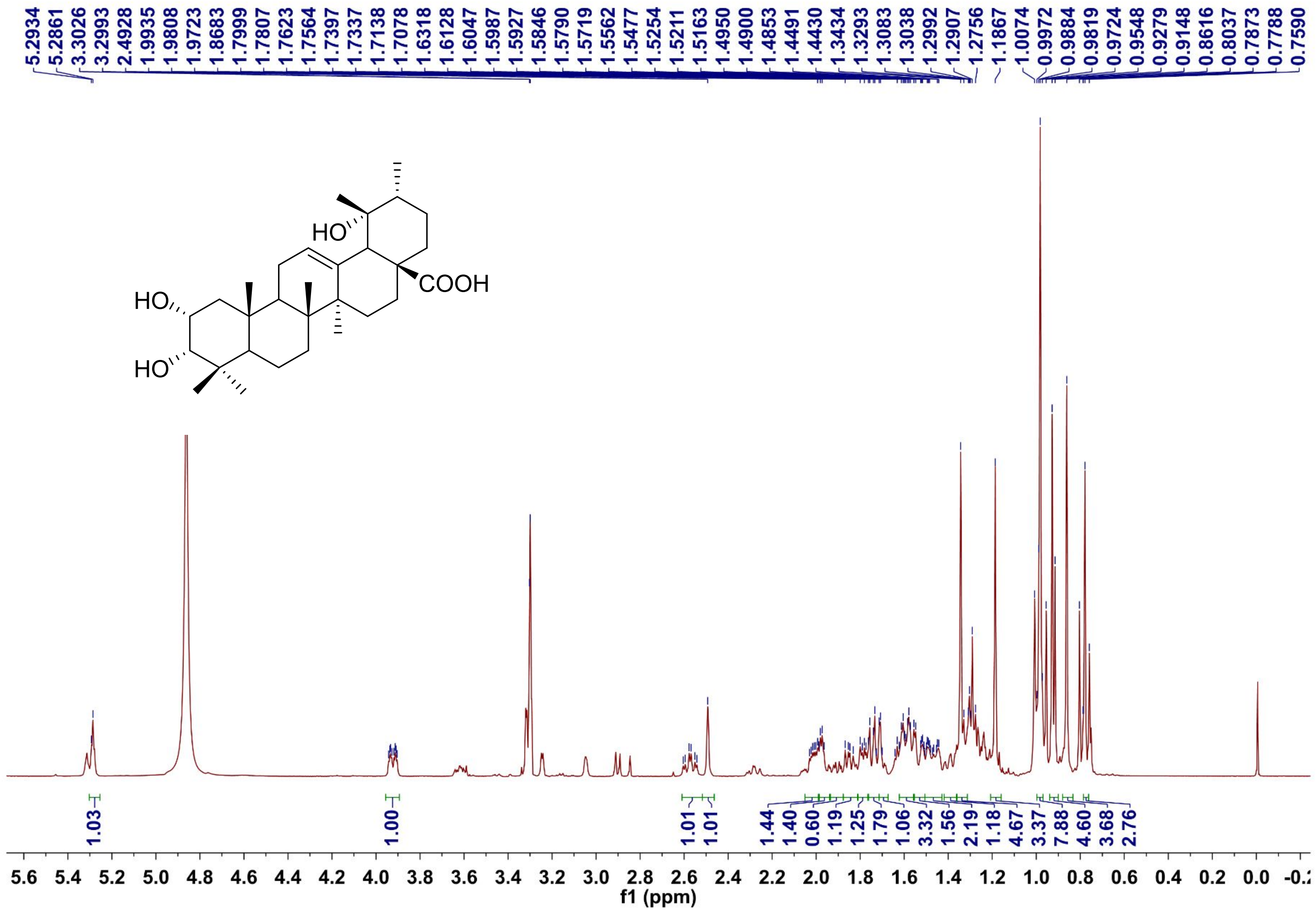

S7-1 ^{1}H NMR spectrum of compound **7** in CD_3OD (500 MHz)

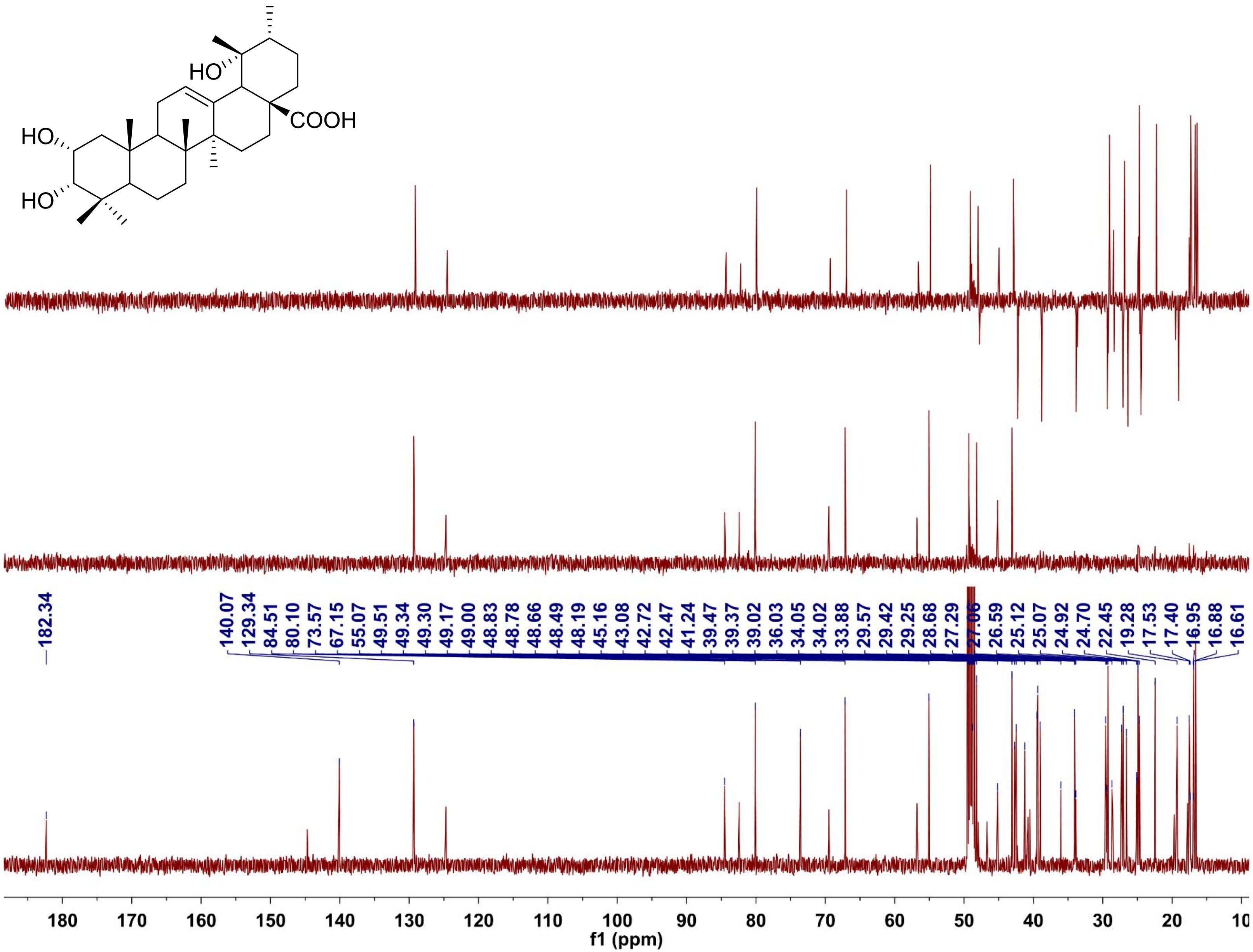

S7-2 ^{13}C NMR spectrum of compound **7** in CD_3ODCD_3(125 MHz)

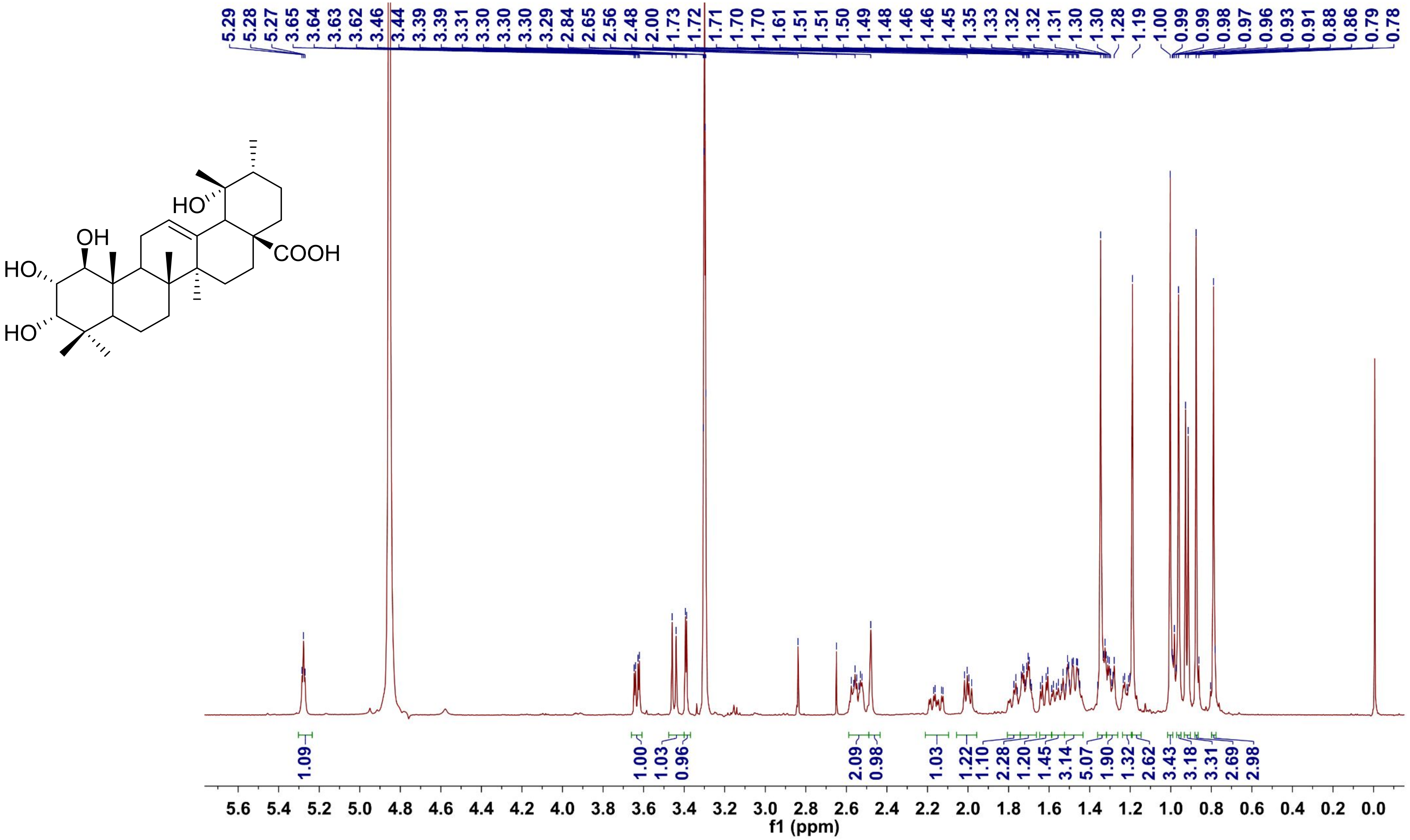

S8-1 ^{1}H NMR spectrum of compound **8** in CD_3OD (500 MHz)

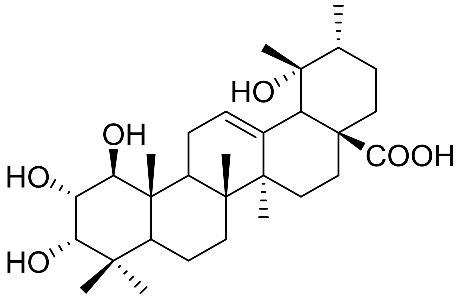

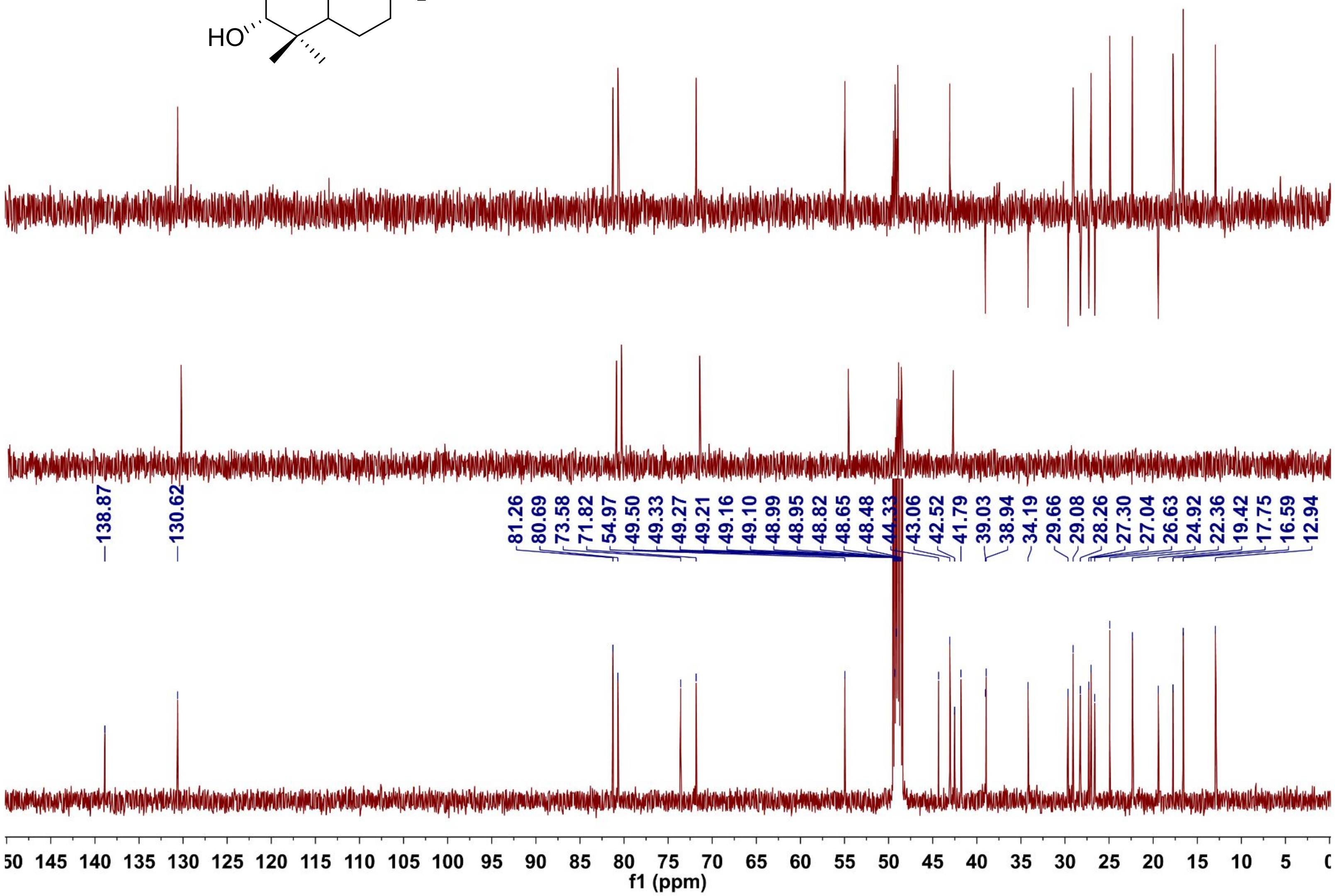

S8-2 ^{13}C NMR spectrum of compound **8** in CD_3OD (125 MHz)

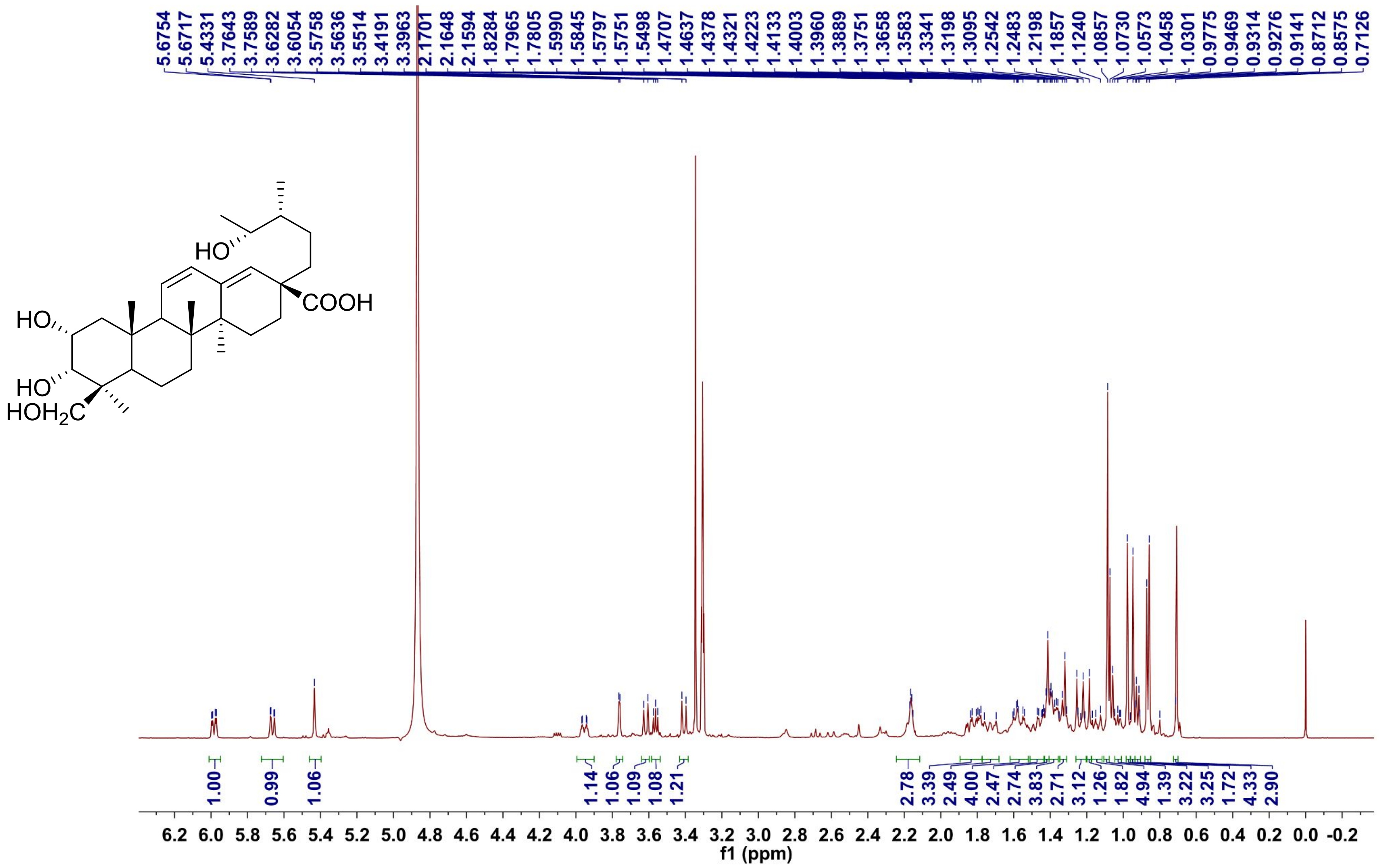

S9-1 ^{1}H NMR spectrum of compound **9** in CD_3OD (500 MHz)

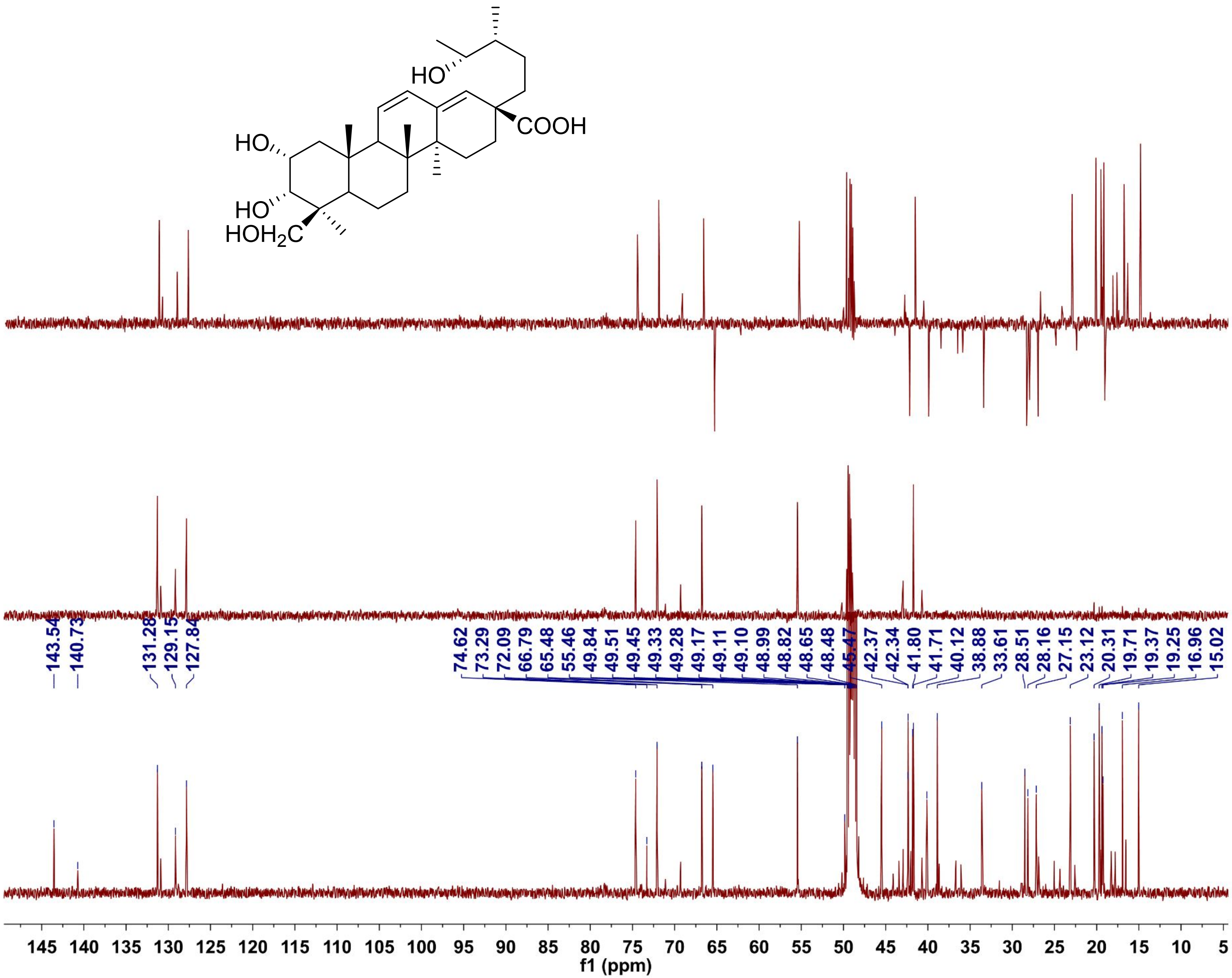

S9-2 ^{13}C NMR spectrum of compound **9** in CD_3OD (125 MHz)

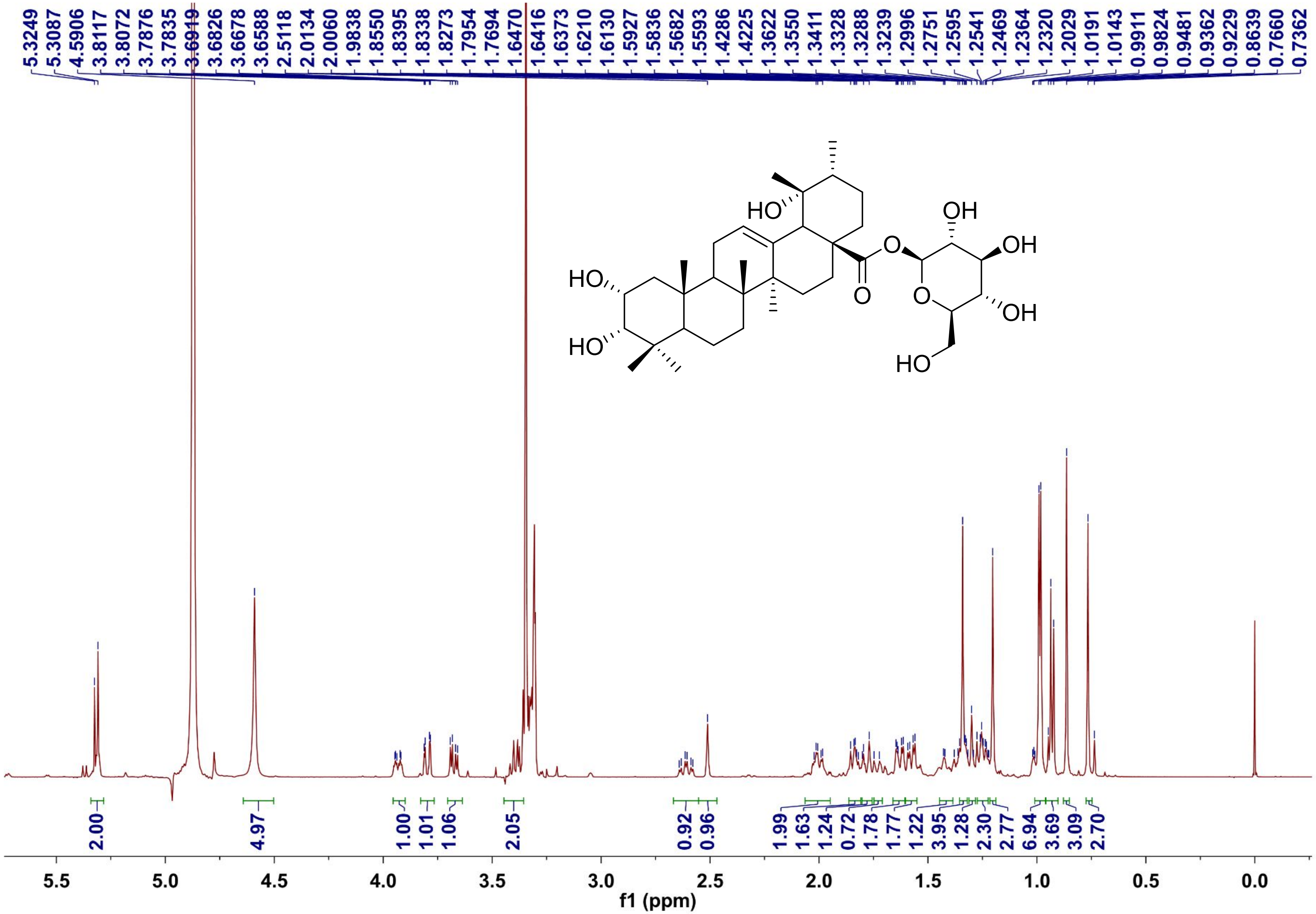

S10-1 ^{1}H NMR spectrum of compound **10** in CD_3OD (500 MHz)

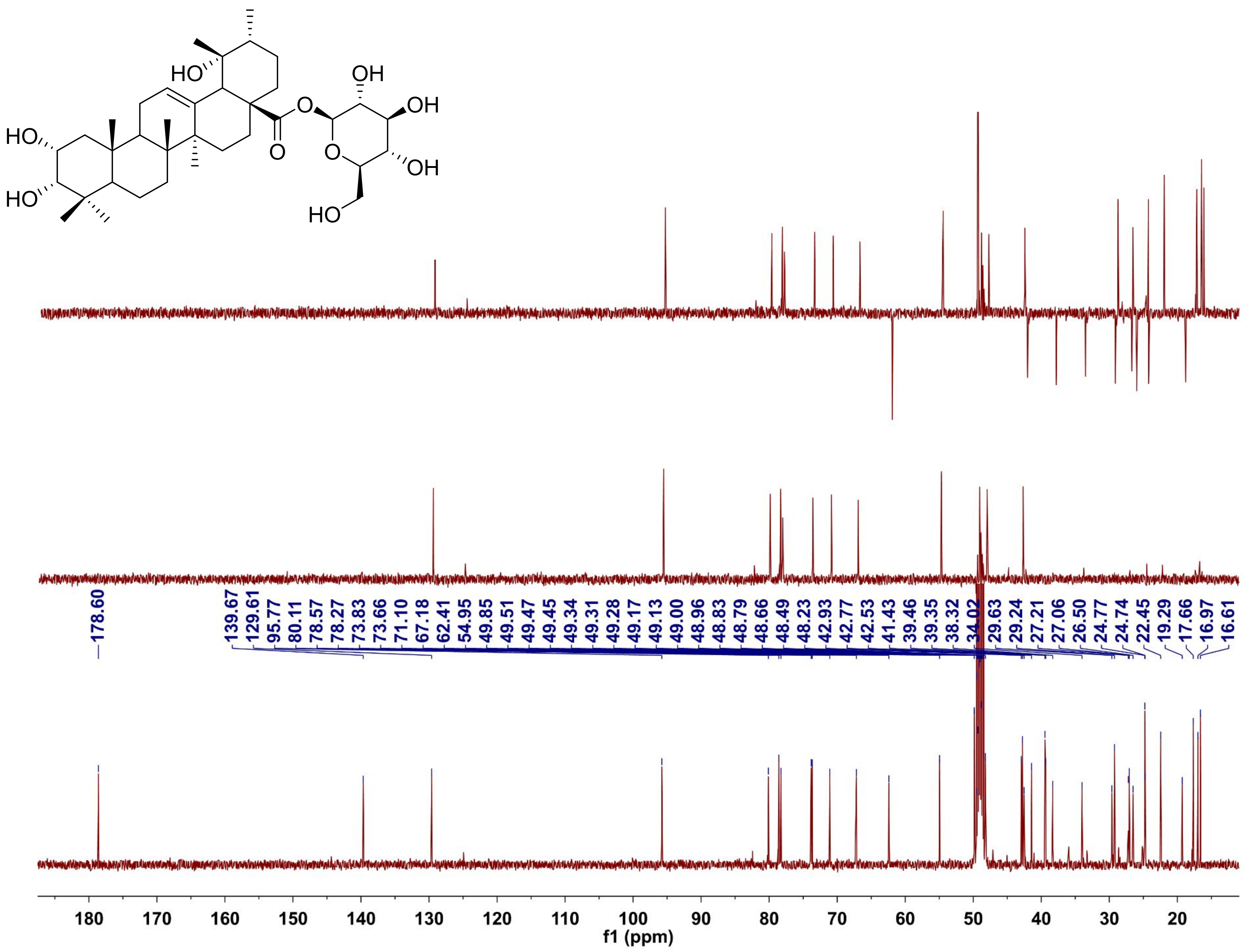

S10-2 ^{13}C NMR spectrum of compound **10** in CD_3OD (125 MHz)

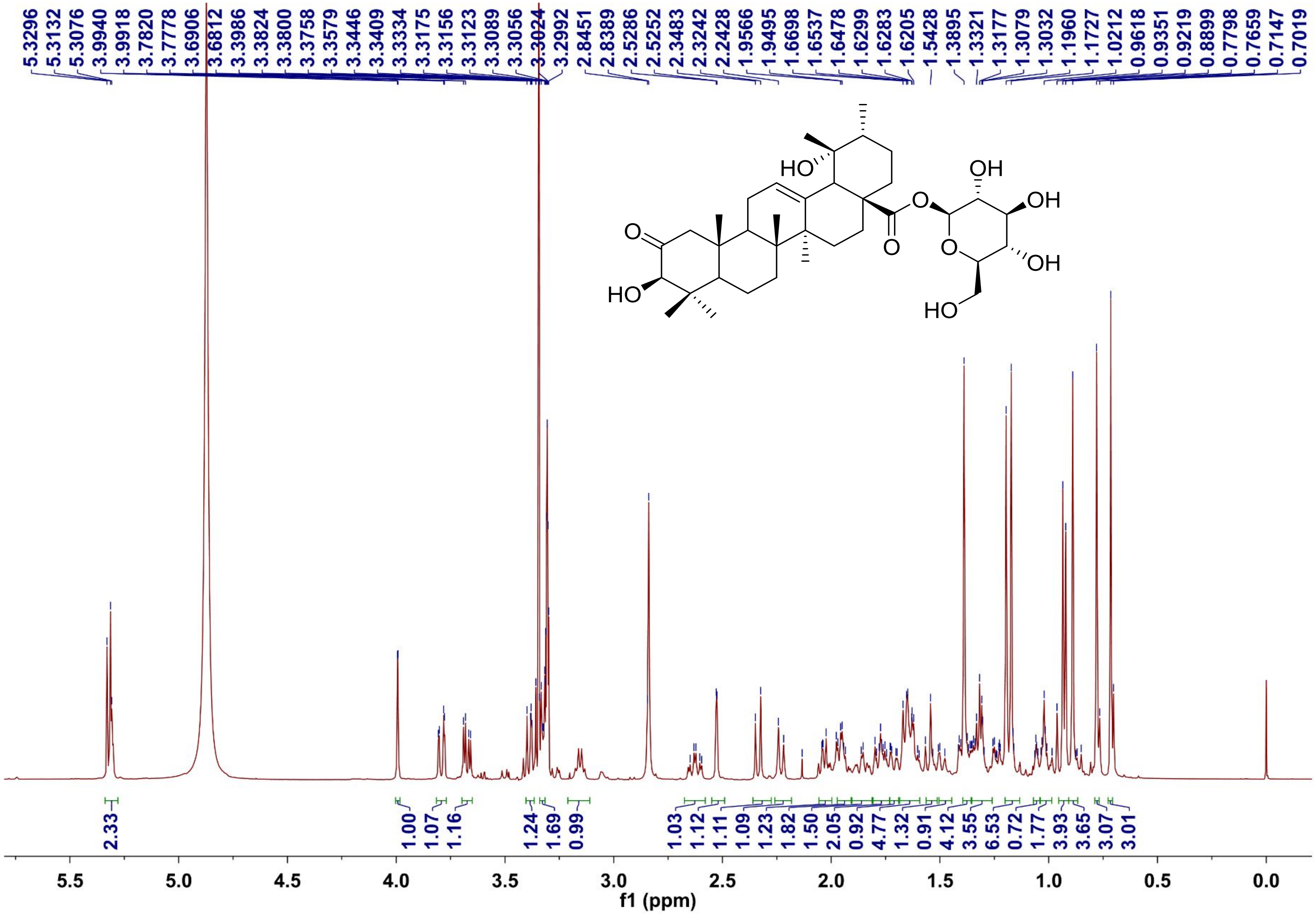

S11-1 ^{1}H NMR spectrum of compound **11** in CD_3OD (500 MHz)

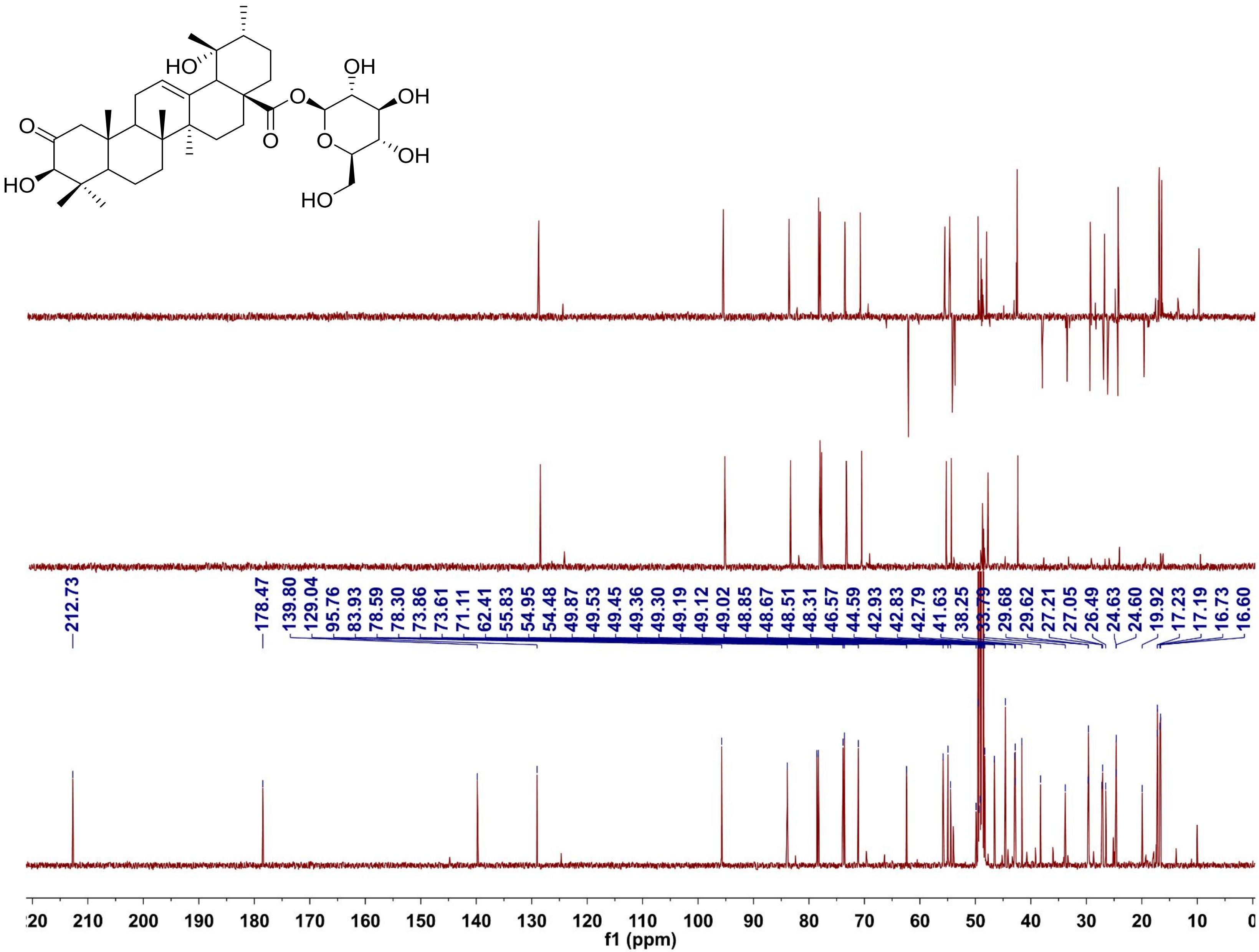

S11-2 ^{13}C NMR spectrum of compound **11** in CD_3OD (125 MHz)

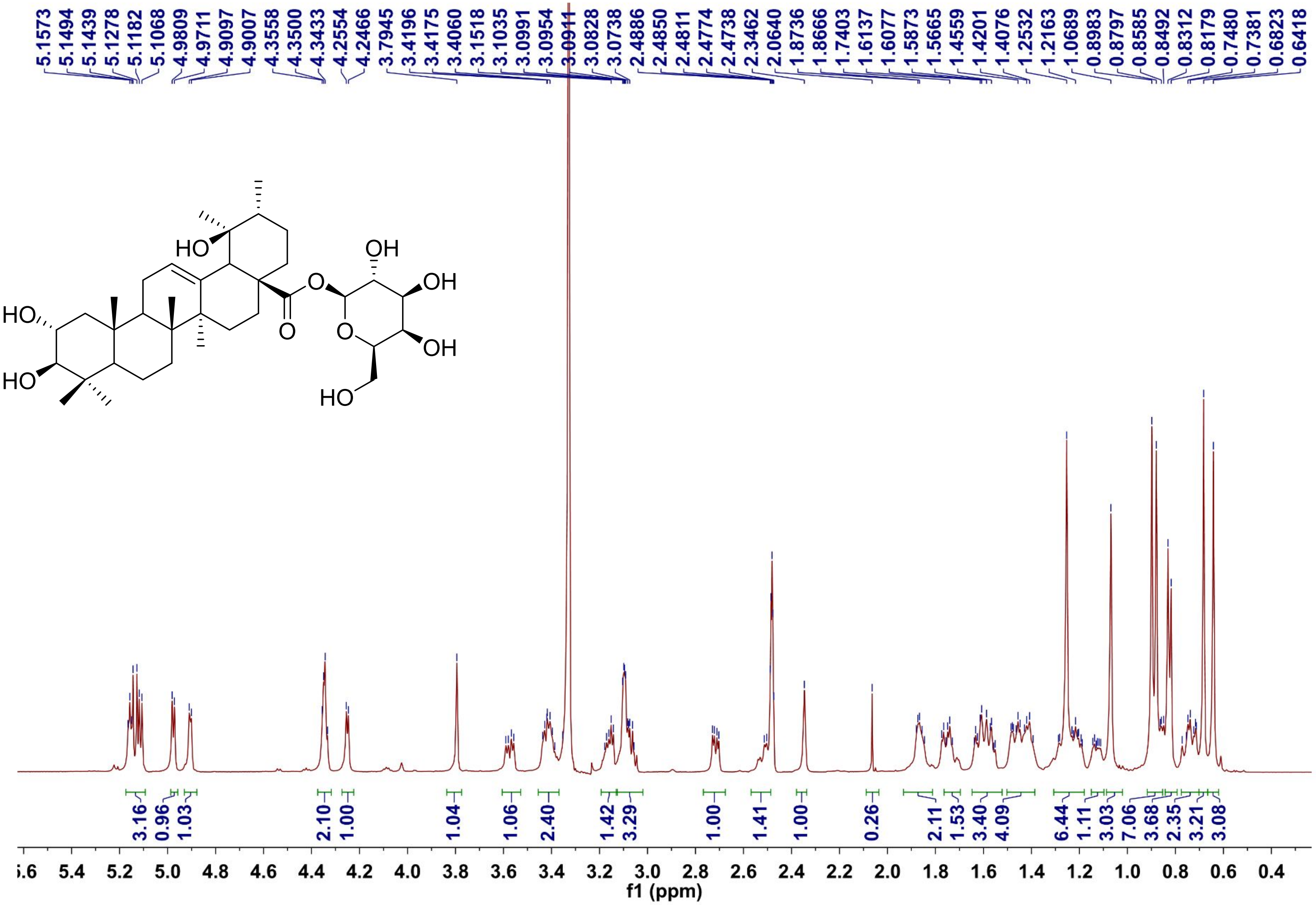

S12-1 ^{1}H NMR spectrum of compound **12** in DMSO-d_6 (500 MHz)

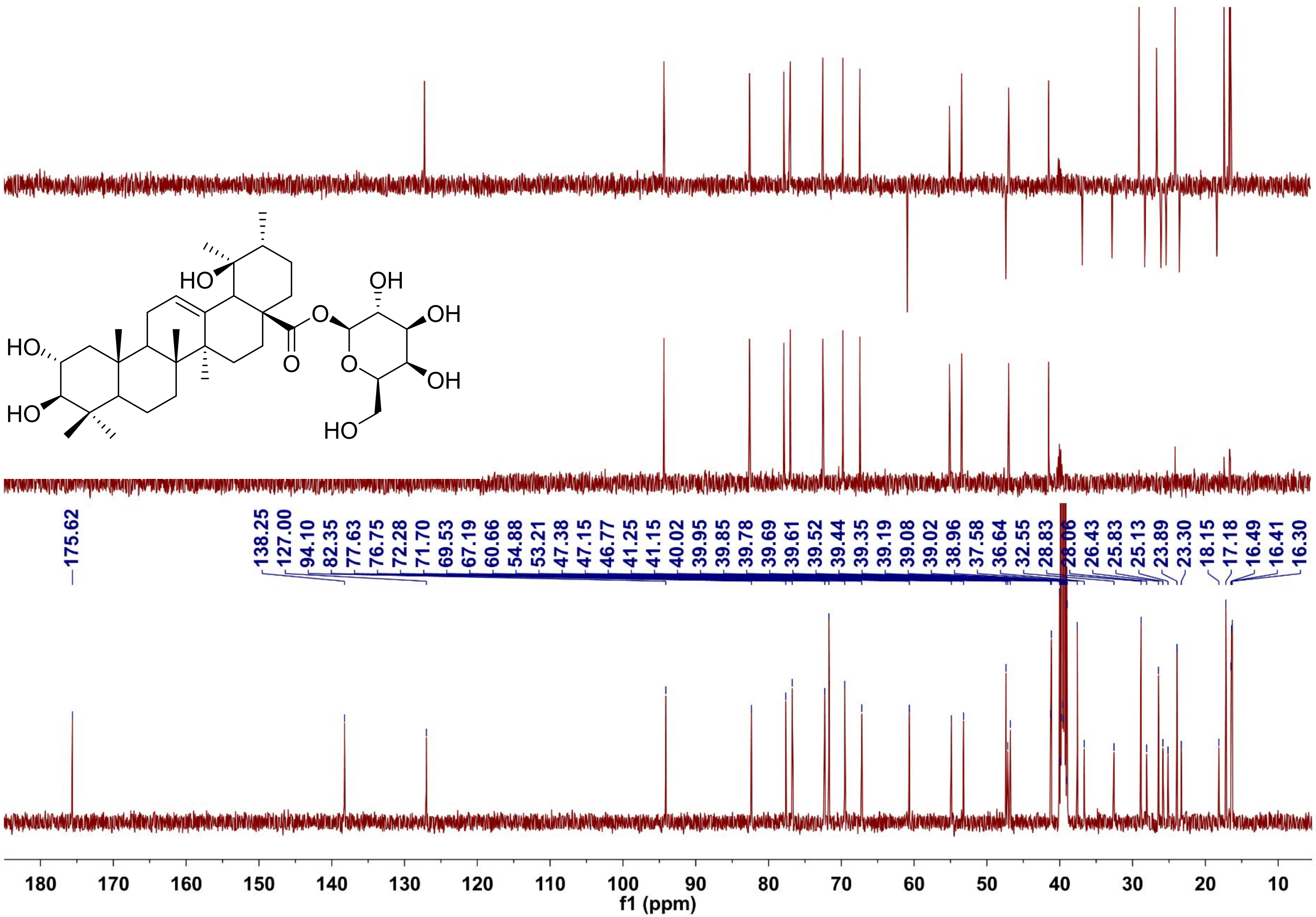

S12-2 ^{13}C NMR spectrum of compound **12** in DMSO-d_6 (125 MHz)

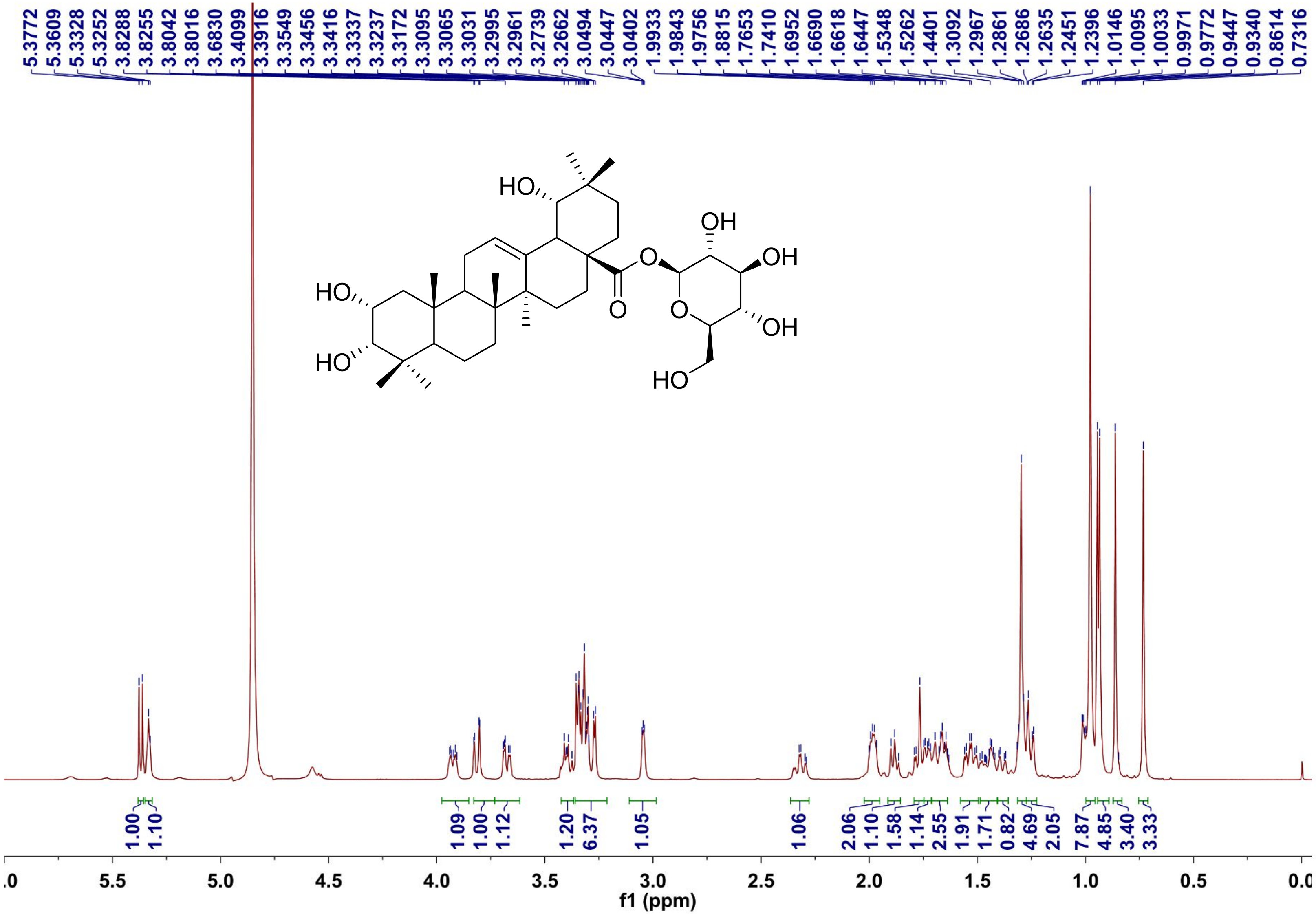

S13-1 ^1H NMR spectrum of compound **13** in CD_3OD (500 MHz)

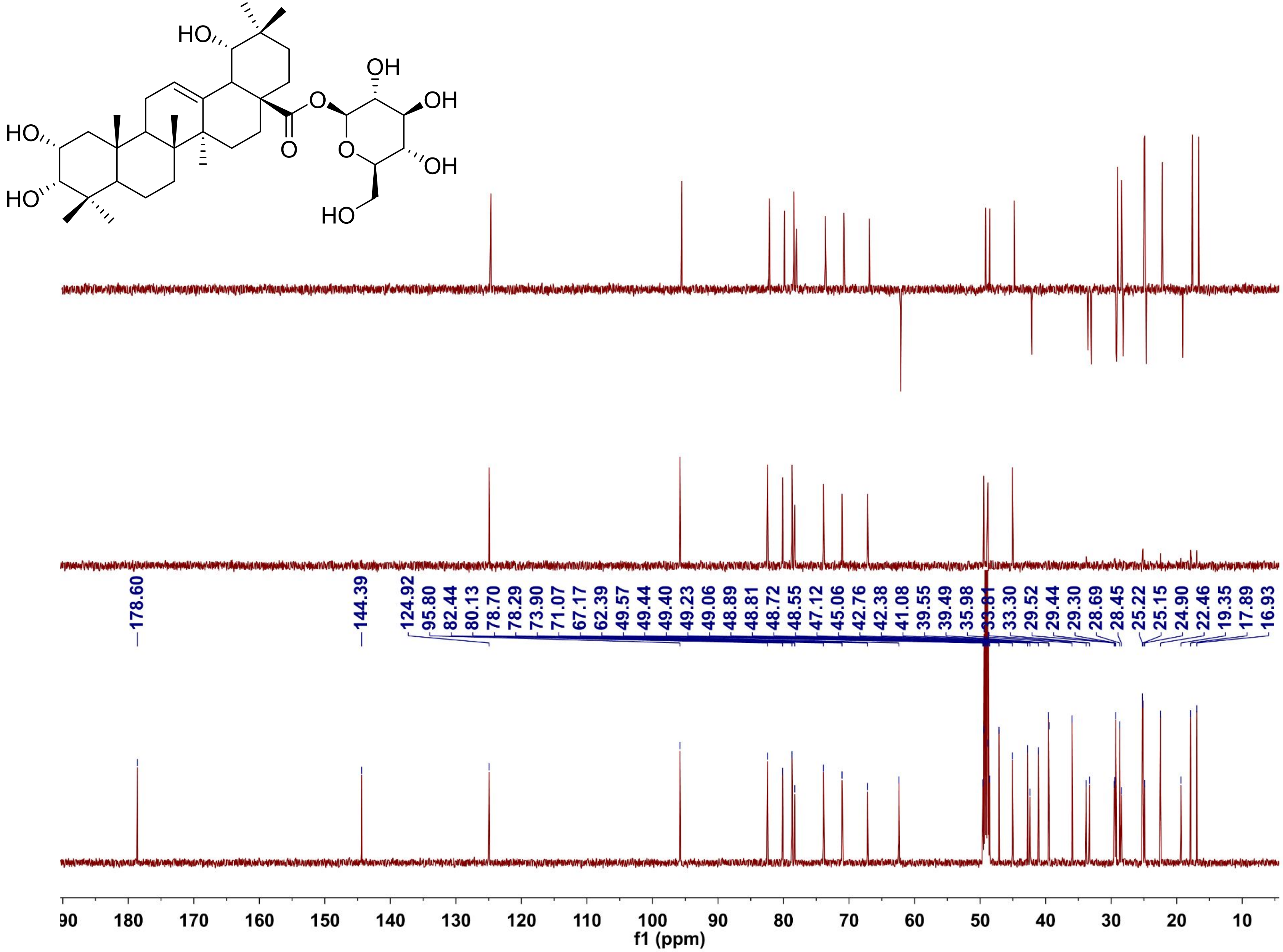

S13-2 ^{13}C NMR spectrum of compound **13** in CD_3OD (125 MHz)

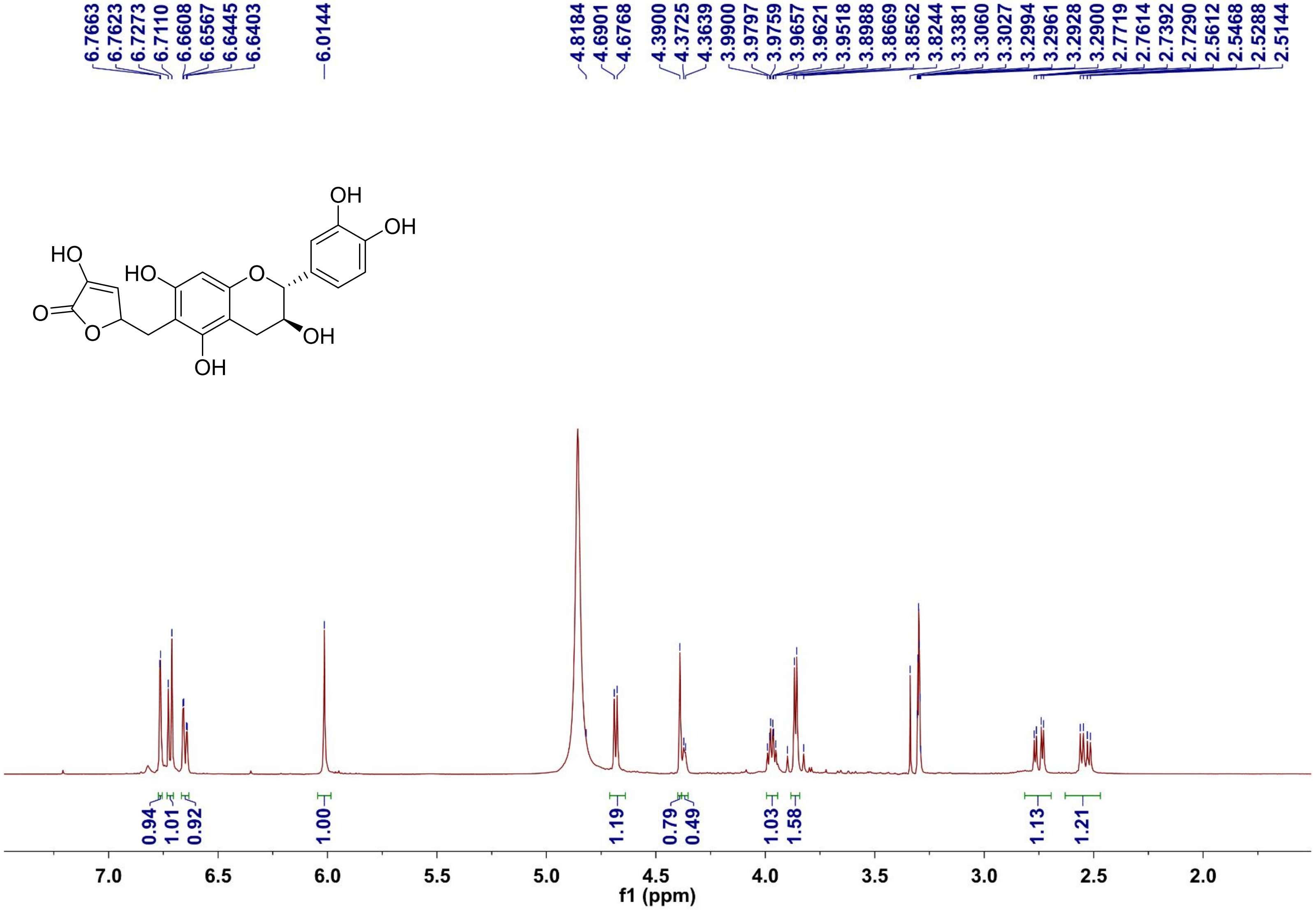

S14-1 [1] H NMR spectrum of compound **14** in CD_3OD (500 MHz)

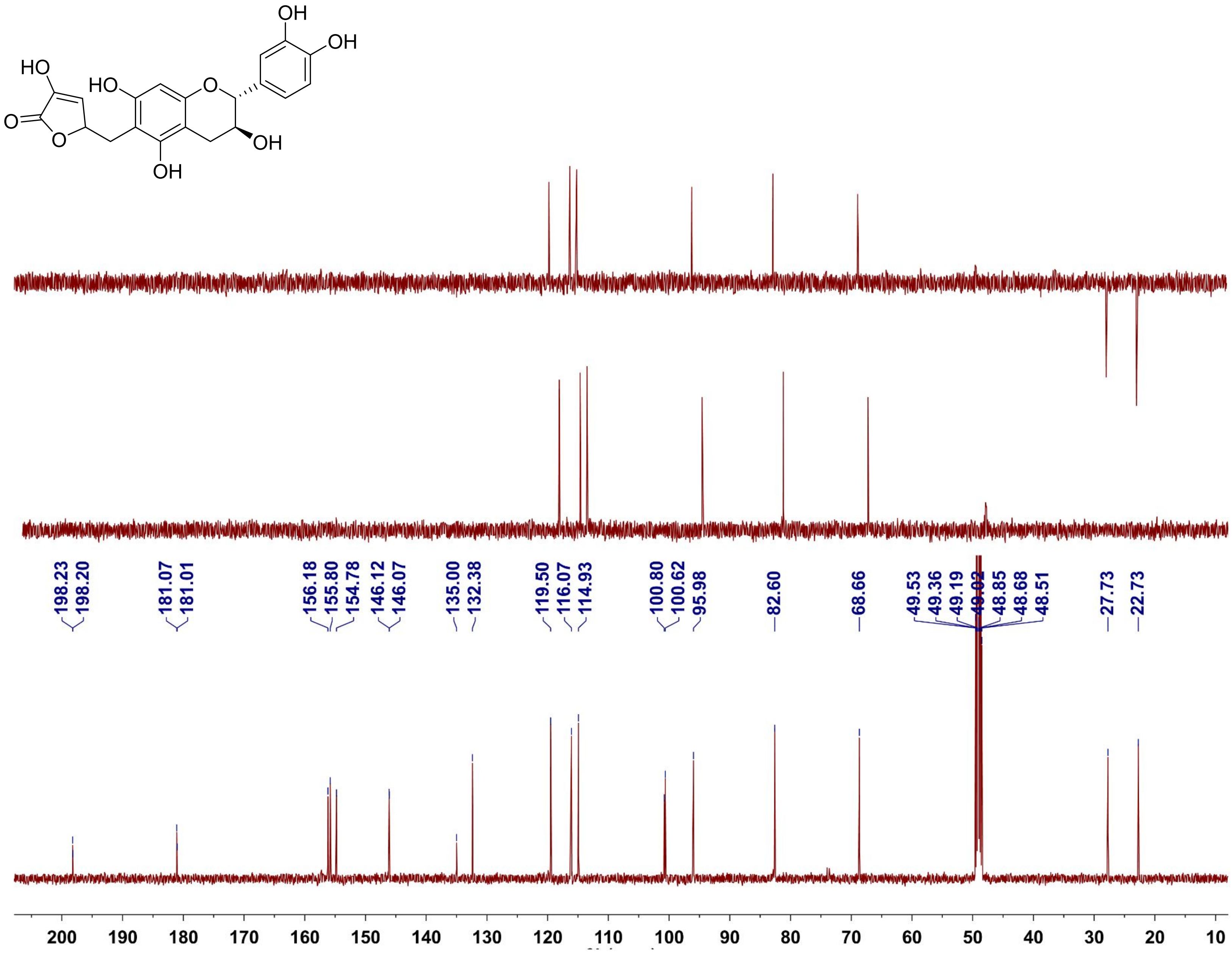

S14-2 ^{13}C NMR spectrum of compound **14** in CD_3OD (125 MHz)

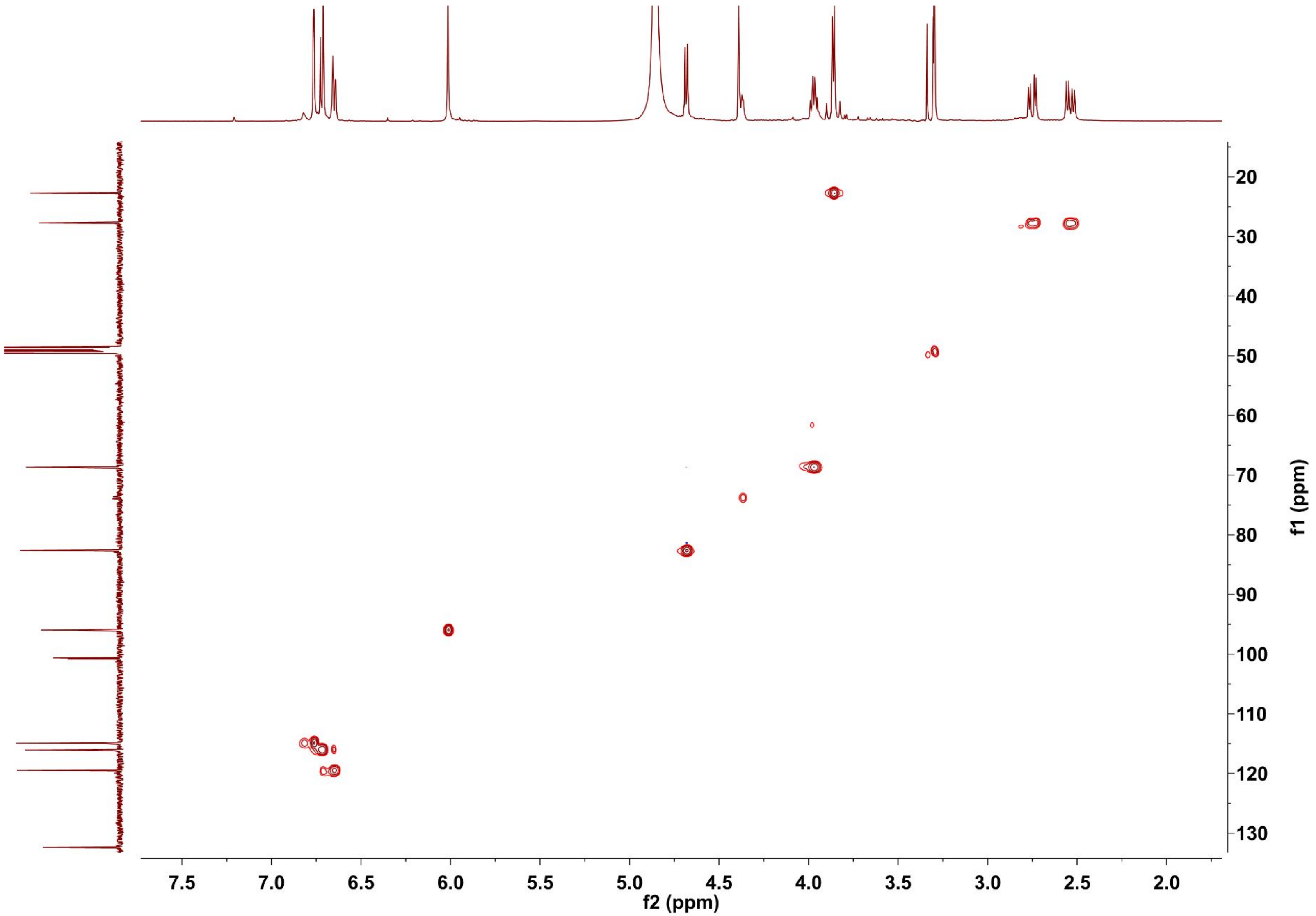

S14-3 HSQC spectrum of compound **14** in CD_3OD (500 MHz)

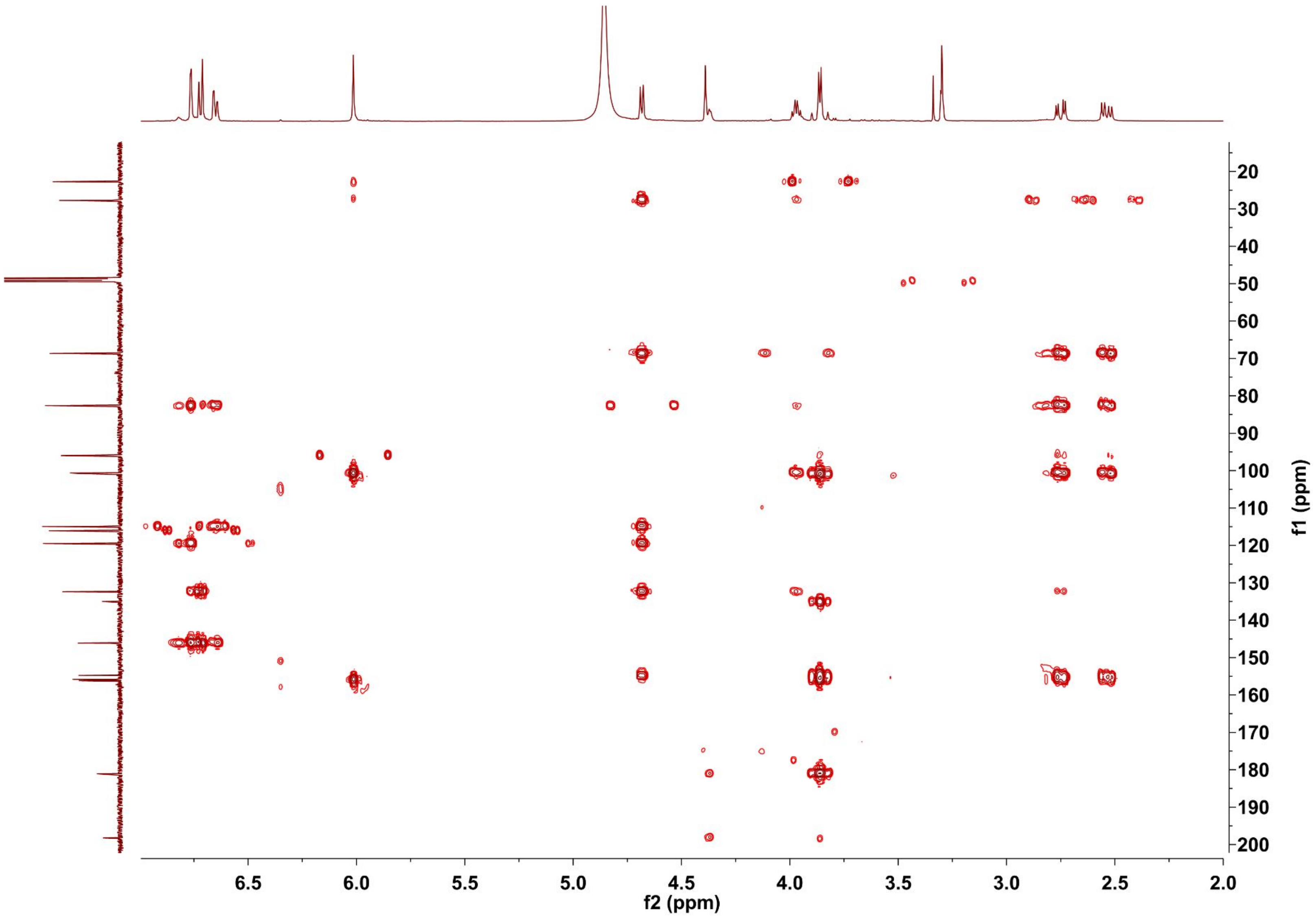

S14-4 HMBC spectrum of compound **14** in CD_3OD (500 MHz)

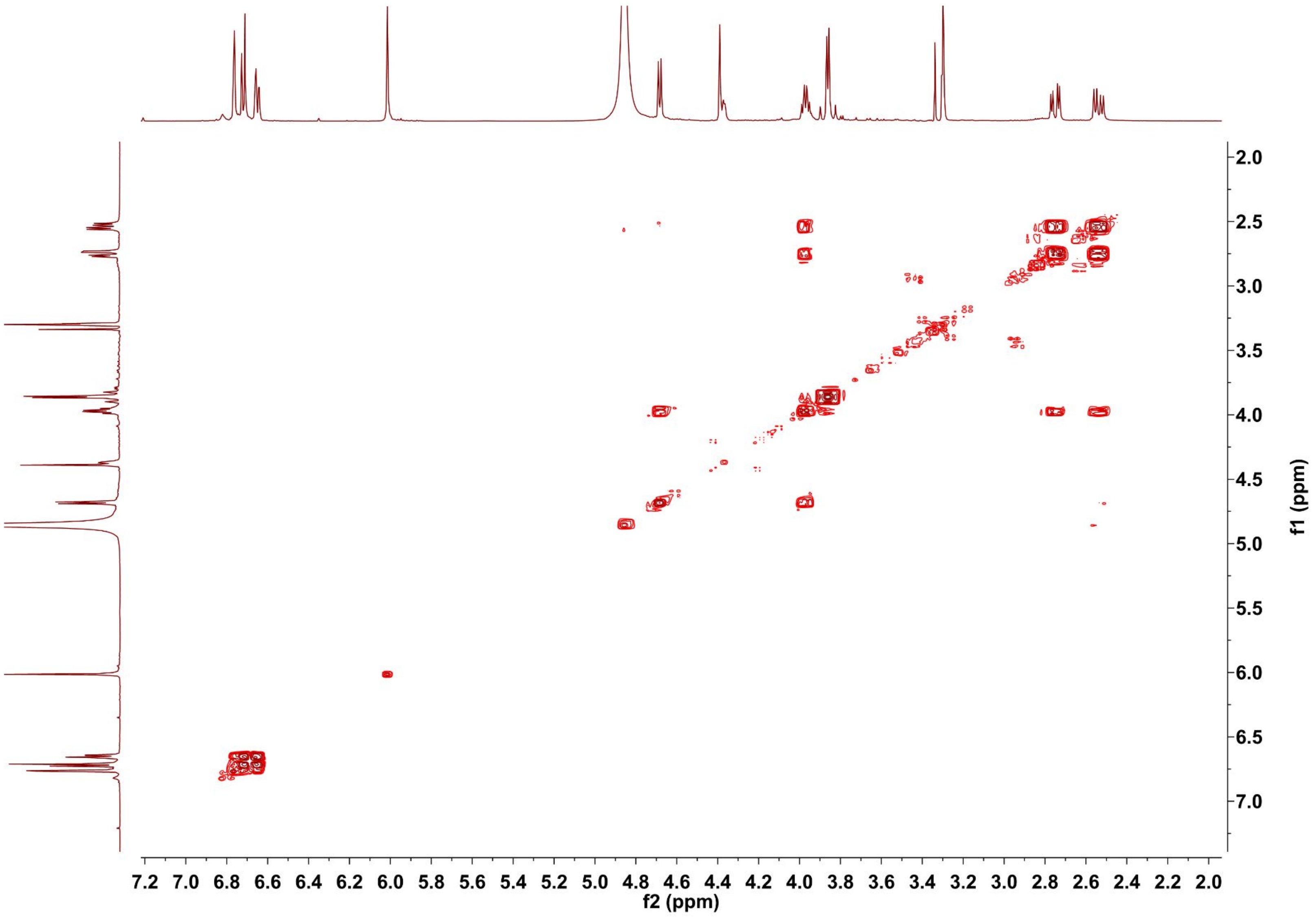

S14-5 ^{1}H-^{1}H COSY spectrum of compound **14** in CD_3OD (500 MHz)

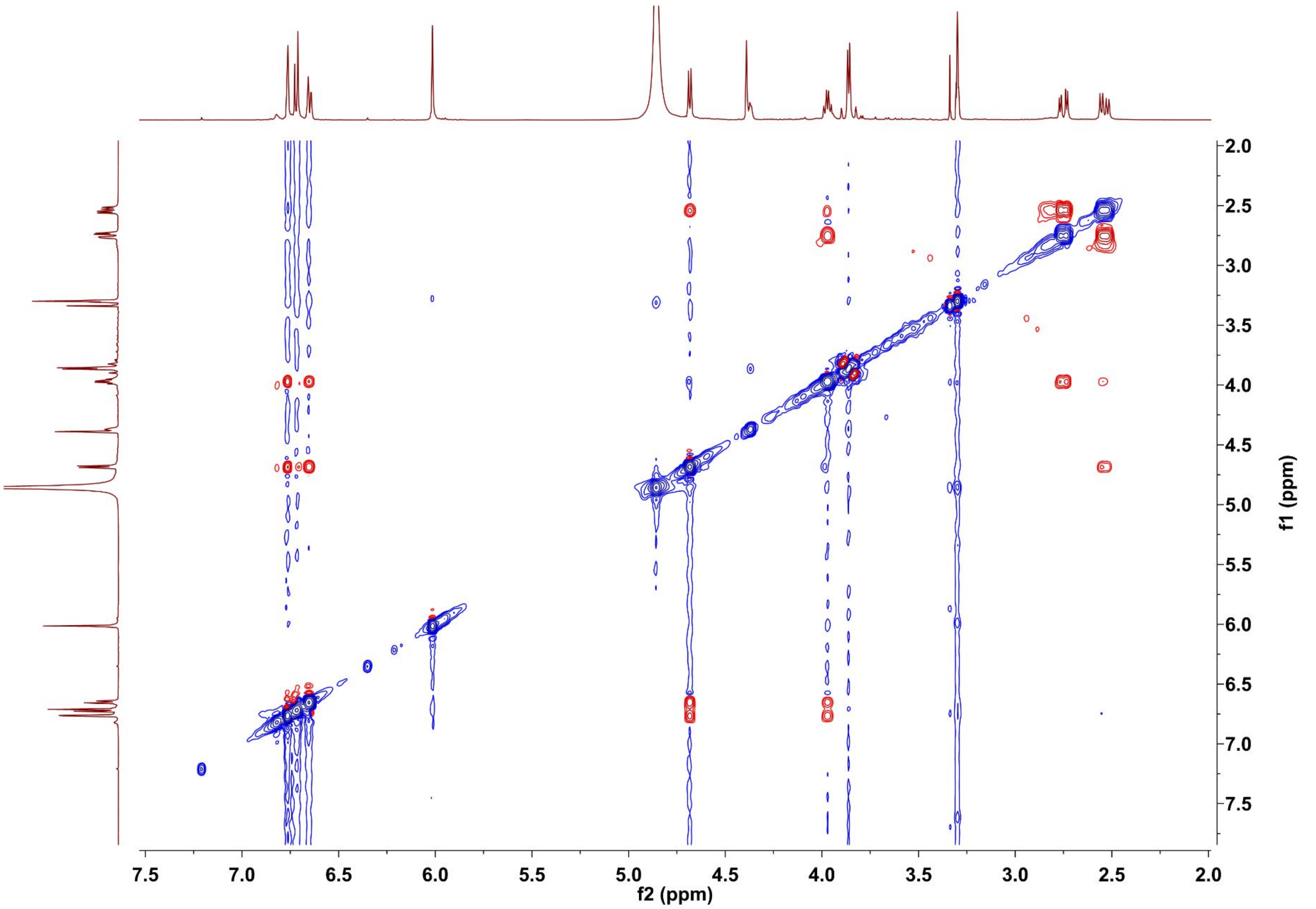

S14-6 ROESY spectrum of compound **14** in CD_3OD (500 MHz)

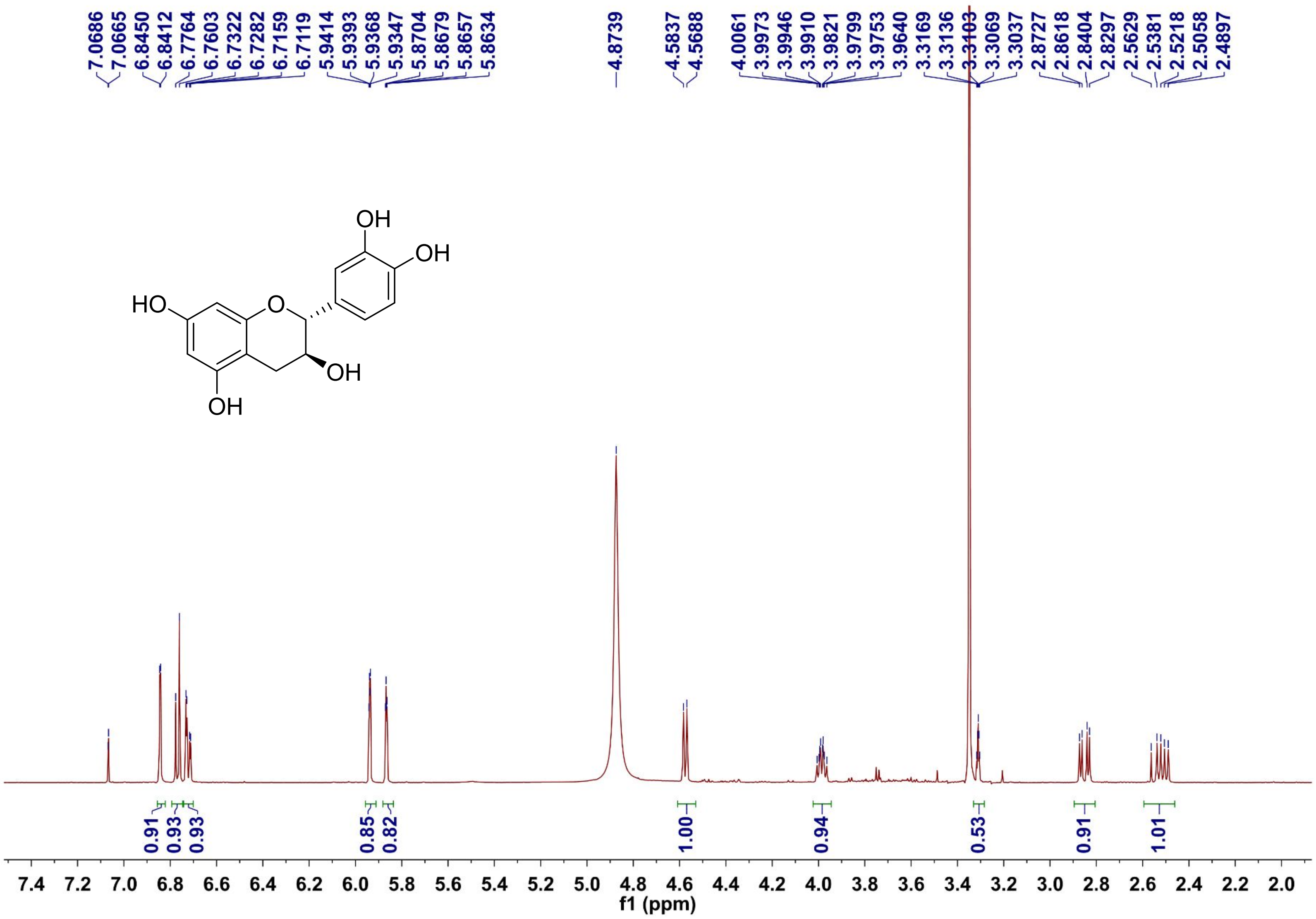
7.0686
7.0665
6.8450
6.8412
6.7764
6.7603
6.7322
6.7282
6.7159
6.7119
5.9414
5.9393
5.9368
5.9347
5.8704
5.8679
5.8657
5.8634
4.8739
4.5837
4.5688
4.0061
3.9973
3.9946
3.9910
3.9821
3.9799
3.9753
3.9640
3.3169
3.3136
3.3103
3.3069
3.3037
2.8727
2.8618
2.8404
2.8297
2.5629
2.5381
2.5218
2.5058
2.4897
HO
OH
OH
OH
OH
0.91
0.93
0.93
0.85
0.82
1.00
0.94
0.53
0.91
1.01
7.4 7.2 7.0 6.8 6.6 6.4 6.2 6.0 5.8 5.6 5.4 5.2 5.0 4.8 4.6 4.4 4.2 4.0 3.8 3.6 3.4 3.2 3.0 2.8 2.6 2.4 2.2 2.0
f1 (ppm)

S15-1 ^{1}H NMR spectrum of compound **15** in CD_3OD (500 MHz)

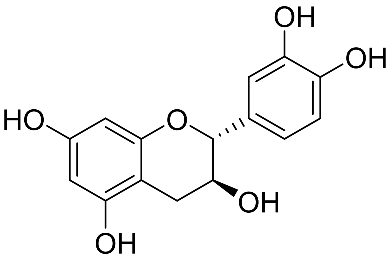

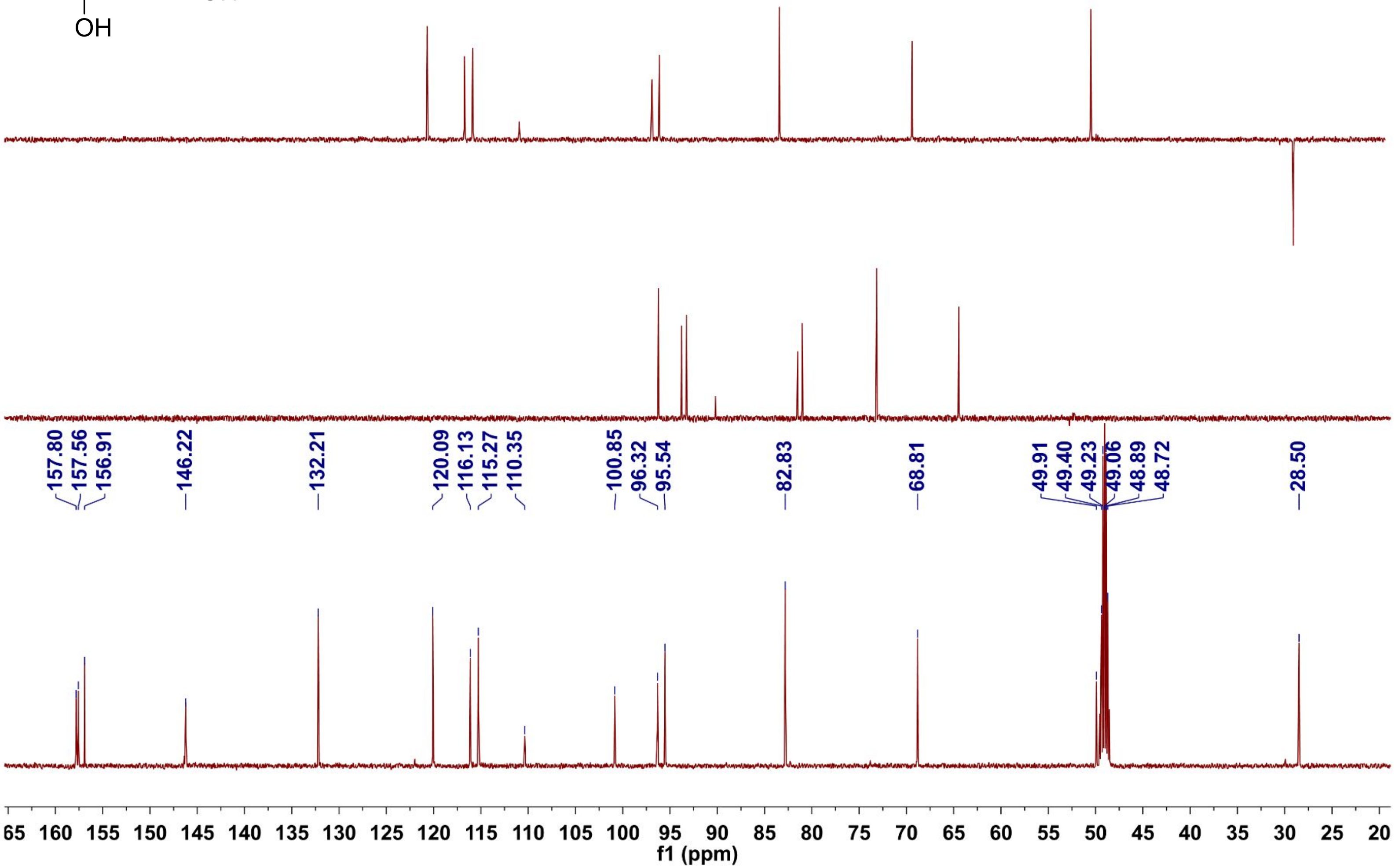

S15-2 ^{13}C NMR spectrum of compound **15** in CD_3OD (125 MHz)

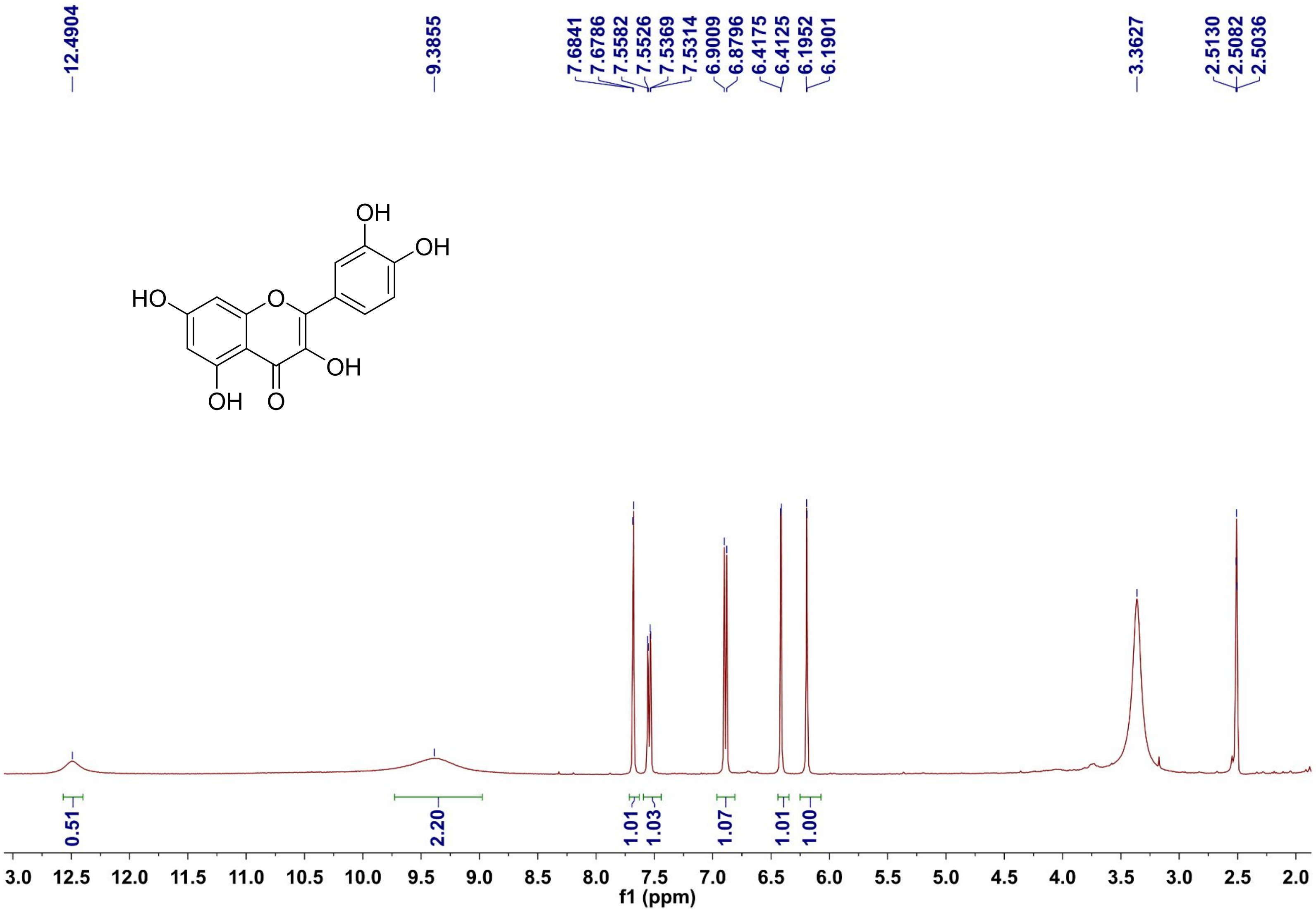

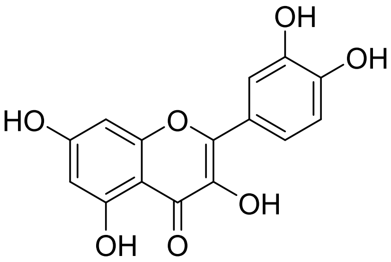

S16-1 ^{1}H NMR spectrum of compound **16** in CD_3OD (400 MHz)

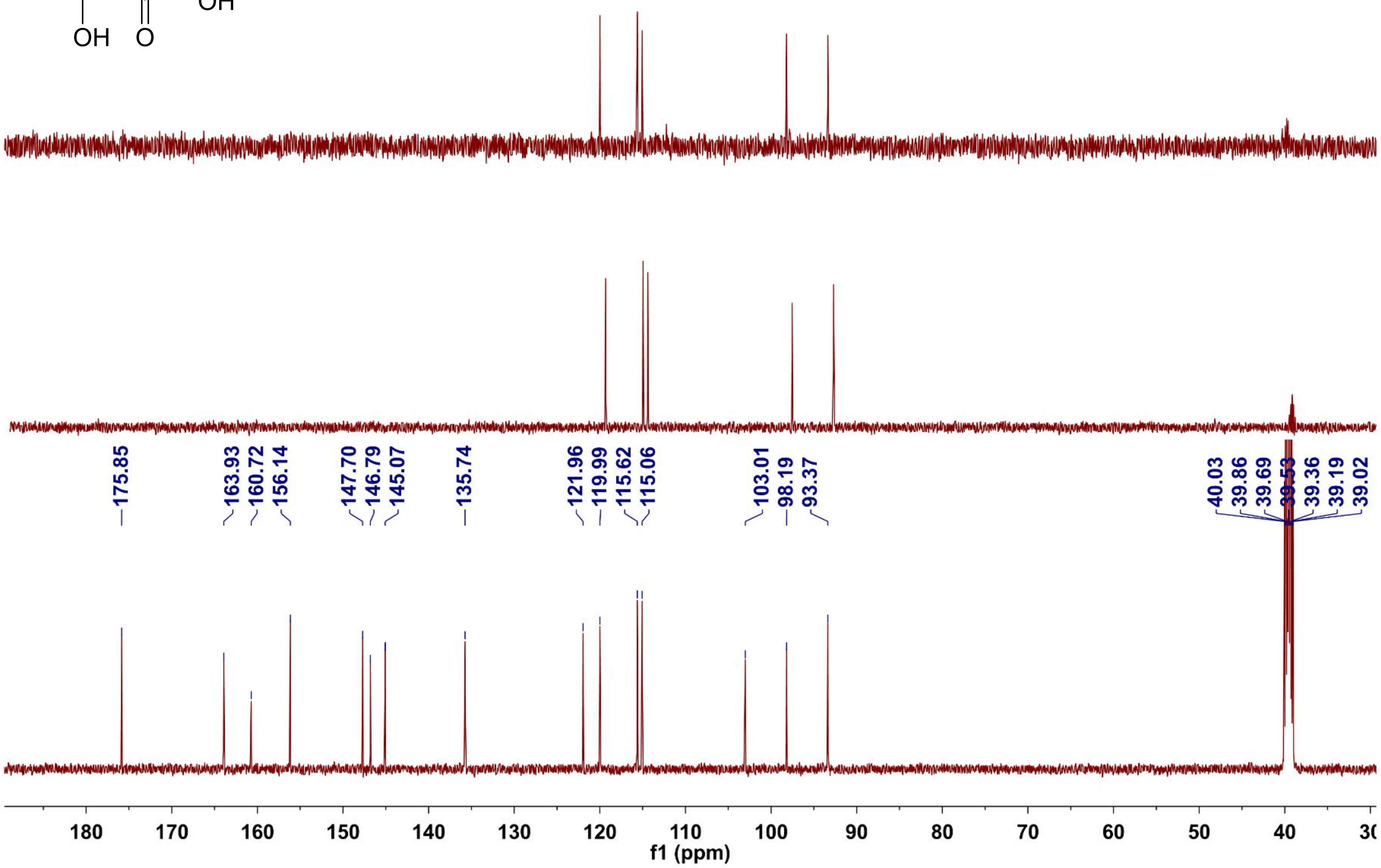

S16-2 ^{13}C NMR spectrum of compound **16** in CD_3OD (125 MHz)

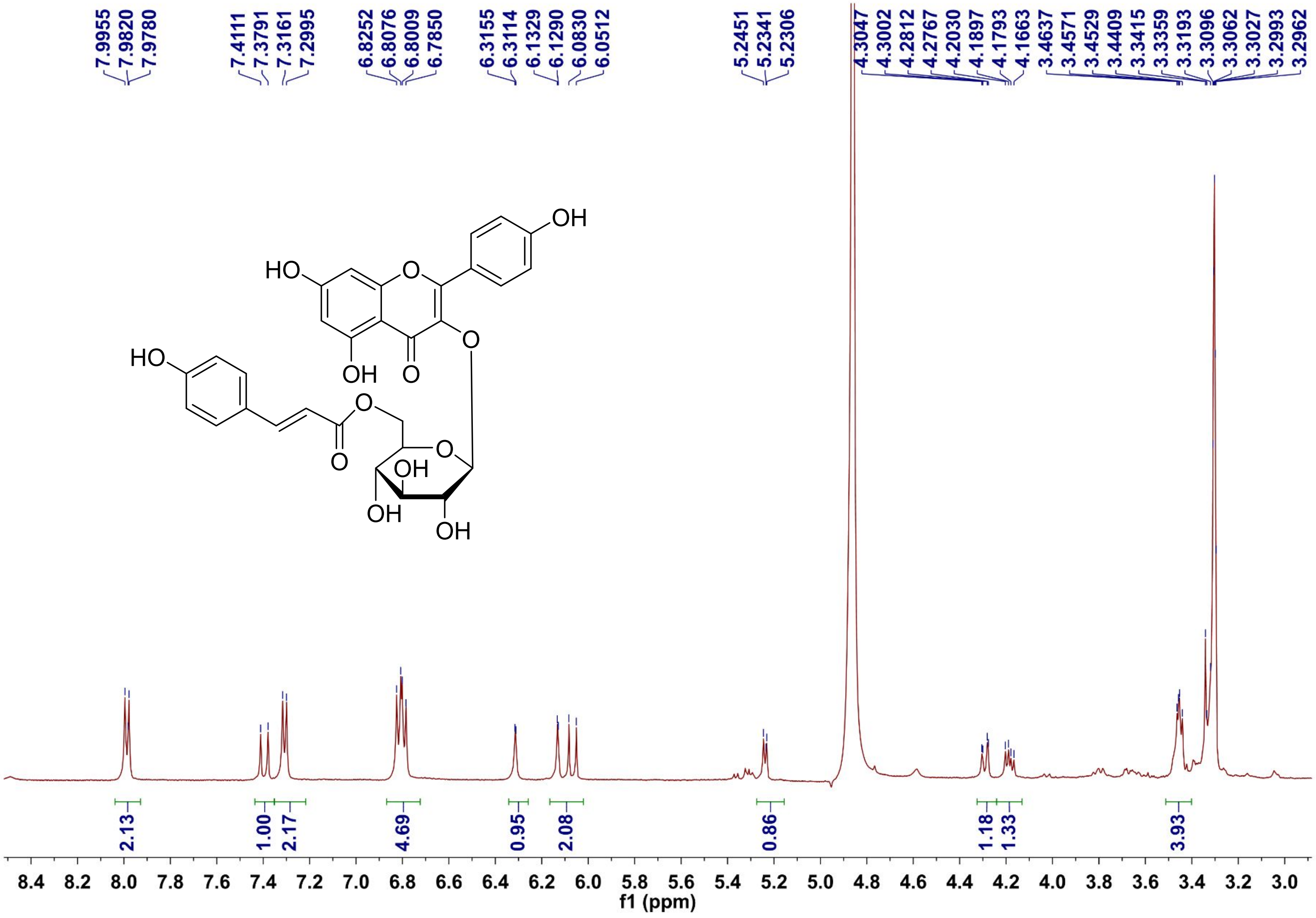

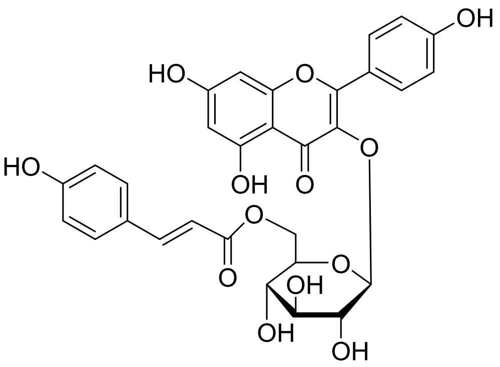

S17-1 ^{1}H NMR spectrum of compound **17** in CD_3OD (500 MHz)

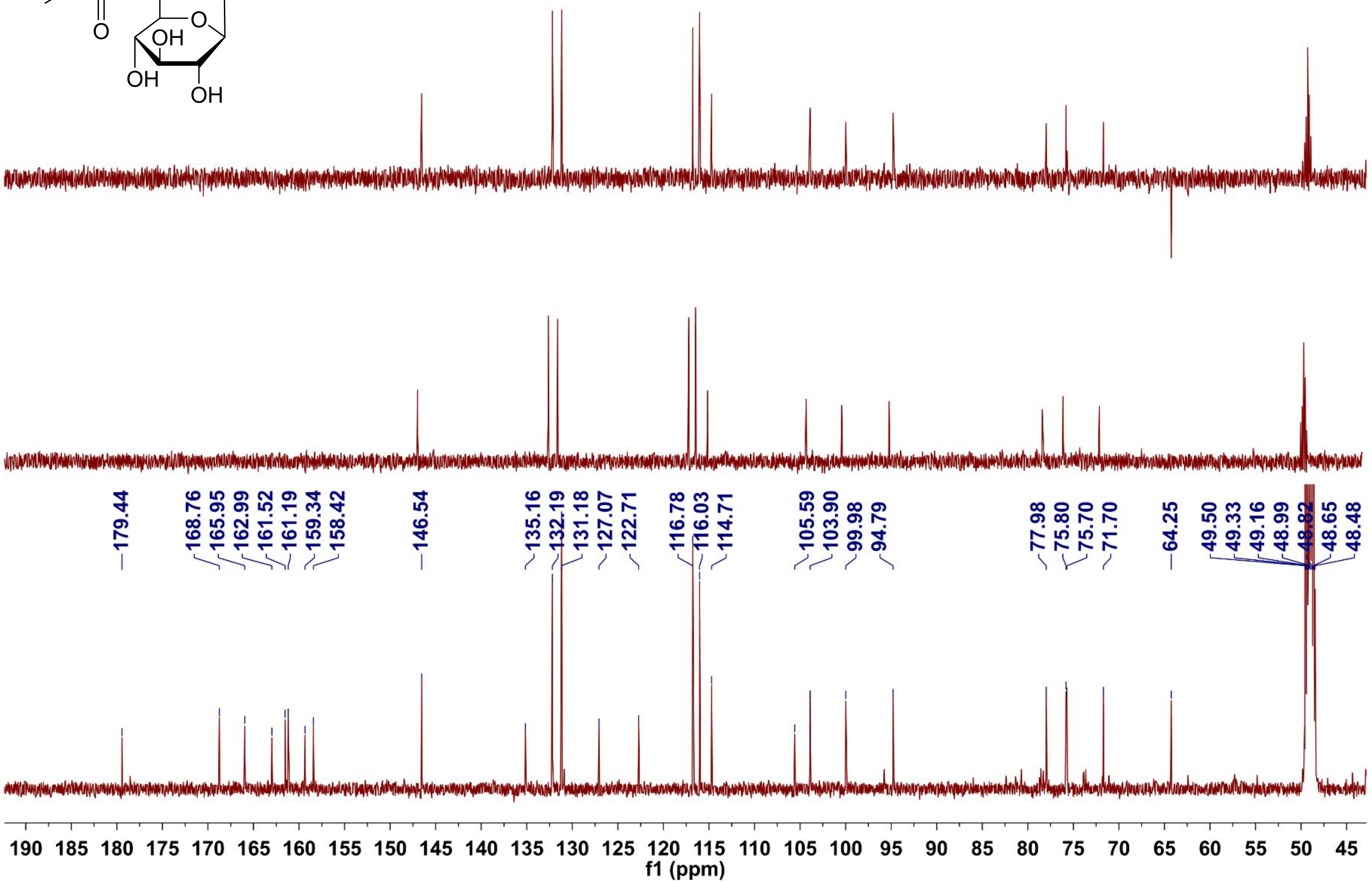

S17-2 ¹³C NMR spectrum of compound **17** in CD_3OD (125 MHz)

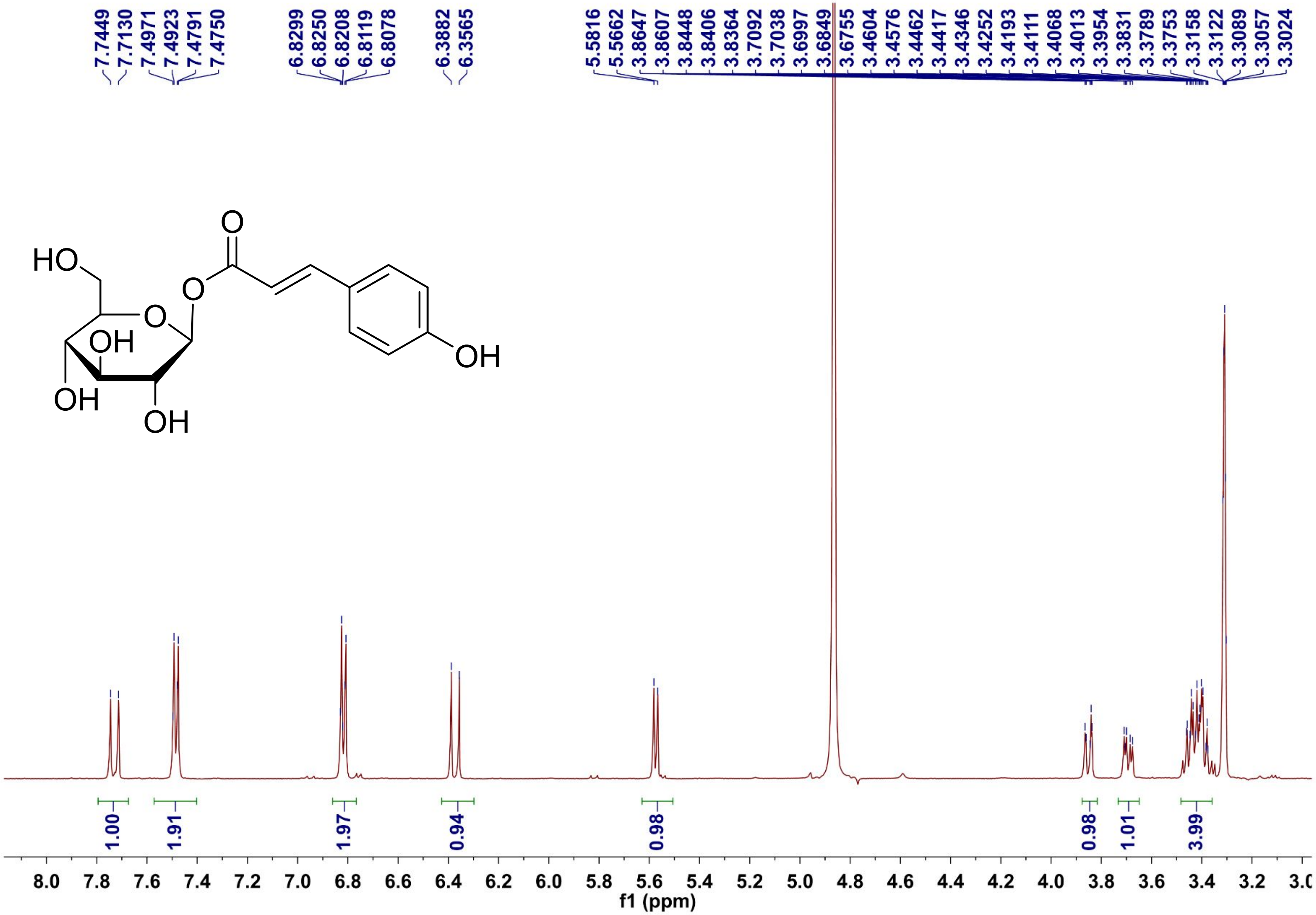

S18-1 [1] H NMR spectrum of compound **18** in CD_3OD (500 MHz)

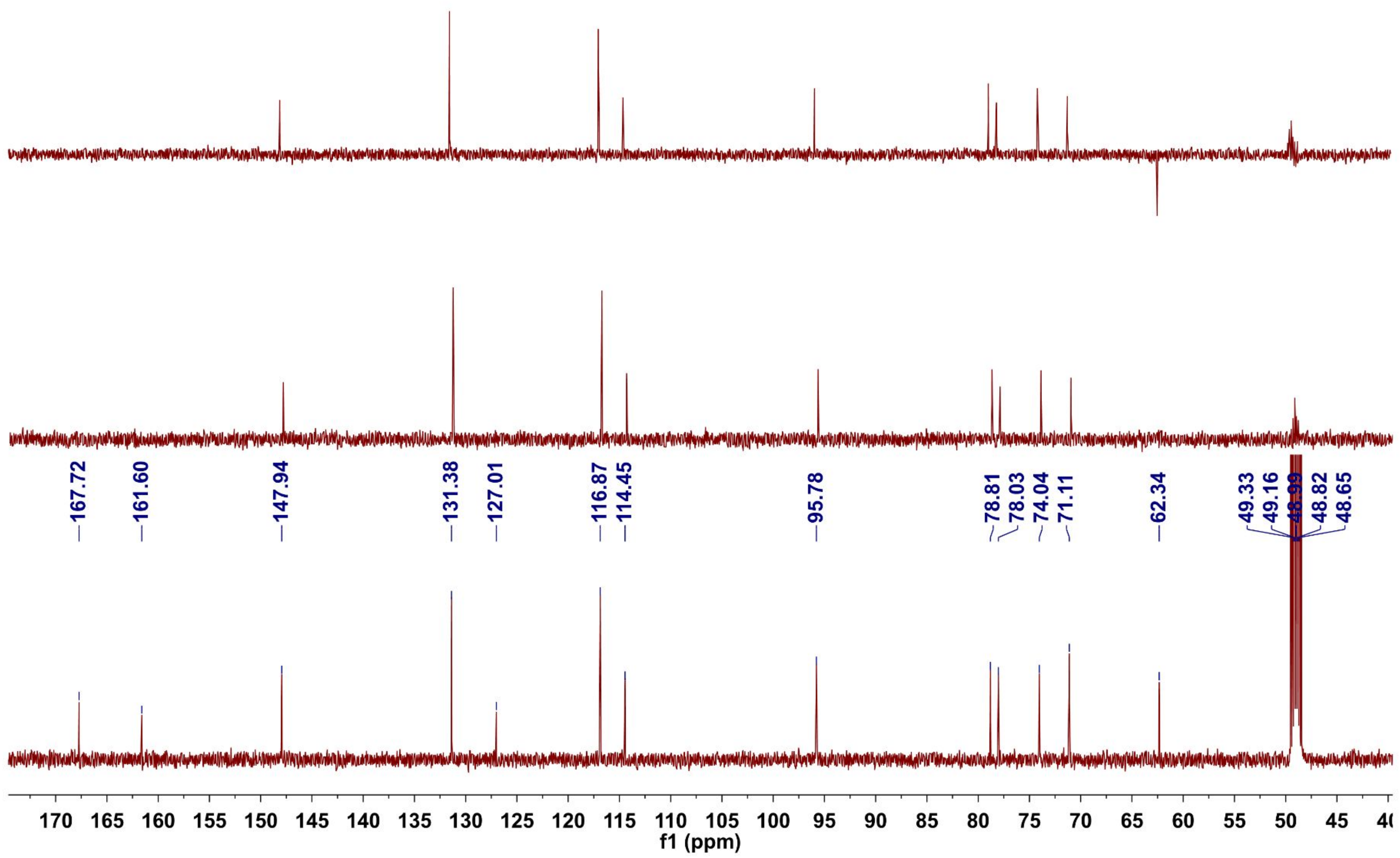

S18-2 ^{13}C NMR spectrum of compound **18** in CD_3OD (125 MHz)

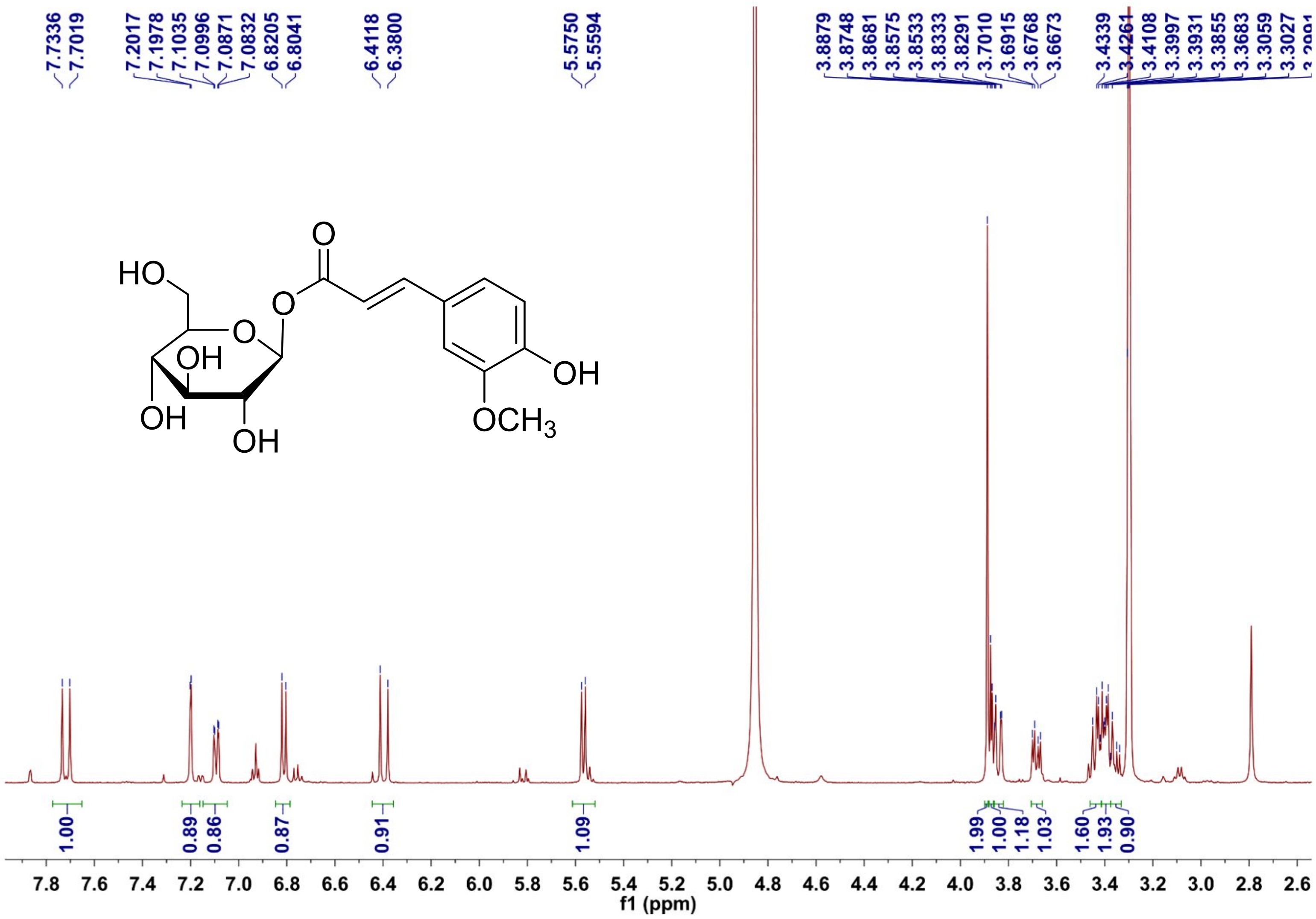

ɔf compound **19** in CD_3OD (500 MHz)

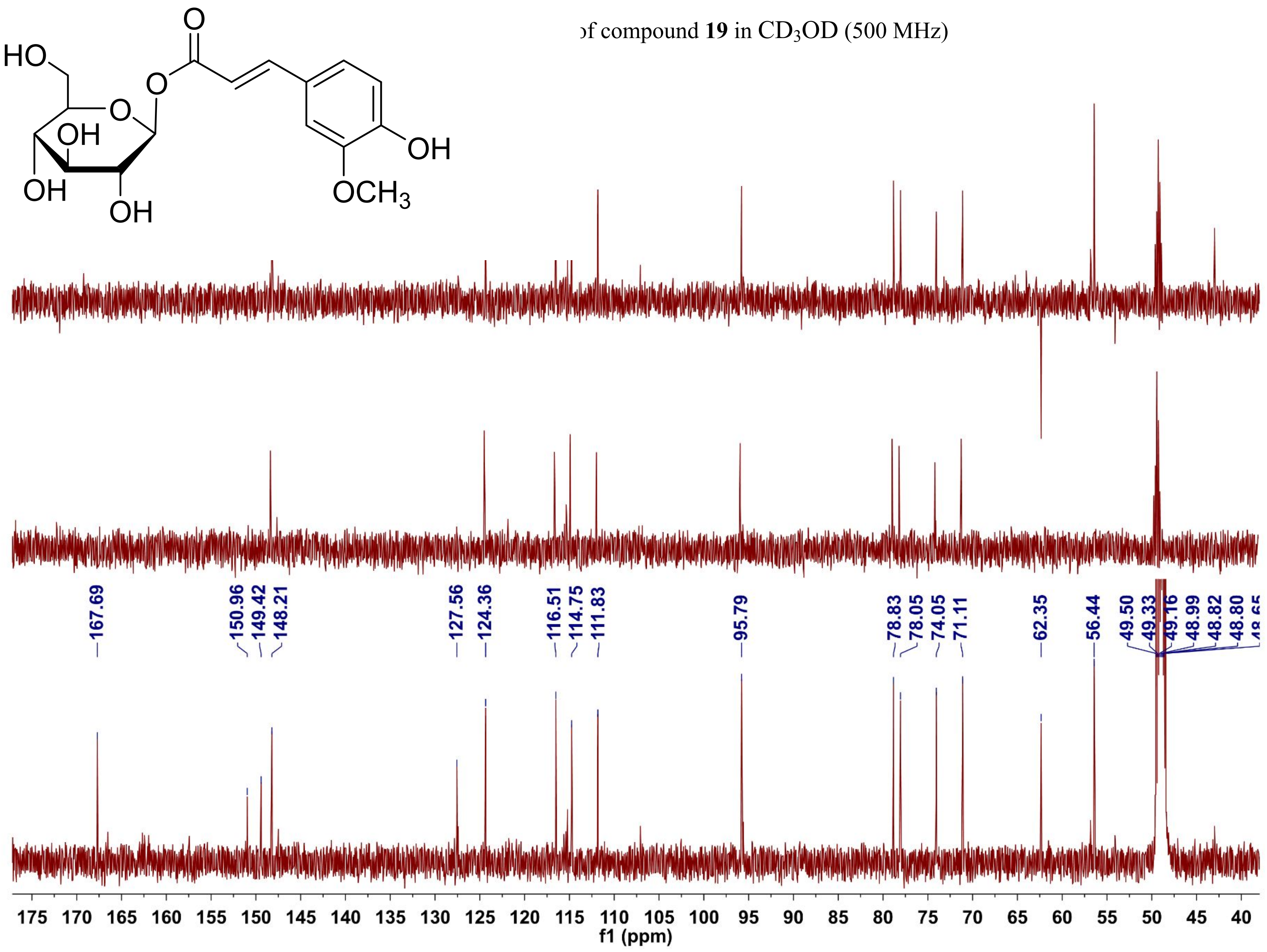

S19-2 ^{13}C NMR spectrum of compound **19** in CD_3OD (125 MHz)

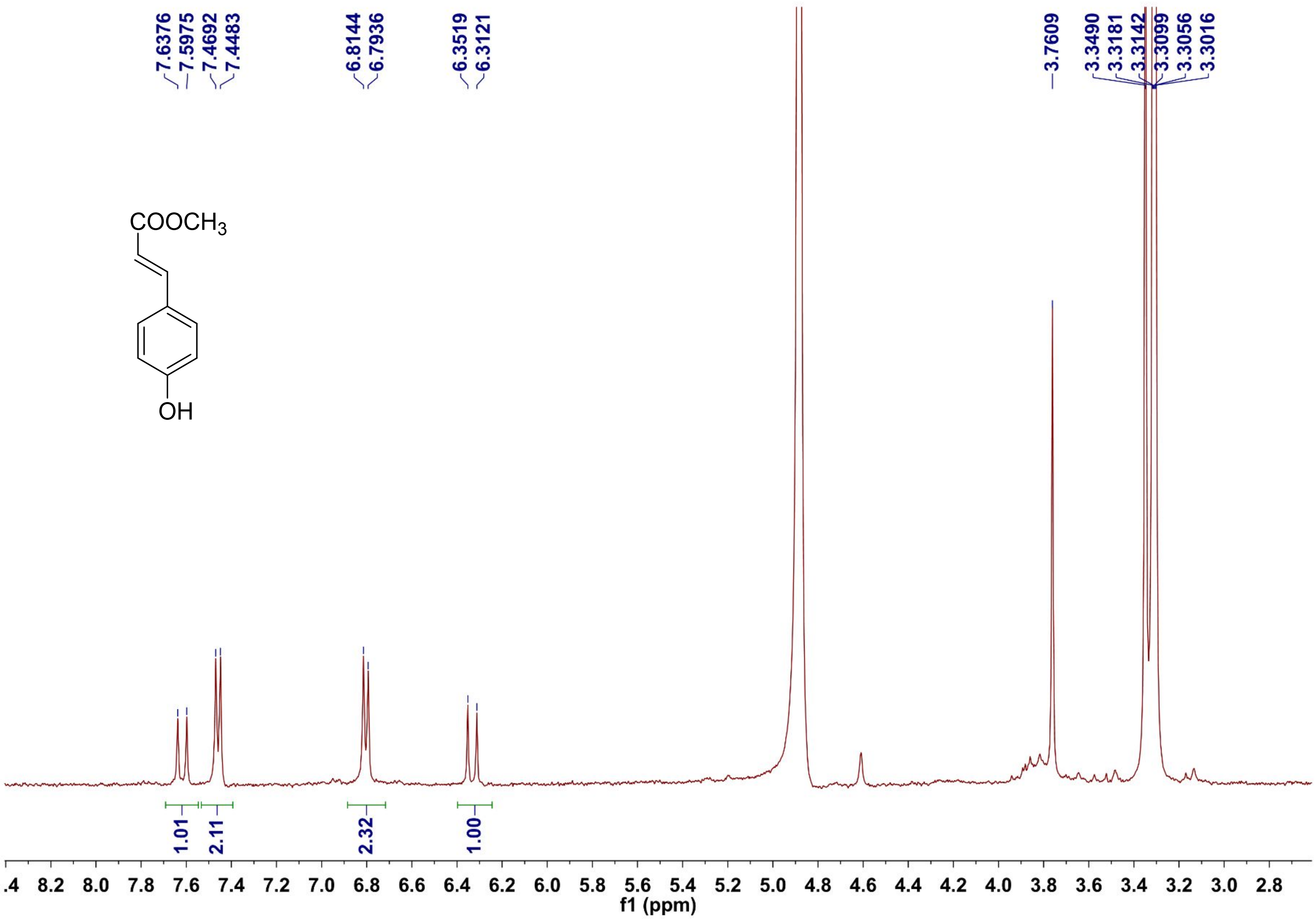

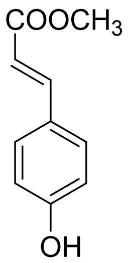

S20-1 ^{1}H NMR spectrum of compound **20** in CD_3OD (600 MHz)

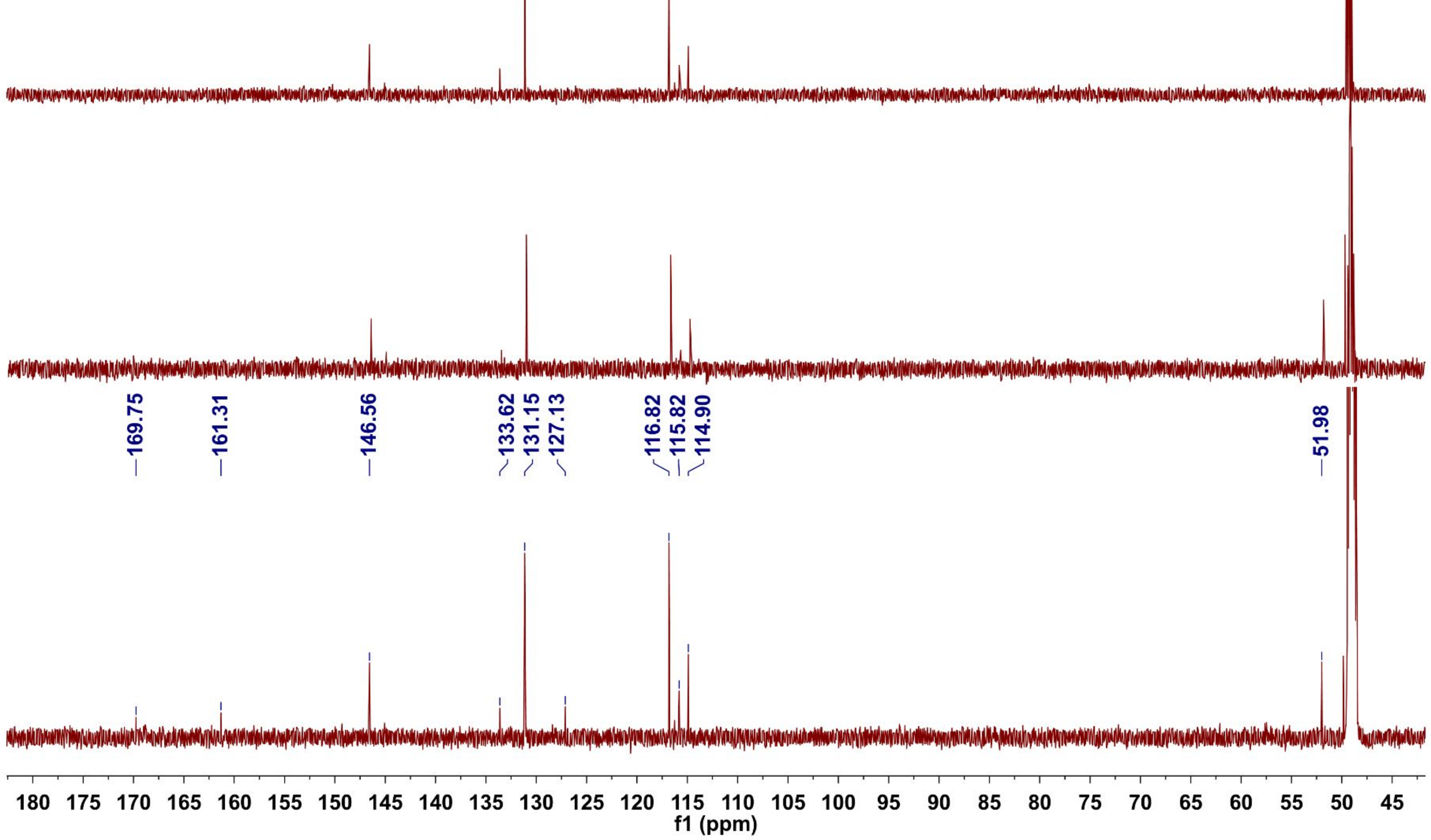

S20-2 ^{13}C NMR spectrum of compound **20** in CD_3OD (150 MHz)

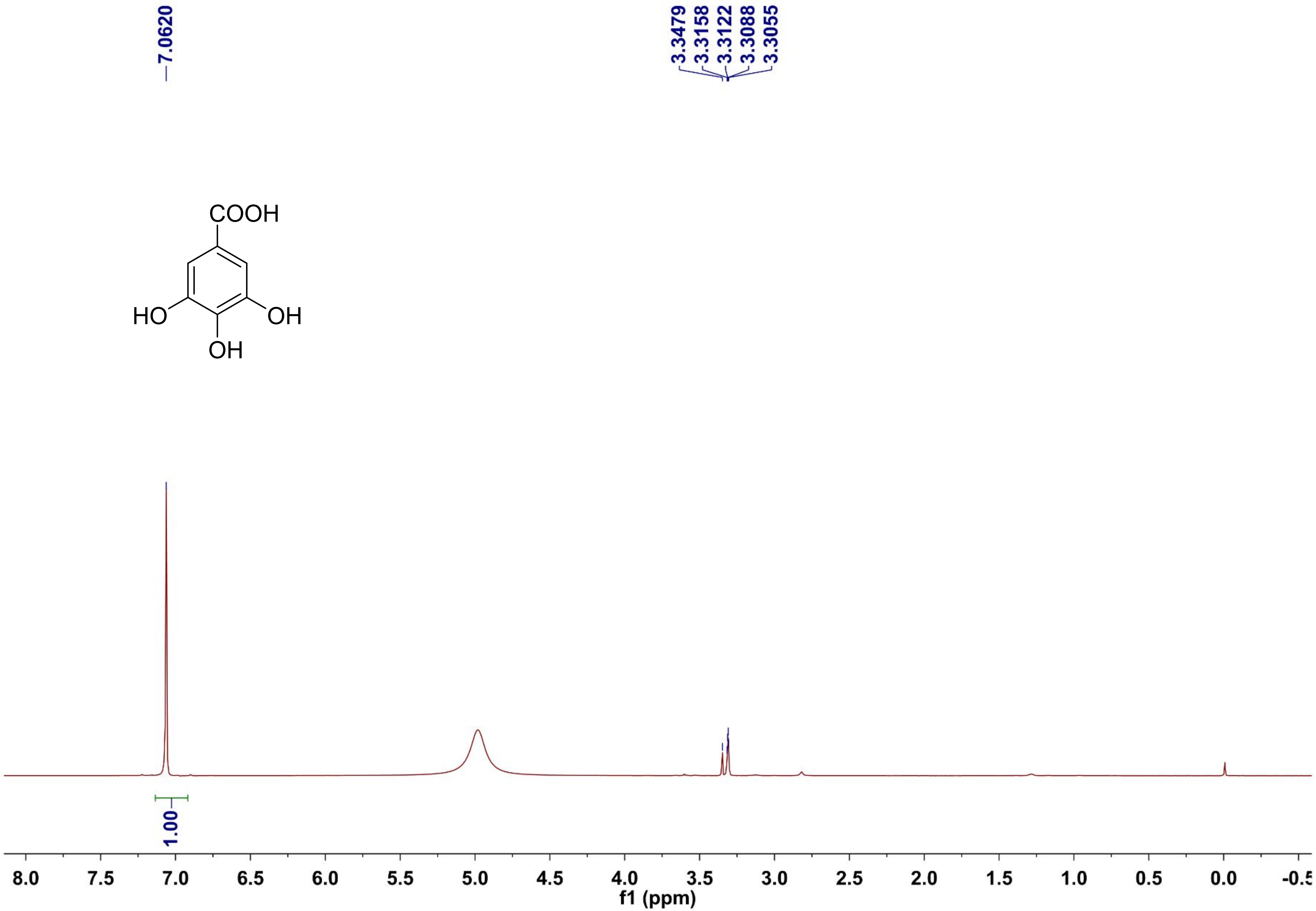

S21-1 [1] H NMR spectrum of compound **21** in CD_3OD (500 MHz)

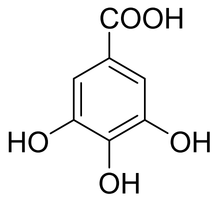

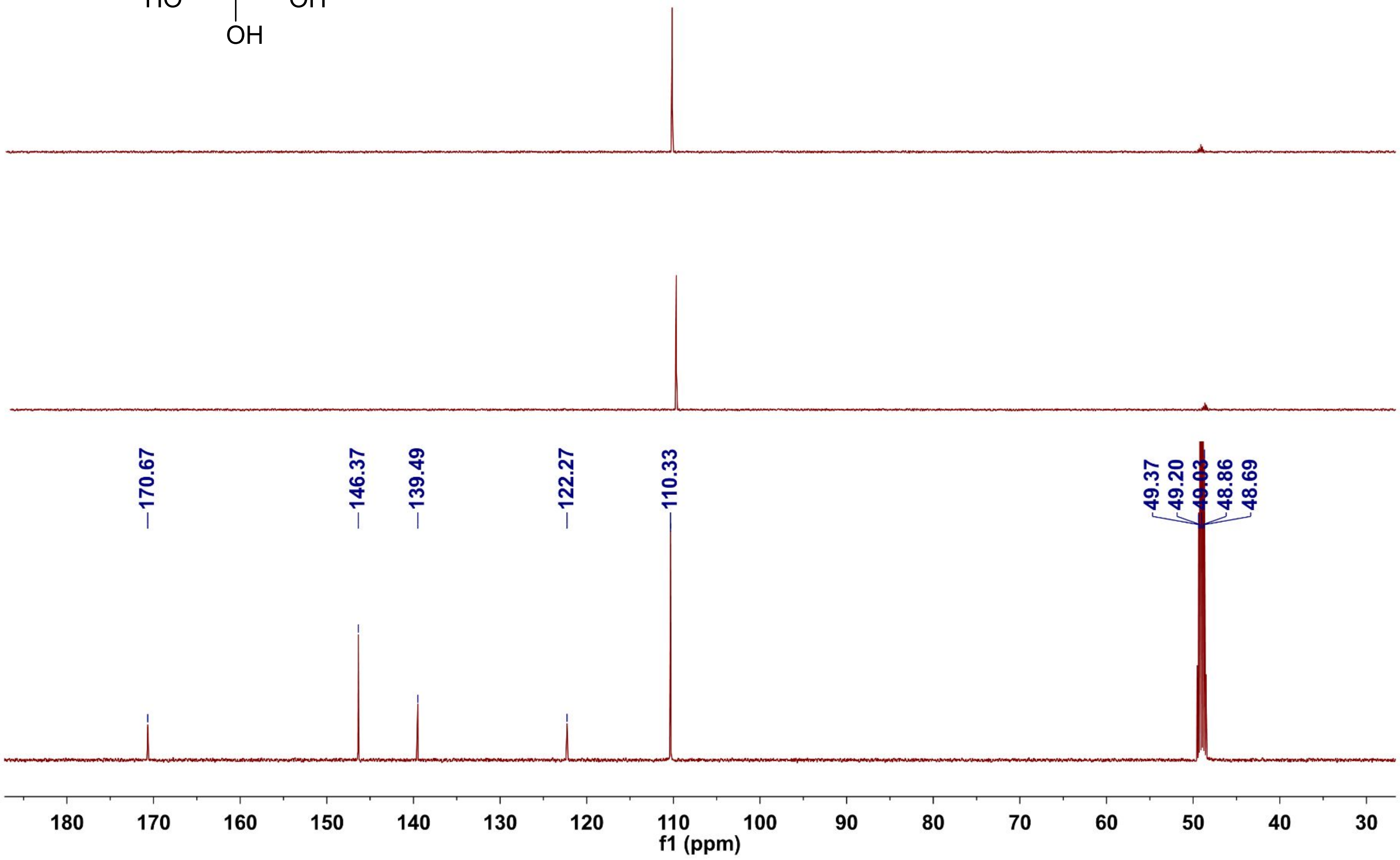

S21-2 ^{13}C NMR spectrum of compound **21** in CD_3OD (125 MHz)

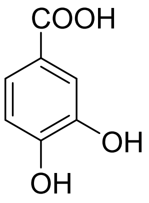

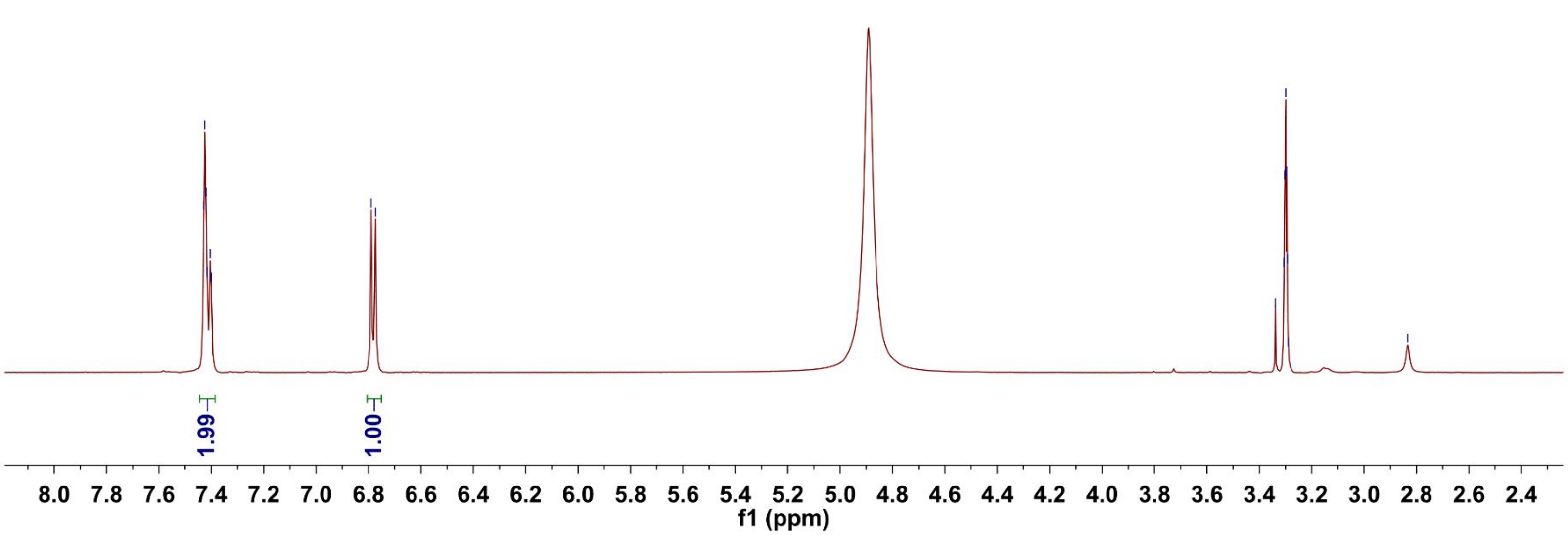

S22-1 [1] H NMR spectrum of compound **22** in CD_3OD (500 MHz)

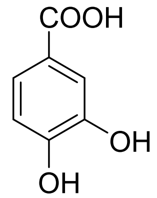

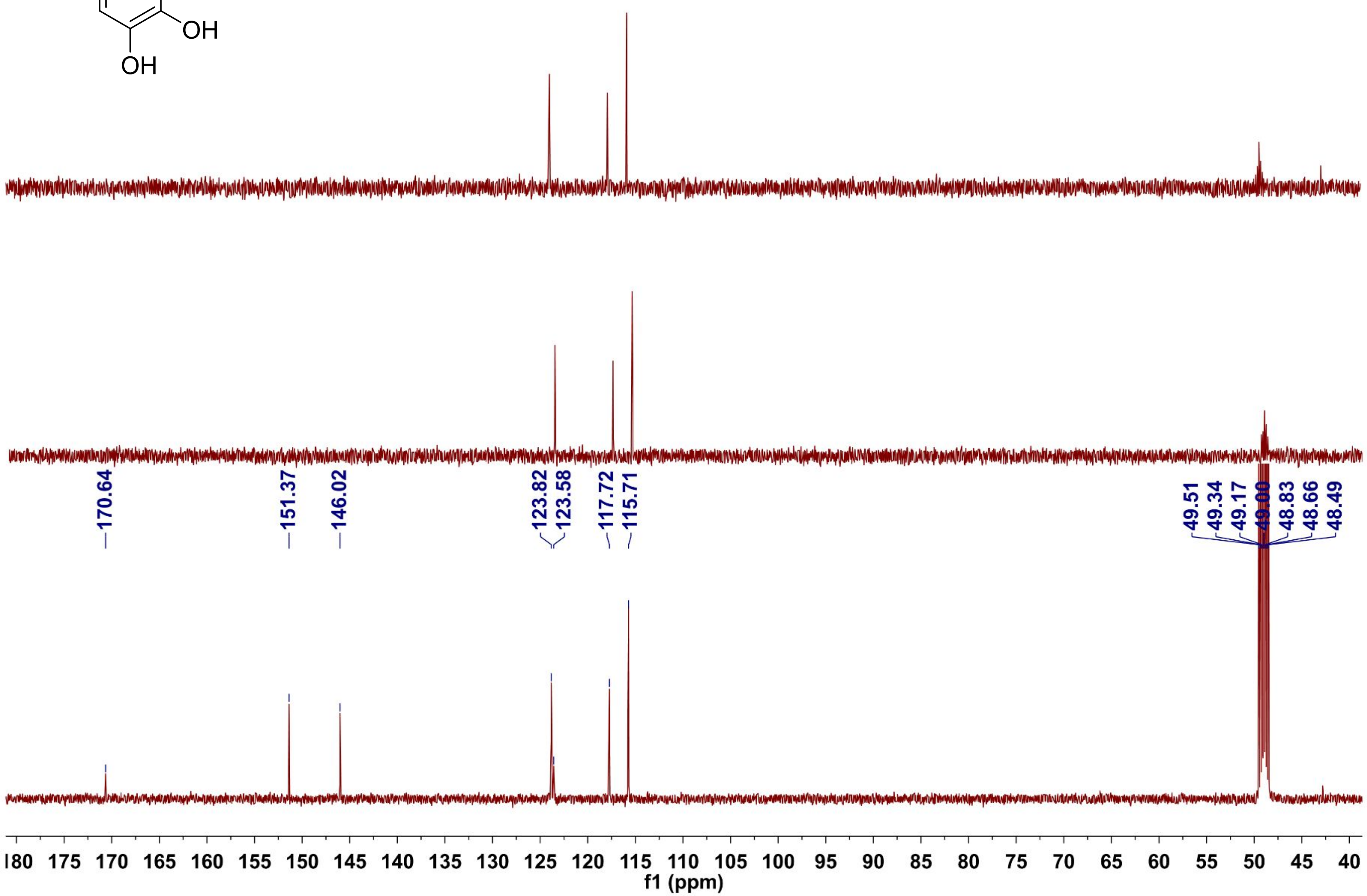

S22-2 ^{13}C NMR spectrum of compound **22** in CD_3OD (125 MHz)

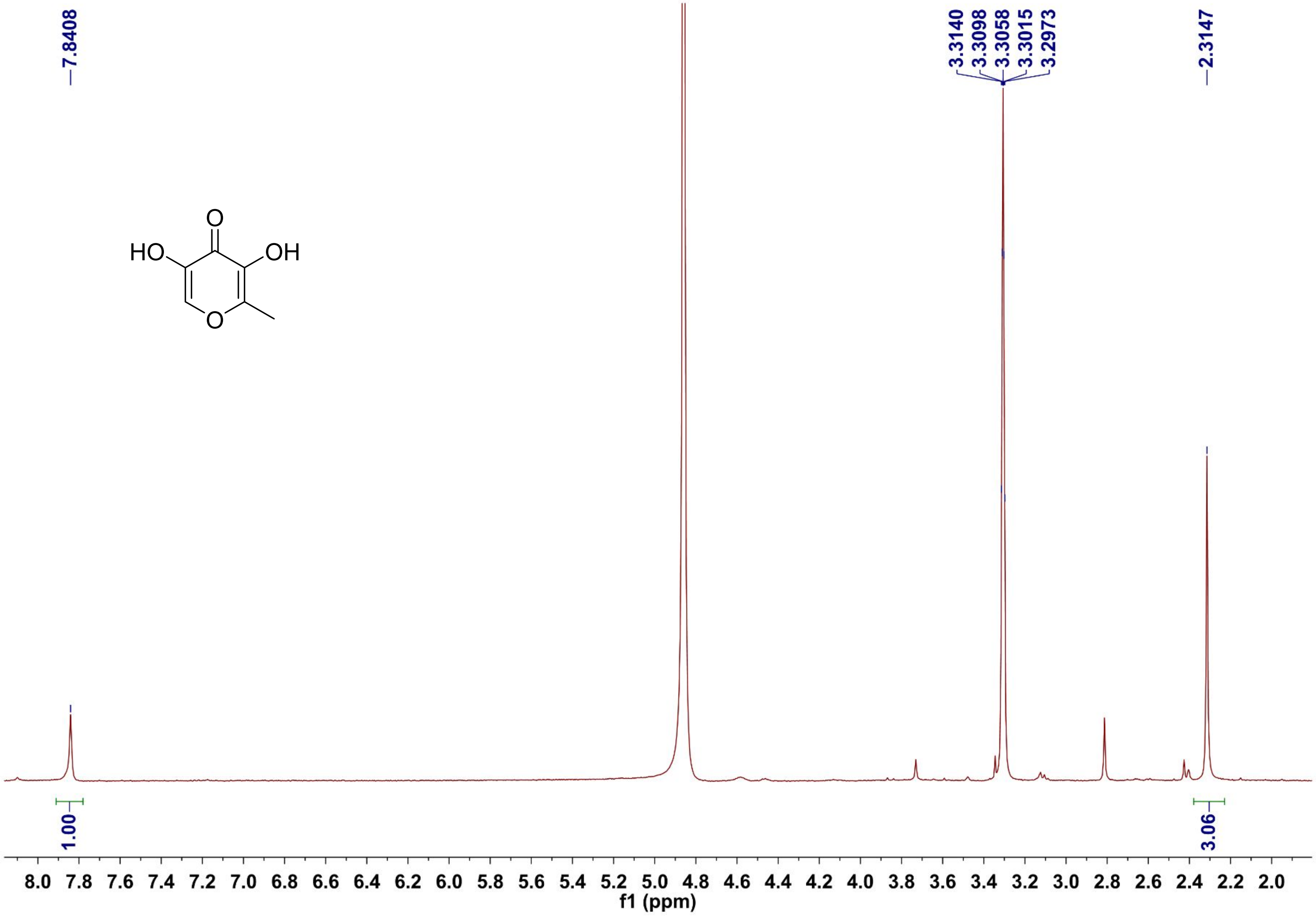

S23-1 ^{1}H NMR spectrum of compound **23** in CD_3OD (400 MHz)

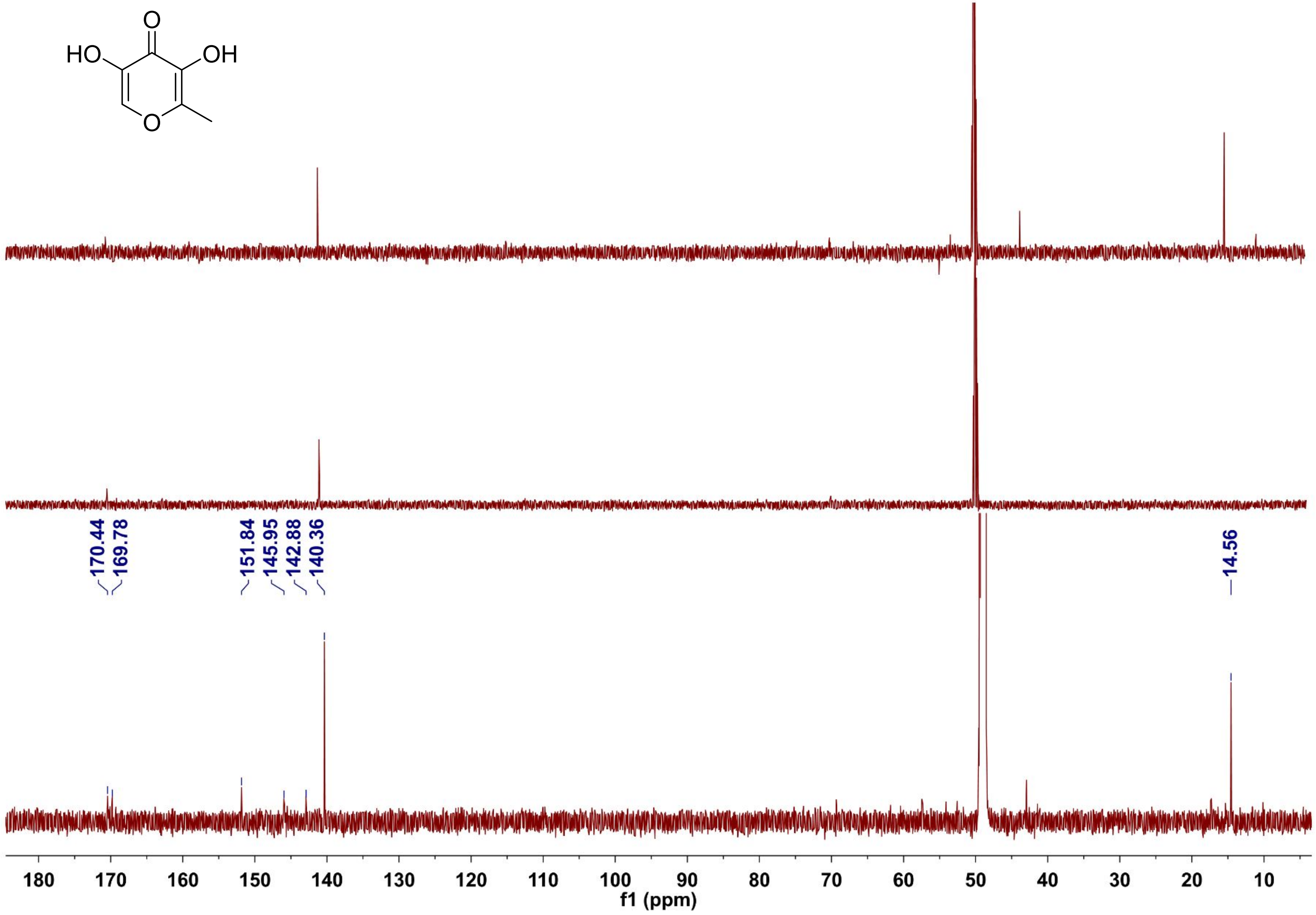

S23-2 ^{13}C NMR spectrum of compound **23** in CD_3OD (150 MHz)

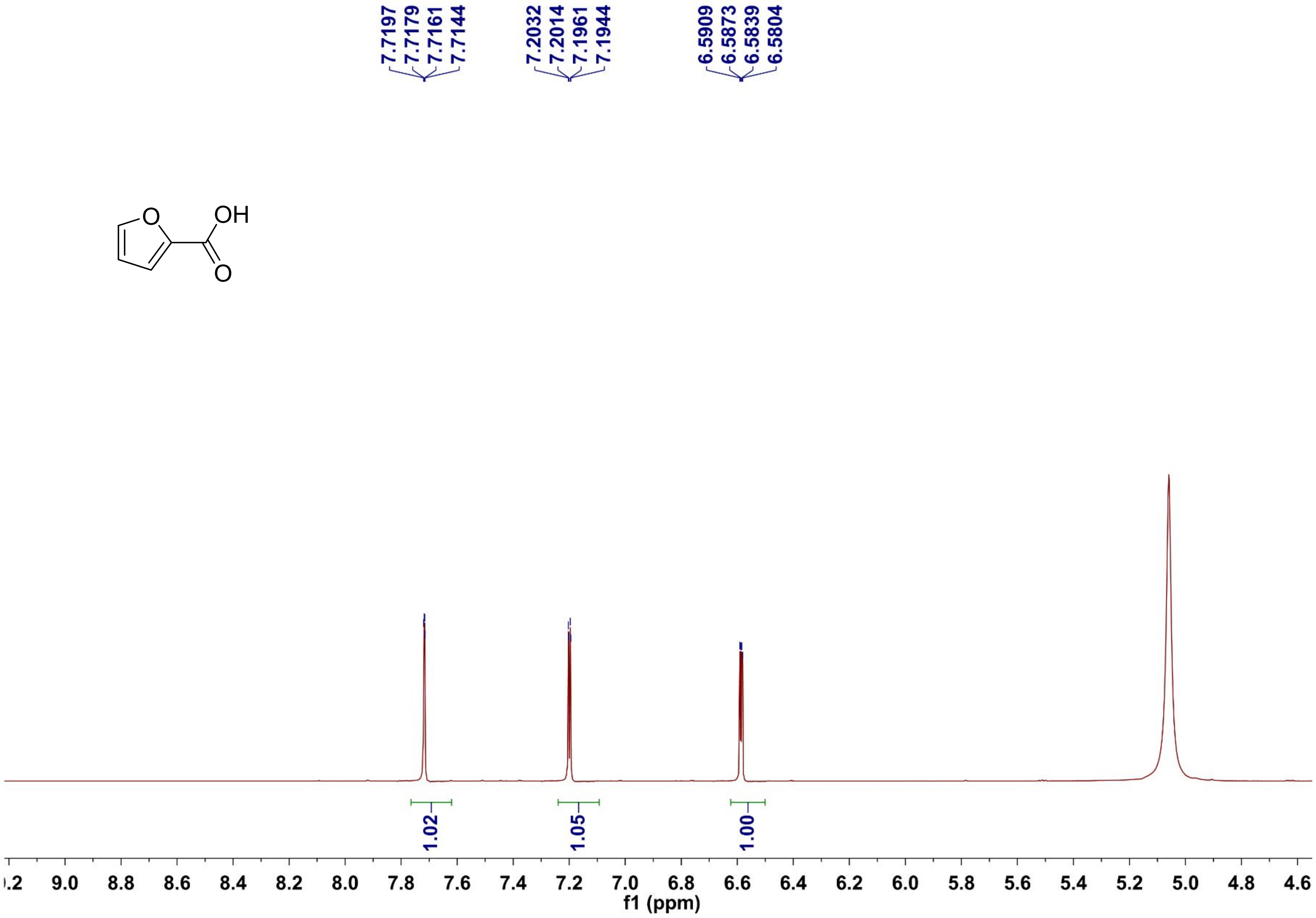

S24-1 ^{1}H NMR spectrum of compound **24** in CD_3OD (500 MHz)

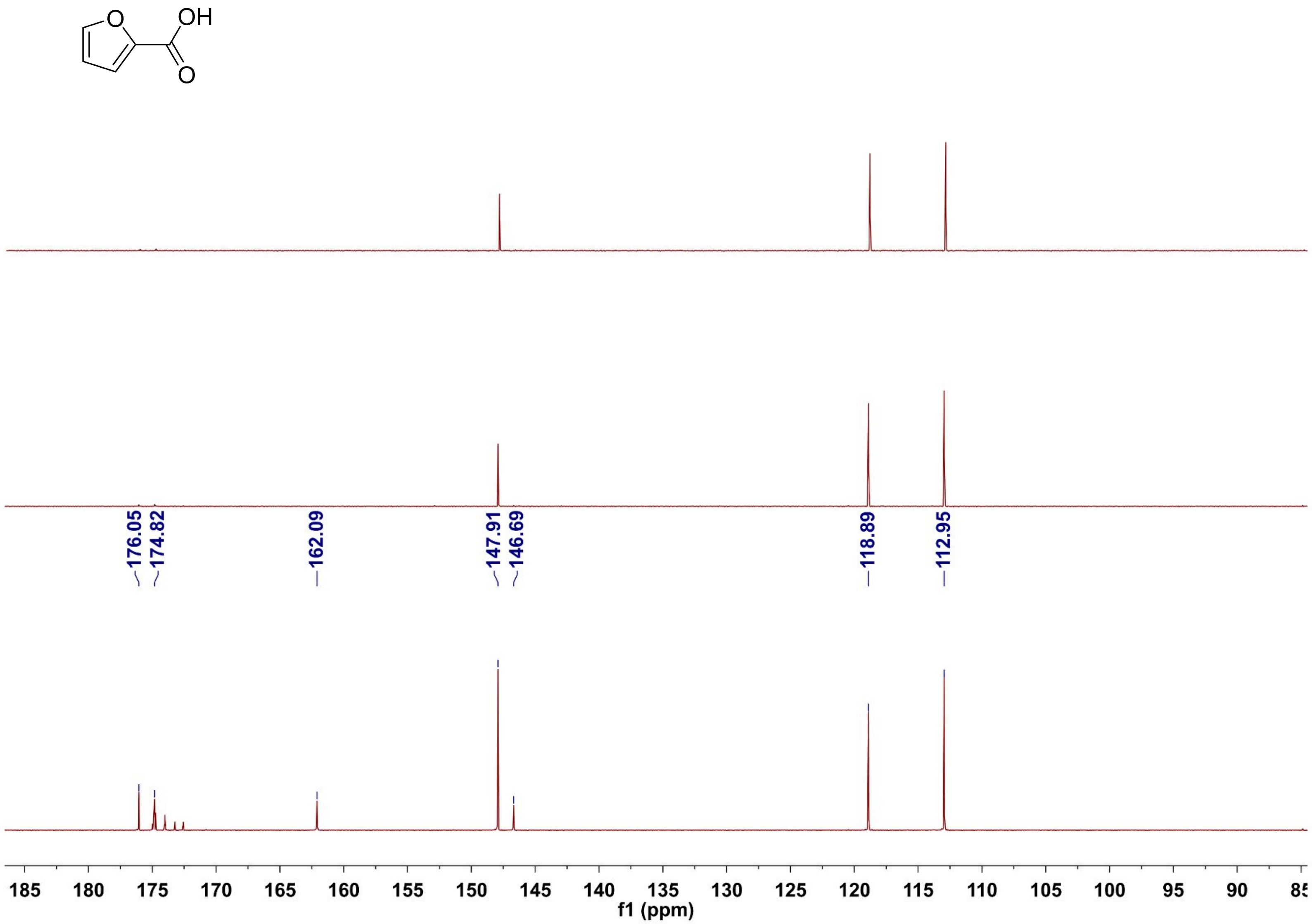

S24-2 ^{13}C NMR spectrum of compound **24** in CD_3OD (125 MHz)

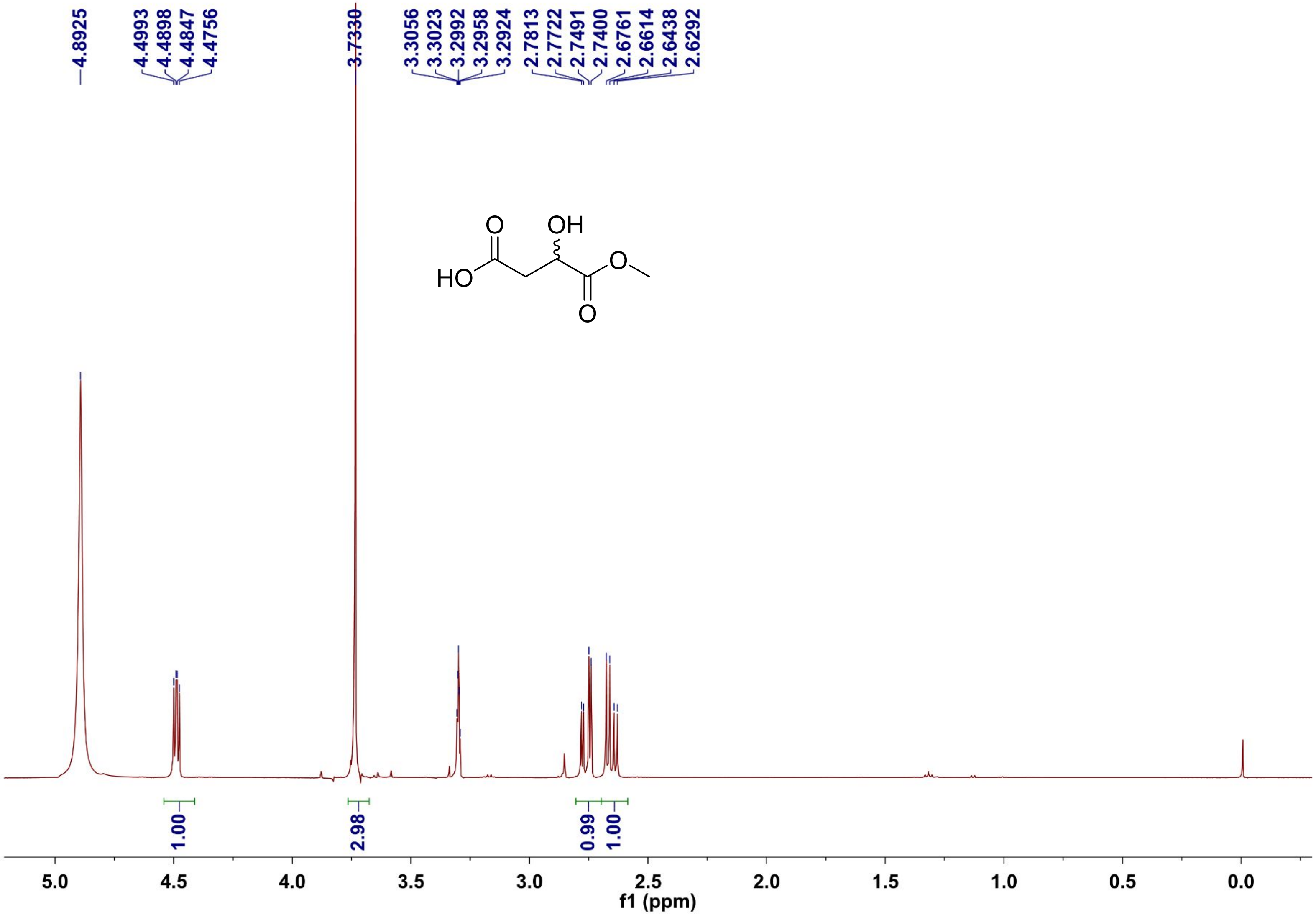

S25-1 ^{1}H NMR spectrum of compound **25** in CD_3OD (500 MHz)

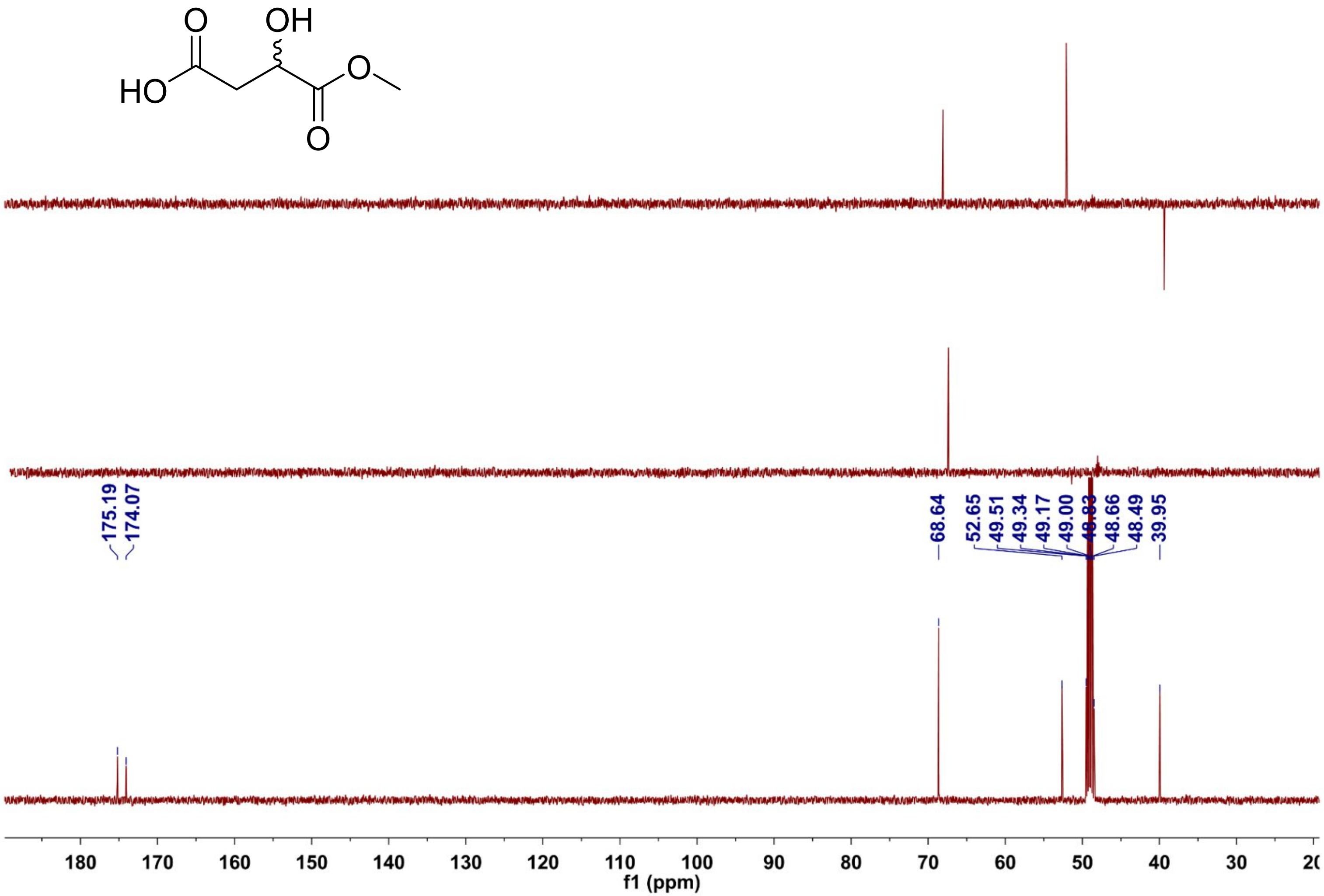

S25-2 ^{13}C NMR spectrum of compound **25** in CD_3OD (125 MHz)

S26. NMR data of compounds 5–25

2-Oxo-pomolic acid (**5**)

$C_{30}H_{46}O_5$; ^{1}H NMR (CD_3OD, 400 MHz): δ 5.29 (1H, br s, H-12), 3.99 (1H, s, H-3), 2.59 (1H, td, J = 13.2, 4.5 Hz, H-16), 2.51 (1H, s, H-18), 2.33 (1H, d, J = 12 Hz, H-1a), 2.23 (1H, d, J = 12 Hz, H-1b), 1.39 (3H, s, CH_3-27), 1.18 (3H, s, CH_3-29), 1.17 (3H, s, CH_3-23), 0.92 (3H, d, J = 6.6 Hz, CH_3-30), 0.88 (3H, s, CH_3-25), 0.79 (3H, s, CH_3-26), 0.71 (3H, s, CH_3-24); ^{13}C NMR (CD_3OD, 125 MHz) : δ 54.3 (C-1), 212.6 (C-2), 83.9 (C-3), 46.5 (C-4), 55.8 (C-5), 19.9 (C-6), 33.8 (C-7), 41.4 (C-8), 48.2 (C-9), 44.5 (C-10), 24.7 (C-11), 128.7 (C-12), 140.2 (C-13), 42.7 (C-14), 29.6 (C-15), 26.5 (C-16), 48.4 (C-17), 55.0 (C-18), 73.5 (C-19), 43.0 (C-20), 27.2 (C-21), 38.9 (C-22), 29.6 (C-23), 17.1 (C-24), 16.5 (C-25), 17.2 (C-26), 24.5 (C-27), 182.2 (C-28), 27.0 (C-29), 17.0 (C-30).

3-*O*-acetyl-pomolic acid (**6**)

$C_{32}H_{50}O_5$;^{1}H NMR (CD_3OD, 400 MHz): δ 5.28 (1H, t, J = 3.7 Hz, H-12), 4.45 (1H, dd, J = 10.8, 4.7 Hz, H-3), 2.56 (1H, td, J = 13.2, 4.5 Hz, H-16), 2.50 (1H, s, H-18), 2.02 (3H, s, C<u>H</u>$_3$COO), 1.34 (3H, s, CH_3-27), 1.18 (3H, s, CH_3-29), 0.98 (3H, s, CH_3-23), 0.92 (3H, d, J = 6.6 Hz, CH_3-30), 0.89 (3H, s, CH_3-25), 0.88 (3H, s, CH_3-24), 0.80 (3H, s, CH_3-26); ^{13}C NMR (CD_3OD, 125 MHz) : δ 39.0 (C-1), 27.2 (C-2), 82.5 (C-3), 39.3 (C-4), 56.7 (C-5), 19.4 (C-6), 34.0 (C-7), 41.0 (C-8), 48.4 (C-9), 38.0 (C-10), 24.6 (C-11), 129.2 (C-12), 140.0 (C-13), 42.5 (C-14), 29.6 (C-15), 24.7 (C-16), 49.0 (C-17), 55.0 (C-18), 73.5 (C-19), 43.0 (C-20), 26.6 (C-21), 38.7 (C-22), 28.5 (C-23), 15.9 (C-24), 16.9 (C-25), 17.4 (C-26), 24.5 (C-27), 182.2 (C-28), 27.0 (C-29), 16.5(C-30), 172.9 (CH_3<u>CO</u>O), 21.1 (<u>CH$_3$</u>COO).

Euscaphic acid (**7**)

$C_{30}H_{48}O_5$; ^{1}H NMR (CD_3OD, 400 MHz): δ 5.29 (1H, t, J = 3.7 Hz, H-12), 3.92 (1H, dt, J = 11.8, 3.8 Hz, H-2), 2.57 (1H, td, J = 13.2, 4.5 Hz, H-16), 2.49 (1H, s, H-18), 1.34 (3H, s, CH_3-27), 1.19 (3H, s, CH_3-29), 0.98 (6H, s, CH_3-23/26), 0.92 (3H, d, J = 6.6 Hz, CH_3-30), 0.86 (3H, s, CH_3-24), 0.78 (3H, s, CH_3-25); ^{13}C NMR (CD_3OD, 125 MHz) : δ 42.7 (C-1), 67.1 (C-2), 80.0 (C-3), 41.2 (C-4), 48.8 (C-5), 25.1 (C-6), 34.0 (C-7), 39.0 (C-8), 48.4 (C-9), 39.4 (C-10), 27.2 (C-11), 129.3 (C-12), 140.0 (C-13), 42.7 (C-14), 29.5 (C-15), 26.5 (C-16), 48.8 (C-17), 55.0 (C-18), 73.5 (C-19), 43.0 (C-20), 27.0 (C-21), 39.0 (C-22), 29.2 (C-23), 22.4 (C-24), 17.5 (C-25), 16.6 (C-26), 27.0 (C-27), 182.3 (C-28), 16.8 (C-29), 24.7 (C-30).

1β-Hydroxy-euscaphic acid (**8**)

$C_{30}H_{48}O_6$; ^{1}H NMR (CD_3OD, 400 MHz): *δ* 5.28 (1H, t, *J* = 3.7 Hz, H-12), 3.64 (1H, dd, *J* = 9.6, 3.1 Hz, H-2), 3.45 (1H, d, *J* = 9.6 Hz, H-3), 3.40 (1H, d, *J* = 3.1 Hz, H-1), 2.48 (1H, s, H-18), 1.35 (3H, s, CH_3-27), 1.19 (3H, s, CH_3-29), 1.00 (3H, s, CH_3-25), 0.96 (3H, s, CH_3-23), 0.92 (3H, d, *J* = 6.6 Hz, CH_3-30), 0.88 (3H, s, CH_3-24), 0.79 (3H, s, CH_3-26); ^{13}C NMR (CD_3OD, 125 MHz) : *δ* 81.2 (C-1), 71.8 (C-2), 80.6 (C-3), 41.7 (C-4), 48.8 (C-5), 19.4 (C-6), 34.1 (C-7), 43.0 (C-8), 48.4 (C-9), 38.9 (C-10), 26.6 (C-11), 130.6 (C-12), 138.8 (C-13), 44.3 (C-14), 29.6 (C-15), 29.0 (C-16), 48.8 (C-17), 54.9 (C-18), 73.5 (C-19), 42.5 (C-20), 27.2 (C-21), 39.0 (C-22), 28.2 (C-23), 22.3 (C-24), 12.9 (C-25), 17.7 (C-26), 24.9 (C-27), 182.3 (C-28), 27.0 (C-29), 16.5(C-30).

2α,3α,19,24-Tetrahydroxy-18,19-seco-urs-11,13(18)-dien-28-oic acid (**9**)

$C_{30}H_{48}O_6$;^{1}H NMR (CD_3OD, 400 MHz): *δ* 5.98 (1H, dd, *J* = 10.1, 2.8 Hz, H-12), 5.66 (1H, d, *J* = 10.1 Hz, H-11), 5.43 (1H, s, H-18), 3.76 (1H, d, *J* = 2.7 Hz, H-3), 3.61 (1H, d, *J* = 11.5 Hz, H-24a), 3.40 (1H, d, *J* = 11.5 Hz, H-24b), 1.08 (3H, s, CH_3-27), 1.08 (3H, s, CH_3-23), 1.07 (3H, d, *J* = 6.6 Hz, CH_3-29), 0.96 (3H, d, *J* = 6.6 Hz, CH_3-30), 0.87 (3H, s, CH_3-27), 0.86 (3H, s, CH_3-25), 0.70 (3H, s, CH_3-26);^{13}C NMR (CD_3OD, 125 MHz) : *δ* 42.3 (C-1), 66.7 (C-2), 74.6 (C-3), 45.4 (C-4), 49.5 (C-5), 19.2 (C-6), 33.6 (C-7), 41.7 (C-8), 55.4 (C-9), 38.8 (C-10), 127.8 (C-11), 131.2 (C-12), 143.5 (C-13), 42.3 (C-14), 27.1 (C-15), 28.1 (C-16), 48.4 (C-17), 129.1 (C-18), 72.0 (C-19), 42.3 (C-20), 28.5 (C-21), 40.1 (C-22), 23.1 (C-23), 65.4 (C-24), 19.3 (C-25), 16.9 (C-26), 19.7 (C-27), 178.6 (C-28), 20.3 (C-29), 15.0 (C-30).

Kajiichigoside F1 (**10**）

$C_{36}H_{58}O_{10}$; ^{1}H NMR (CD_3OD, 400 MHz): *δ* 5.32 (1H, br s, H-1′), 5.30 (1H, br s, H-12), 2.61 (1H, td, *J* = 13.2, 4.5 Hz, H-16), 2.51 (1H, s, H-18), 1.34 (3H, s, CH_3-27), 1.20 (3H, s, CH_3-29), 0.99 (3H, s, CH_3-25), 0.98 (3H, s, CH_3-23), 0.92 (3H, d, *J* = 6.6 Hz, CH_3-30), 0.86 (3H, s, CH_3-26), 0.76 (3H, s, CH_3-24);^{13}C NMR (CD_3OD, 125 MHz) : *δ* 42.5 (C-1), 67.1 (C-2), 80.1 (C-3), 39.4 (C-4), 49.1 (C-5), 22.4 (C-6), 34.0 (C-7), 41.4 (C-8), 48.2 (C-9), 39.4 (C-10), 24.7 (C-11), 129.6 (C-12), 139.6 (C-13), 42.7 (C-14), 29.6 (C-15), 26.5 (C-16), 48.6 (C-17), 54.9 (C-18), 73.6 (C-19), 42.9 (C-20), 27.2 (C-21), 38.3 (C-22), 29.2 (C-23), 16.5 (C-24), 17.1 (C-25), 19.2 (C-26), 24.7 (C-27), 178.5 (C-28), 27.2 (C-29), 17.6 (C-30), 95.7 (C-1′), 73.8 (C-2′), 78.5 (C-3′), 71.1 (C-4′), 78.5 (C-5′), 62.4 (C-6′).

2-Oxo-benthamic acid 28-*O*-β-D-*O*-glucopyranosyl ester (**11**)

$C_{36}H_{56}O_{10}$; ^{1}H NMR (CD_3OD, 400 MHz): δ 5.32 (1H, br s, H-1′), 5.30 (1H, br s, H-12), 3.99 (1H, s, H-3), 2.62 (1H, td, J = 13.2, 4.5 Hz, H-16), 2.52 (1H, s, H-18), 1.38 (3H, s, CH_3-27),

1.19 (3H, s, CH_3-29), 1.17 (3H, s, CH_3-23), 0.92 (3H, d, J = 6.6 Hz, CH_3-30), 0.89 (3H, s, CH_3-25), 0.78 (3H, s, CH_3-26), 0.71 (3H, s, CH_3-24);^{13}C NMR (CD_3OD, 125 MHz) : δ 53.9 (C-1), 212.7 (C-2), 83.9 (C-3), 46.5 (C-4), 55.8 (C-5), 19.9 (C-6), 33.7 (C-7), 41.6 (C-8), 48.3 (C-9), 44.5 (C-10), 24.7 (C-11), 129.0 (C-12), 139.8 (C-13), 42.7 (C-14), 29.6 (C-15), 26.5 (C-16), 48.5 (C-17), 54.9 (C-18), 73.6 (C-19), 42.9 (C-20), 27.2 (C-21), 38.2 (C-22), 29.6 (C-23), 17.1 (C-24), 16.5 (C-25), 17.2 (C-26), 24.5 (C-27), 178.4 (C-28), 27.0 (C-29), 17.0 (C-30), 95.7 (C-1′), 73.8 (C-2′), 78.3 (C-3′), 71.1 (C-4′), 78.5 (C-5′), 62.4 (C-6′).

3.4.12. 2α,3β,19β-Trihydroxy-12-en-urs-onic acid-28-O-β-D-glucopyranosyl ester (**12**)
$C_{36}H_{58}O_{10}$; ^{1}H NMR (DMSO, 400 MHz): δ 5.16 (1H, m, H-1′), 5.15 (1H, m, H-12), 2.74 (1H, dd, J = 9.2, 4.0 Hz, H-3), 2.37 (1H, br s, H-18), 1.27 (3H, s, CH_3-27), 1.09 (3H, s, CH_3-29), 0.92 (3H, s, CH_3-23), 0.90 (3H, s, CH_3-24), 0.84 (3H, d, J = 6.3 Hz, CH_3-30), 0.70 (3H, s, CH_3-25), 0.66 (3H, s, CH_3-26);^{13}C NMR (DMSO, 125 MHz) : δ 47.1 (C-1), 67.1 (C-2), 82.3 (C-3), 40.0 (C-4), 54.8 (C-5), 18.1 (C-6), 32.5 (C-7), 37.5 (C-8), 46.7 (C-9), 38.9 (C-10), 23.3 (C-11), 127.0 (C-12), 138.2 (C-13), 41.1 (C-14), 28.0 (C-15), 25.1 (C-16), 47.3 (C-17), 53.2 (C-18), 71.6 (C-19), 41.2 (C-20), 26.4 (C-21), 36.6 (C-22), 28.8 (C-23), 16.4 (C-24), 17.1 (C-25), 16.5 (C-26), 23.8 (C-27), 175.6 (C-28), 26.4 (C-29), 16.3 (C-30), 94.0 (C-1′), 72.2 (C-2′), 76.7 (C-3′), 69.5 (C-4′), 77.6 (C-5′), 60.6 (C-6′).
2α,3α,19α-Trihydroxy-12-en-oleanolic acid-28-*O*-β-D-glucopyranosyl ester (**13**)
$C_{36}H_{58}O_{10}$; ^{1}H NMR (CD_3OD, 400 MHz): *δ* 5.37 (1H, d, *J* = 8.0 Hz, H-1′), 5.33 (1H, t, *J* = 3.5 Hz, H-12), 3.93 (1H, dt, *J* = 11.8, 3.6 Hz, H-2), 3.04 (1H, br s, H-18), 1.29 (3H, s, CH_3-27), 0.98 (6H, s, CH_3-23/25), 0.94 (3H, s, CH_3-29), 0.93 (3H, s, CH_3-30), 0.86 (3H, s, CH_3-26), 0.76 (3H, s, CH_3-24); ^{13}C NMR (CD_3OD, 125 MHz) : *δ* 42.3 (C-1), 67.1 (C-2), 80.1 (C-3), 39.4 (C-4), 49.4 (C-5), 19.3 (C-6), 33.8 (C-7), 41.0 (C-8), 48.5 (C-9), 39.5 (C-10), 24.9 (C-11), 124.9 (C-12), 144.3 (C-13), 42.7 (C-14), 29.5 (C-15), 28.4 (C-16), 47.1 (C-17), 45.0 (C-18), 82.4 (C-19), 35.9 (C-20), 28.6 (C-21), 33.3 (C-22), 29.4 (C-23), 22.4 (C-24), 16.9 (C-25), 17.9 (C-26), 25.1 (C-27), 178.5 (C-28), 29.4 (C-29), 25.1 (C-30), 95.8 (C-1′), 73.8 (C-2′), 78.2 (C-3′), 71.0 (C-4′), 78.6 (C-5′), 62.3 (C-6′).

(+)-1"-Methylene-6"-hydroxy-2H-furan-5"-one-6-catechin (**14**)
$C_{20}H_{18}O_9$; ^{1}H NMR (CD_3OD, 400 MHz): *δ* 6.77 (1H, d, *J* = 1.5 Hz, H-2′), 6.72 (1H, d, *J* = 8.1 Hz, H-5′), 6.65 (1H, dd, *J* = 8.1, 1.5 Hz, H-6′), 6.02 (1H, s, H-8), 4.68 (1H, d, *J* = 7.5 Hz, H-2), 4.39 (1H, s, 4′′), 3.98 (1H, m, H-3), 3.86 (2H, s, H-1′′), 2.75 (1H, dd, *J* = 16.2, 5.1 Hz, H-4a), 2.52 (1H, dd, *J* = 16.2, 7.2 Hz, H-4b); ^{13}C NMR (CD_3OD, 125 MHz) : *δ* 82.6 (C-2), 68.6 (C-3), 27.7 (C-4), 155.8 (C-5), 100.7 (C-6), 156.1 (C-7), 95.9 (C-8), 154.7 (C-9), 100.6 (C-

10), 132.3 (C-1′), 114.9 (C-2′), 146.1 (C-3′/4′), 116.0 (C-5′), 119.4 (C-6′), 22.7 (C-1′′), 134.9 (C-2′′), 74.1 (C-4′′), 198.2 (C-5′′), 181.0 (C-6′′).

Catechin (**15**)
$C_{15}H_{14}O_6$; ^{1}H NMR (CD_3OD, 400 MHz): δ 6.84 (1H, d, J = 1.6 Hz, H-2′), 6.76 (1H, d, J = 8.1 Hz, H-5′), 6.71 (1H, dd, J = 8.1, 1.6 Hz, H-6′), 5.93 (1H, d, J = 1.9 Hz, H-6), 5.86 (1H, d, J = 1.9 Hz, H-8), 4.57 (1H, d, J = 7.5 Hz, H-2), 3.98 (1H, m, H-3), 2.85 (1H, dd, J = 16.1, 5.4 Hz, H-4a), 2.51 (1H, dd, J = 16.1, 8.0 Hz, H-4b); ^{13}C NMR (CD_3OD, 125 MHz) : δ 82.8 (C-2), 68.8 (C-3), 28.4 (C-4), 157.5 (C-5), 96.3 (C-6), 157.7 (C-7), 95.5 (C-8), 156.9 (C-9), 100.8 (C-10), 132.2 (C-1′), 115.2 (C-2′), 146.2 (C-3′/4′), 115.6 (C-5′), 120.0 (C-6′).

Quercetin (**16**)
$C_{15}H_{10}O_7$;^{1}H NMR (DMSO, 400 MHz): δ 12.54 (1H, s, 5-OH), 7.67 (1H, d, J = 2.1 Hz, H-2′), 7.54 (1H, dd, J = 8.4, 2.1 Hz, H-6′), 6.88 (1H, d, J = 8.4 Hz, H-5′), 6.41 (1H, d, J = 1.9 Hz, H-8), 6.19 (1H, d, J = 1.9 Hz, H-6);^{13}C NMR (DMSO, 125 MHz) : δ 146.7 (C-2), 135.7 (C-3), 175.8 (C-4), 160.7 (C-5), 98.1 (C-6), 163.9 (C-7), 93.6 (C-8), 156.1 (C-9), 103.0 (C-10), 121.9 (C-1′), 115.0 (C-2′), 145.0 (C-3′), 147.7 (C-4′), 115.6 (C-5′), 119.9 (C-6′).

Tiliroside (**17**)
$C_{30}H_{26}O_{12}$;^{1}H NMR (CD_3OD, 400 MHz): δ 7.99 (2H, d, J = 8.6 Hz, H-2′/6′), 7.39 (1H, d, J = 15.9 Hz, H-β), 7.31 (2H, d, J = 8.4 Hz, H-2′′′/6′′′), 6.80 (4H, m, H-3′/5′, 3′′′/5′′′), 6.31 (1H, s, H-8), 6.13 (1H, s, H-6), 6.07 (1H, d, J = 15.9 Hz, H-γ);^{13}C NMR (CD_3OD, 125 MHz) : δ 159.3 (C-2), 135.1 (C-3), 179.4 (C-4), 162.9 (C-5), 99.9 (C-6), 165.9 (C-7), 94.7 (C-8), 158.4 (C-9), 105.5 (C-10), 122.7 (C-1′), 132.2 (C-2′/6′), 116.0 (C-3′/5′), 161.5 (C-4′), 103.8 (C-1′′), 75.7 (C-2′′), 75.6 (C-3′′), 71.7 (C-4′′), 77.9 (C-5′′), 64.2 (C-6′′), 127.0 (C-1′′′), 131.1 (C-2′′′/6′′′), 116.7 (C-3′′′/5′′′), 161.1 (C-4′′′), 168.7 (Cα), 114.7 (Cβ), 146.5 (Cγ).

1-[(*E*)-3-(4-hydroxy-phenyl)-2-propenoate β-D-glucopyranosyl ester] (**18**)
$C_{15}H_{18}O_8$;^{1}H NMR (CD_3OD, 400 MHz): δ 7.72 (1H, d, J = 15.9 Hz, H-7), 7.47 (2H, d, J = 8.5 Hz, H-2/6), 6.81 (2H, d, J = 8.5 Hz, H-3/5), 6.36 (1H, d, J = 15.9 Hz, H-8), 5.57 (1H, d, J = 7.7 Hz, H-1′);^{13}C NMR (CD_3OD, 125 MHz) : δ 127.0 (C-1), 131.3 (C-2/6), 116.8 (C-3/5), 161.5 (C-4), 147.9 (C-7), 114.4 (C-8), 167.7 (C-9), 95.7 (C-1′), 74.0 (C-2′), 78.0 (C-3′), 71.1 (C-4′), 78.8 (C-5′), 62.3 (C-6′).

1-Feruloyl-β-D-glucopyranoside (**19**)

$C_{16}H_{20}O_9$; ^{1}H NMR (CD_3OD, 500 MHz): δ 7.72 (1H, d, J = 15.9 Hz, H-7), 7.20 (1H, d, J = 1.8 Hz, H-2), 7.09 (1H, dd, J = 8.2, 1.8 Hz, H-6), 6.81 (1H, d, J = 8.2 Hz, H-5), 6.39 (1H, d, J = 15.9 Hz, H-8), 5.56 (1H, d, J = 7.8 Hz, H-1′), 3.88 (3H, s, -OCH_3); ^{13}C NMR (CD_3OD, 125 MHz) : δ 127.5 (C-1), 111.8 (C-2), 149.4 (C-3), 150.9 (C-4), 114.7 (C-5), 124.3 (C-6), 148.2 (C-7), 116.5 (C-8), 167.6 (C-9), 95.7 (C-1′), 74.0 (C-2′), 78.0 (C-3′), 71.1 (C-4′), 78.8 (C-5′), 62.3 (C-6′), 56.4 (-OCH_3).

Methyl-(*E*)-p-coumarate (**20**)

$C_{10}H_{10}O_3$;^{1}H NMR (CD_3OD, 400 MHz): δ 7.61 (1H, d, J = 16.0 Hz, H-7), 7.45 (2H, d, J = 8.3 Hz, H-3/5), 6.80 (1H, d, J = 8.3 Hz, H-2/6), 6.32 (1H, d, J = 16.0 Hz, H-8), 3.76 (3H, s, $COOCH_3$); ^{13}C NMR (CD_3OD, 150 MHz): δ 161.3 (C-1), 116.8 (C-2/6), 131.4 (C-3/5), 127.1 (C-4), 146.5 (C-7), 114.8 (C-8), 169.7 ($\underline{CO}OCH_3$), 51.9 ($COO\underline{C}H_3$).

Gallic acid（**21**）

$C_7H_6O_5$;^{1}H NMR (CD_3OD, 400 MHz): δ 7.70 (2H, s, H-2/6); ^{13}C NMR (CD_3OD, 125 MHz): δ 122.2 (C-1), 110.3 (C-2/6), 146.3 (C-3/5), 139.4 (C-4), 170.6 (C-7).

Protocatechuic acid (**22**)

$C_7H_6O_4$;^{1}H NMR (CD_3OD, 400 MHz): δ 7.43 (1H, brs, H-2), 7.42 (1H, d, J = 8.0 Hz, H-6), 6.79 (1H, d, J = 8.0 Hz, H-5); ^{13}C NMR (CD_3OD, 125 MHz): δ 123.5 (C-1), 115.7 (C-2), 146.0 (C-3), 151.3 (C-4), 117.7 (C-5), 123.8 (C-6), 170.6 (COOH).

5-Hydroxymaltol (**23**)

$C_6H_6O_4$; ^{1}H NMR (CD_3OD, 400 MHz): δ 7.84 (1H, s, H-2), 2.31 (3H, s, CH_3);^{13}C NMR (CD_3OD, 150 MHz): δ 140.3 (C-2), 151.8 (C-3), 170.4 (C-4), 142.9 (C-5), 145.9 (C-6), 14.5 (CH_3).

Furan-2-carboxylic acid (**24**)

$C_5H_4O_3$; ^{1}H NMR (CD_3OD, 400 MHz): δ 7.71 (1H, brs, H-3), 7.20 (1H, d, J = 3.3 Hz, H-5), 6.58 (1H, t, J = 1.7 Hz, H-4); ^{13}C NMR (CD_3OD, 125 MHz): δ 146.6 (C-2), 118.8 (C-3), 112.9 (C-4), 147.9 (C-5), 162.0 (C-6).

2-Hydroxy-butandioic acid-1-methyl ester (**25**)

$C_5H_8O_5$;^{1}H NMR (CD_3OD, 400 MHz): δ 4.49 (1H, dd, J = 7.2, 4.7 Hz, H-2), 3.74 (3H, s, -OCH_3), 2.76 (1H, dd, J = 16.1, 4.7 Hz, H-3), 2.65 (1H, dd, J = 16.1, 7.2 Hz, H-3); ^{13}C NMR (CD_3OD, 125 MHz): δ 175.1 (C-1), 68.6 (C-2), 39.9 (C-3), 174.0 (C-4), 52.6 (-OCH_3).

S27. Quantification by LC-MS of compounds 3, 5, 15, 16, 21 and 22

Compound **3**

Coefficient of Determination: R^2 = 0.999969

Calibration curve: 83.1073 * x

Response type: External Std, Area

Curve type: Linear, Origin: Force, Weighting: Null, Axis trans: None

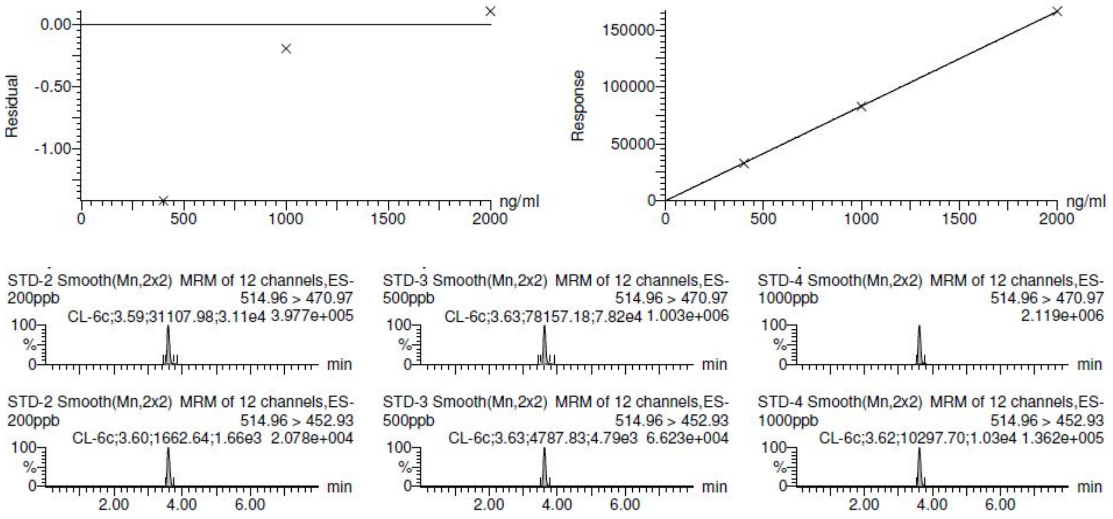

Figure S1 Standard curve for compound **3**

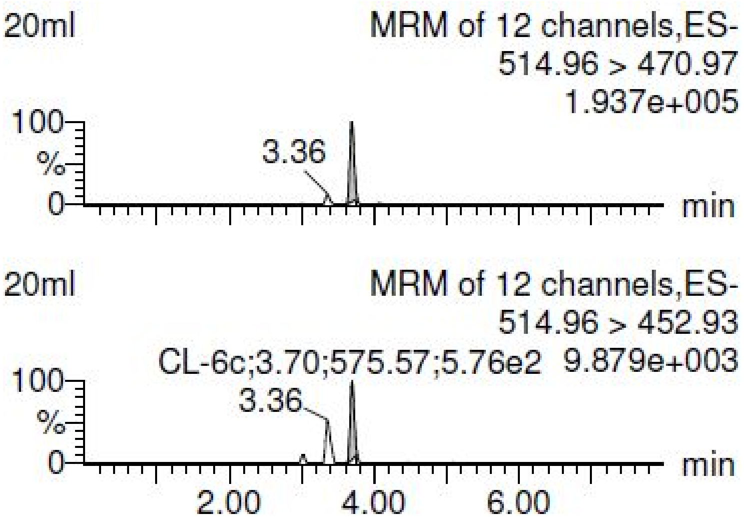

Figure S2 Ion flow diagram of compound **3** in total extract

Table S1 Standard concentration (μg mL^{-1}) and content determination of compound **3**

Name	Std.conc	RT	Area	Response	ng mL^{-1}	%Dev
STD2	400.000	3.59	31107.977	32770.617	394.3	-1.4

STD3	1000.000	3.63	78157.180	82945.013	998.0	-0.2
STD4	2000.000	3.62	156092.594	166390.297	2002.1	0.1
Extract1		3.70	12217.538	12793.105	153.9	

Compound **5**

Coefficient of Determination: $R^2 = 0.999561$

Calibration curve: 41.3092 * x

Response type: External Std, Area

Curve type: Linear, Origin: Force, Weighting: Null, Axis trans: None

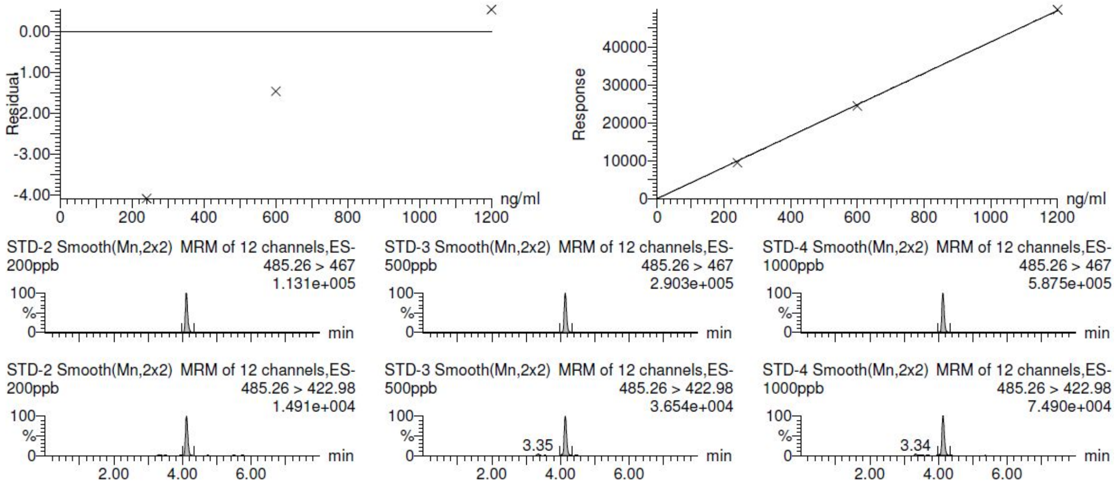

Figure S3 Standard curve for compound **5**

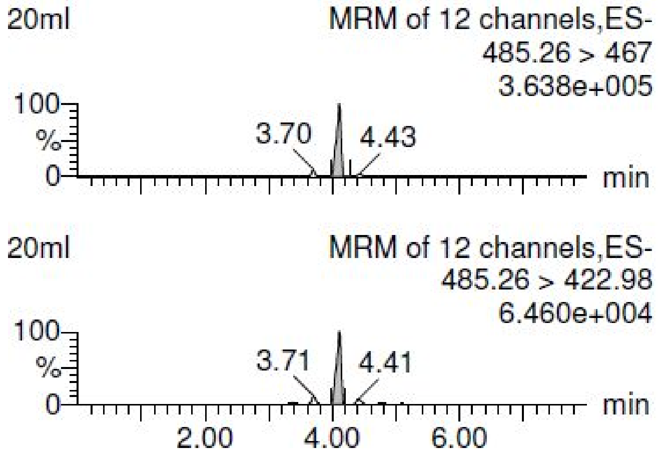

Figure S4 Ion flow diagram of compound **5** in total extract

Table S2 Standard concentration (μg mL^{-1}) and content determination of compound **5**

Name	Std.conc	RT	Area	Response	ng mL^{-1}	%Dev
STD2	240.000	4.11	8376.034	9509.820	230.2	-4.1
STD3	600.000	4.14	21611.975	24421.727	591.2	-1.5
STD4	1200.000	4.13	43868.320	49833.830	1206.4	0.5
Extract1		4.11	30456.217	35934.800	869.9	

Compound **15**

Coefficient of Determination: R^2 = 0.999376

Calibration curve: 2.38155 * x

Response type: External Std, Area

Curve type: Linear, Origin: Force, Weighting: Null, Axis trans: None

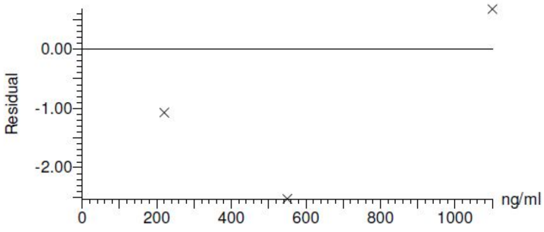

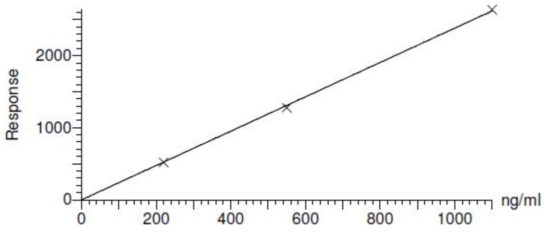

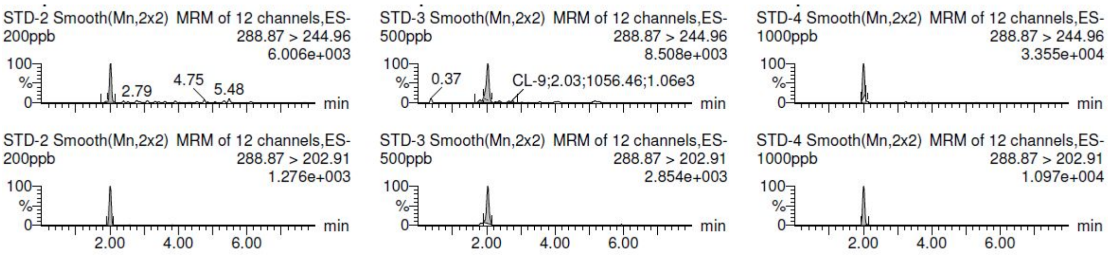

Figure S5 Standard curve for compound **15**

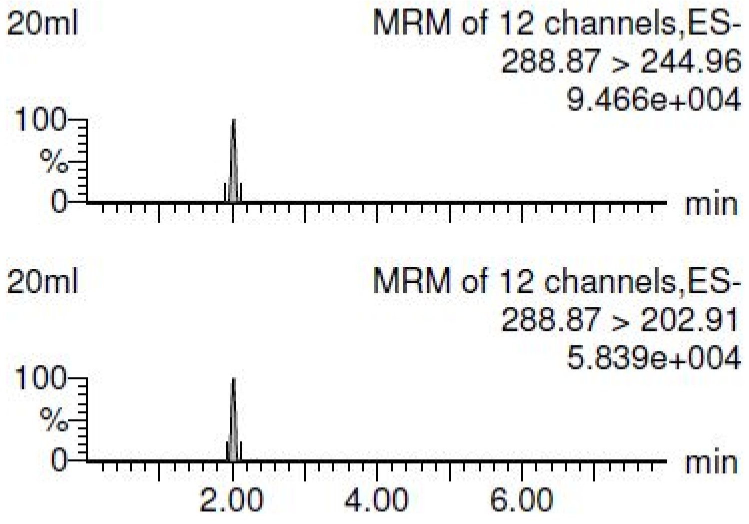

Figure S6 Ion flow diagram of compound **15** in total extract

Table S3 Standard concentration (μg mL^{-1}) and content determination of compound **15**

Name	Std.conc	RT	Area	Response	ng mL^{-1}	%Dev
STD2	220.000	2.02	431.469	518.327	217.6	-1.1
STD3	550.000	2.03	1056.465	1276.772	536.1	-2.5
STD4	1100.000	2.01	1862.840	2637.375	1107.4	0.7
Extract1		2.02	5662.145	9084.355	3814.5	

Compound **16**

Coefficient of Determination: R^2 = 0.999143

Calibration curve: 2.81434 * x

Response type: External Std, Area

Curve type: Linear, Origin: Force, Weighting: Null, Axis trans: None

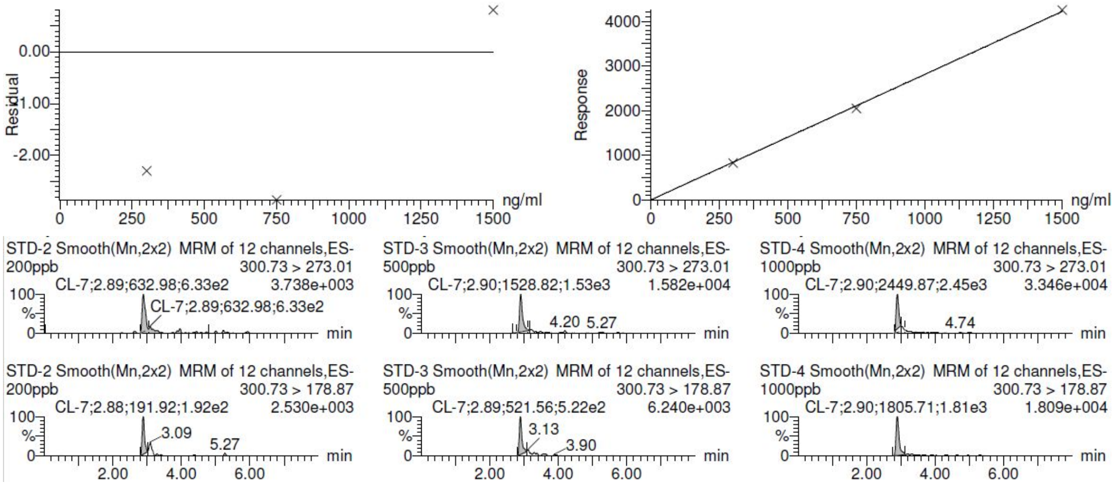

Figure S7 Standard curve for compound **16**

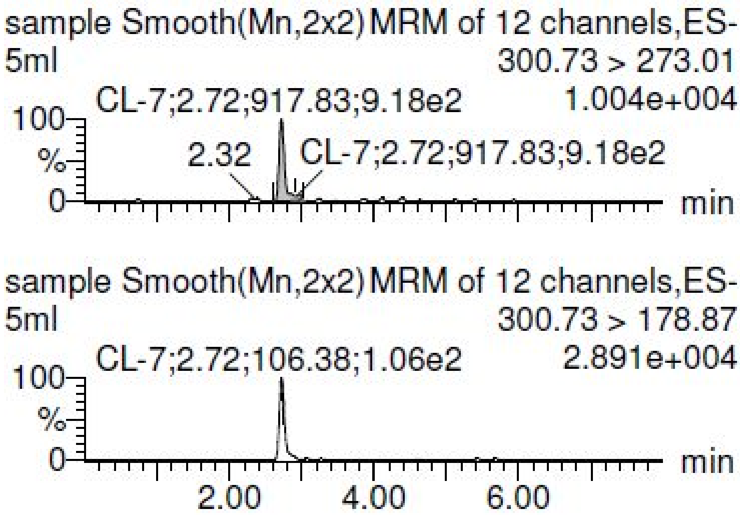

Figure S8 Ion flow diagram of compound **16** in total extract

Table S4 Standard concentration ($\mu g\ mL^{-1}$) and content determination of compound **16**

Name	Std.conc	RT	Area	Response	ng mL^{-1}	%Dev
STD2	300.000	2.89	632.984	824.904	293.1	-2.3
STD3	750.000	2.90	1528.817	2050.379	728.5	-2.9
STD4	1500.000	2.90	2449.870	4255.578	1512.1	0.8
Extract1		2.72	917.832	1024.216	363.9	

Compound **21**

Coefficient of Determination: R^2 = 0.999805

Calibration curve: 24.781 * x

Response type: External Std, Area

Curve type: Linear, Origin: Force, Weighting: Null, Axis trans: None

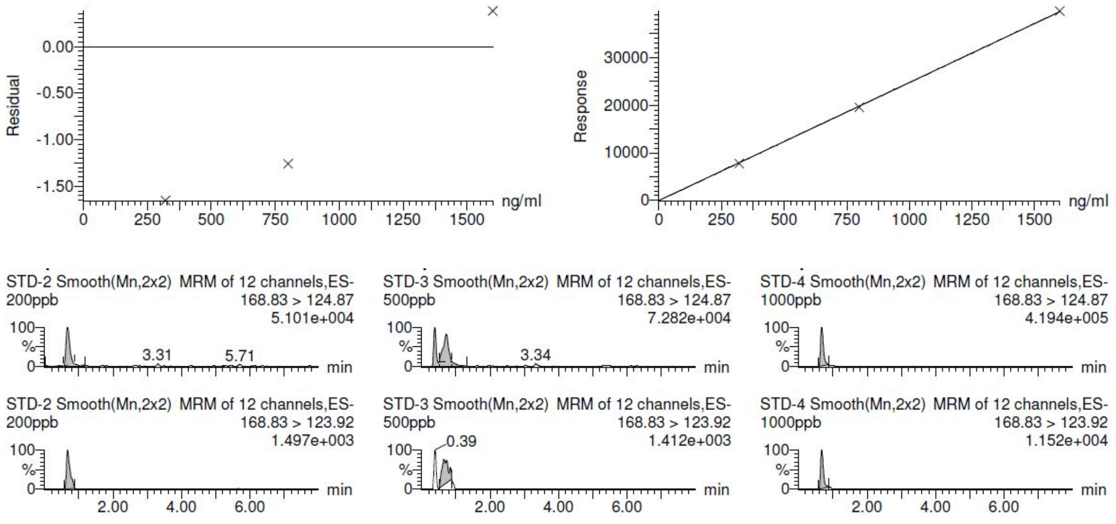

Figure S9 Standard curve for compound **21**

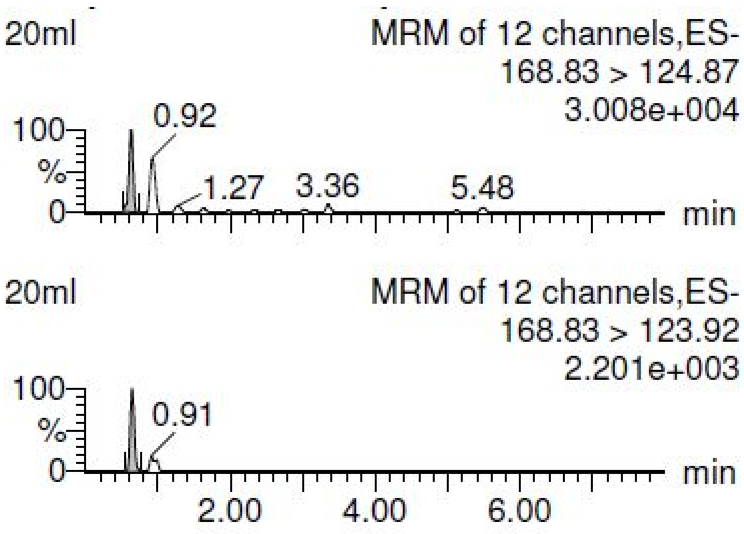

Figure S10 Ion flow diagram of compound **21** in total extract

Table S5 Standard concentration (μg mL^{-1}) and content determination of compound **21**

Name	Std.conc	RT	Area	Response	ng mL^{-1}	%Dev

STD2	320.000	0.67	7622.824	7798.686	314.7	-1.7
STD3	800.000	0.38	19378.420	19575.028	789.9	-1.3
STD4	1600.000	0.67	38774.488	39800.645	1606.1	0.4
Extract1		0.63	1966.696	2110.207	85.2	

Compound **22**

Coefficient of Determination: R^2 = 0.999168

Calibration curve: 21.6481 * x

Response type: External Std, Area

Curve type: Linear, Origin: Force, Weighting: Null, Axis trans: None

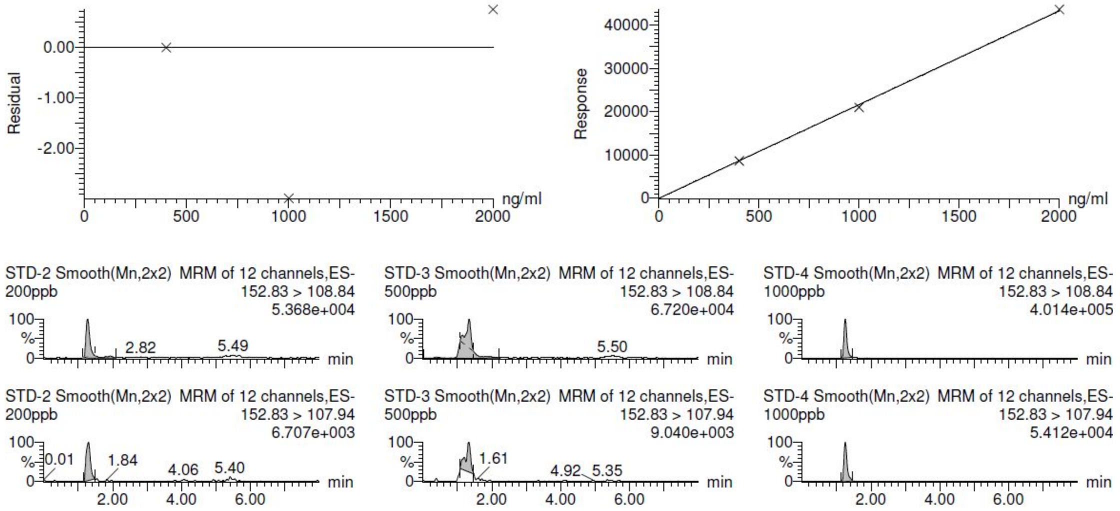

Figure S11 Standard curve for compound **22**

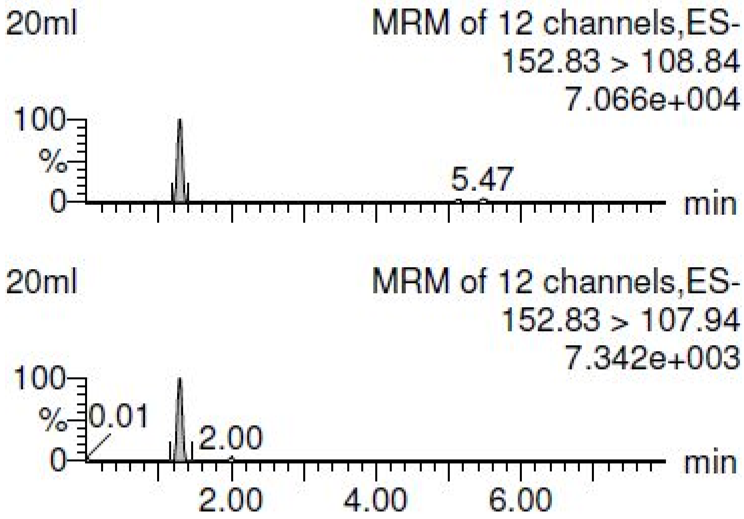

Figure S12 Ion flow diagram of compound **22** in total extract

Table S6 Standard concentration (μg mL^{-1}) and content determination of compound **22**

Name	Std.conc	RT	Area	Response	ng mL^{-1}	%Dev
STD2	400.000	1.27	7741.212	8658.749	400.0	-0.0
STD3	1000.000	1.34	19866.834	21001.347	970.1	-3.0
STD4	2000.000	1.25	38117.488	43619.726	2014.9	0.7
Extract1		1.29	5450.314	6037.218	278.9	
